# A Randomised Feasibility Trial of a Service‐Coordinated Exercise Intervention in First‐Episode Psychosis: Challenges in Implementation and Outcome Assessment

**DOI:** 10.1111/eip.70193

**Published:** 2026-07-09

**Authors:** Brian W. O'Mahony, Elizabeth Wycherley, Karen Daly, Meghan Cowman, Edgar Lonergan, Abigale Owens, Karen O'Mahony, Brian O'Donoghue, Gerard Clarke, Karen O'Connor

**Affiliations:** ^1^ UCD Centre for Psychosis Research, School of Medicine University College Dublin Dublin Ireland; ^2^ RISE, Blackrock Hall Primary Care Centre Cork Ireland; ^3^ Department of Psychiatry St Vincent's University Hospital Dublin Ireland; ^4^ Department of Psychiatry and Neurobehavioural Science University College Cork Cork Ireland; ^5^ APC Microbiome Ireland Biosciences Research Institute, University College Cork Cork Ireland

**Keywords:** attrition, exercise, feasibility, first‐episode psychosis, metabolic health, physical health

## Abstract

**Background:**

Psychosis is increasingly recognised as a systemic disorder characterised by interrelated metabolic dysfunction, chronic low‐grade inflammation and sedentary lifestyles. Physical exercise is a promising intervention to address this triad. This pilot study investigated the feasibility and systemic impact of a pragmatic exercise intervention within a first‐episode psychosis (FEP) service.

**Methods:**

We conducted a parallel‐group randomised controlled trial involving people with FEP, comparing an 8‐week structured aerobic and resistance training programme to Treatment As Usual. We assessed feasibility of the intervention and assessments by measuring recruitment, attendance, retention, and engagement with assessments. Exploratory outcome measures spanned physical fitness, metabolic health, psychometric scales and the Cambridge Neuropsychological Test Automated Battery (CANTAB) cognitive battery.

**Results:**

The study recruited 40 participants, 19 in the intervention and 21 in the control group. At baseline assessment, 23 of 38 had an elevated BMI and 24 of 35 had elevated cholesterol. Study retention was adequate, with only four participants (10%) lost to follow up. However, the assessment battery was poorly completed, highlighting its infeasibility. Fitness outcomes proved especially challenging, with between 11 and 13 participants (27.5% to 32.5%) completing both baseline and endpoint assessments. Of the 19 people recruited to the intervention arm, 7 (36.8%) attended less than half of the 8 sessions. Outcome data were limited by substantial missingness, with no consistent pattern of benefit or harm observed across domains.

**Conclusions:**

Implementing exercise within real‐world FEP services is challenging as indicated by the attrition observed in our study. Future research should individualise exercise prescription and minimise participant assessment burden to mitigate attrition.

## 1 Introduction

High rates of early mortality in people with psychosis are well recognised, with an estimated 15‐year reduction in life expectancy (Laursen 2011; Plana‐Ripoll et al. 2019; Hjorthøj et al. 2017). Psychosis can be seen as a systemic disorder, with affected individuals showing evidence of metabolic dysfunction and inflammation upon first presentation to services (Pillinger et al. 2019; Perry et al. 2016; Dunleavy et al. 2022). This is further exacerbated by the weight gain and metabolic dysregulation caused by antipsychotic medication (Pillinger et al. 2020), as well as other lifestyle factors, such as increased sedentary behaviour and high calorie, low nutritional diets (Manzanares et al. 2014; Vancampfort et al. 2017). These factors lead to high rates of cardiometabolic morbidity in people with severe mental illness, with approximately one‐third experiencing metabolic syndrome (Vancampfort et al. 2015).

Psychosis has also increasingly been correlated with systemic inflammation (Perkins et al. 2025; Momtazmanesh et al. 2019; Halstead et al. 2023), and longitudinal studies suggest that inflammation precedes the development of psychosis (Khandaker et al. 2014; Palmer et al. 2024; Perry et al. 2021). This cohort thus faces challenges in metabolic function, inflammation and an unhealthy lifestyle, all of which are interlinked mechanistically (Firth et al. 2019). Exercise is a plausible intervention to positively impact each of these three challenges. Physical activity is well recognised to improve metabolic function and reduce inflammation (Donnelly et al. 2009; Esteves and Stanford 2024; Thyfault and Bergouignan 2020; Magni et al. 2025). Exercise has been shown to positively impact positive and negative symptoms of psychosis (Ziebart et al. 2022; Rißmayer et al. 2024; Kim et al. 2023), and also improves the cognitive function of people with schizophrenia (Firth et al. 2017; Subotnik et al. 2025). High intensity interval training offers a pragmatic group‐based supervised mode of exercise and has been shown to have beneficial effects on inflammation and metabolic function (Khalafi and Symonds 2020).

Implementing lifestyle interventions for people with psychosis is challenging (Teasdale et al. 2025), not least because of the biopsychological and sociodemographic challenges faced by this cohort (James et al. 2025; Arnautovska et al. 2022). This is especially pronounced in the clinical setting, where systemic barriers such as limited staffing, lack of integrated metabolic monitoring, and the prioritisation of acute treatment of symptoms often sideline long‐term physical health goals (Firth et al. 2019). There is also an imperative to evaluate the acceptability, tolerability and effectiveness of exercise interventions in improving physical health in people with a first episode of psychosis, as there has been limited research in this area (Ancín‐Osés et al. 2025). Attendance of interventions in a research setting would likely exceed that of a clinical service, who would not have staff dedicated to intervention retention and would likely face more challenging staff: patient ratios. The vast majority of FEP services worldwide lack an embedded exercise professional on their multidisciplinary team, and other clinical staff may not have the same proficiency in motivating exercise attendance and ensuring optimisation.

Additionally, research is needed to find the optimal level of measurements included in this research. Although it may yield important information, completing anthropometric measurements and research assessments places an additional burden on both busy clinical staff and a cohort who have high rates of dropout in such trials (Vancampfort et al. 2016). Therefore, a fine balance needs to be achieved between measuring essential outcomes and acceptability to participants and staff.

We aimed primarily to assess the feasibility of implementing a pragmatic, service‐coordinated exercise intervention and secondarily to explore signals across metabolic, cognitive and clinical domains to inform future trials. Preliminary evidence of such a multi‐modal approach which simultaneously addressed the triad of metabolic dysfunction, systemic inflammation and symptomatic burden would help inform the development of more integrated cardiovascular‐focused care.

## 2 Methods

### 2.1 Design

The study was a parallel‐group randomised controlled trial, with the intervention group receiving a structured 8‐week exercise programme. The primary objective was feasibility (recruitment, retention, engagement and intervention adherence). Secondary objectives were exploratory and not powered to detect efficacy.

The programme consisted of weekly 60‐minute supervised group sessions of combined aerobic and resistance training and encouragement to complete individual training at home. The control group received treatment as usual (TAU) from their multidisciplinary service, including medical, psychology, nursing, occupational therapy and dietician reviews. The control group also had the opportunity to take part in the same 8‐week exercise programme after the study period was complete.

### 2.2 Ethical Approval

The Clinical Research Ethics Committee of the Cork Teaching Hospitals (CREC) granted ethical approval for the study (reference: ECM 4 (x) 13/12/2022) and each participant provided informed consent.

### 2.3 Participants

Participants were aged between 18 and 65 and had received a diagnosis of first‐episode psychosis (FEP) in the past 3 years. We excluded any participants who were deemed unable to give informed consent, pregnant females and people with severe cardiovascular disease. We also excluded people who had, in the previous 2 weeks, experienced respiratory tract infection symptoms, symptoms of COVID‐19, or diarrhoea, and people who had taken antibiotics at any point in the previous 4 weeks, as the trial had initially intended to evaluate the impact of the intervention on the gut microbiome. We recruited people with a diagnosis of FEP from two outpatient early intervention for psychosis services in Cork, Ireland: RISE (Responsive early Intervention for psychosis SErvice) in South Lee Mental Health Services and EIST (Early Intervention Screening and Treatment) in North Lee Mental Health Services.

### 2.4 Intervention

Qualified exercise professionals delivered the intervention in the Mardyke Arena, University College Cork, a university gym at least two kilometres from both clinics. Participants from both groups did not attend shared intervention activities, and exercise sessions were only available to intervention participants. The participants completed three circuits of high‐intensity interval type training, with time splits of 60 s on, 20 s off; 45 s on, 15 s off; and 30 s on, 20 s off successively. The circuits contained a mixture of aerobic exercises (treadmill, exercise bike, cross trainer, rowing machine, skiing machine), free weights, machine weights and kettlebells. The session supervisors used the principle of progressive overload but remained flexible to the needs of each individual participant. Participants received a reminder the day before the upcoming exercise session.

The 8‐week intervention was delivered to five blocks of participants in the intervention group. The first session was delivered on 11 October 2023, and the last session was delivered on 16 April 2025.

In contrast, TAU consisted of routine care provided by the Early Intervention in Psychosis (EIP) service, delivered by a multidisciplinary team. Care was individualised according to clinical need and typically included psychiatric review, medication management, keyworker support, psychological interventions, family support, employment support, physical health screening and lifestyle advice, and referral to other allied health services where indicated. Participants did not have access to a dietician. Frequency of contact was weekly or fortnightly depending on illness phase and stage of recovery.

### 2.5 Feasibility Metrics

We assessed feasibility through four metrics:

Recruitment: The number of patients from the two FEP services who consented to take part in the study.

Retention: The number of participants who attended for the endpoint assessment.

Engagement (of assessment): The number of baseline and/or endpoint variables completed by a participant.

Intervention adherence: The participants' attendance at the weekly exercise sessions.

We considered progression feasible if each recruitment batch included at least four participants allocated to the intervention arm, trial retention exceeded 75%, and intervention attendance exceeded 50%.

### 2.6 Assessments

We assessed participants for change from baseline across a range of outcomes, including:

Physical fitness: International Physical Activity Questionnaire Short Form: A self‐report 7‐day recall questionnaire to assess total physical activity. It is the most widely used scale for this purpose though it appears to overestimate the person's level of activity (Lee et al. 2011).

6‐min walk test, 2‐min walk test and 20 m shuttle run: Measurements of aerobic capacity which have been validated in people with psychosis (Bernard et al. 2015; Vancampfort et al. 2019; de Oliveira Tavares et al. 2021).

Vertical jump height test and hand grip dynamometer: Well‐established proxy assessments for muscular strength and exercises in which the participants did not train. Good performance in both is associated with a range of health outcomes (Klavora 2000; Bohannon 2001).

Metabolic health: Blood Pressure, weight, height, body mass index, waist circumference, lipid profile, fasting glucose, HbA1c and CRP.

Mental health: The short form assessment of positive and negative symptoms (SANS and SAPS), used to assess positive and negative symptoms of psychosis (Dazzi and Shafer 2024).

Calgary Depression Scale for Schizophrenia (CDSS), a 9‐item, structured interview designed to assess depression severity in people with schizophrenia, separating it from psychotic or negative symptoms (Porter et al. 2022).

Social Anxiety Interaction Scale (SAIS), State Trait Anxiety Inventory (STAI) and Perceived Stress Scale (PSS): Self‐report scales designed to assess different types of anxiety/stress; namely social anxiety and transient (state)/long‐standing (trait) anxiety, respectively. Both have shown acceptable validity in people with psychosis (Aunjitsakul et al. 2021; Smith et al. 2021).

PSS: A scale measuring the degree to which situations in one's life are appraised as stressful. It is relied upon for its ease of use rather than its definitive psychometric properties (Lee 2012; Nielsen et al. 2016).

Quality of life and functioning: Euro QOL–5 Dimension (EQ5D5L) and Visual Analogue Scale (EQ‐VAS) assessing quality of life, and Global Assessment of Functioning (GAF) to assess functioning. Three easily and rapidly used scales with established psychometric validity (Feng et al. 2021; Cheng et al. 2021; Schwartz 2007).

Cognition: Cambridge Neuropsychological Test Automated Battery (CANTAB): A widely used cognitive battery which includes tests of verbal memory (Verbal Recognition Memory; assessing recall and recognition), psychomotor speed (Reaction Time; measuring simple and five‐choice response speed), executive function (One Touch Stockings of Cambridge; evaluating spatial planning), and social cognition (Emotion Recognition Task; assessing facial affect identification). It has been well established in psychosis research (Levaux et al. 2007).

The clinical research team measured blood pressure, BMI, waist circumference, physical activity and psychometric scale assessments at baseline, at the midpoint (4 weeks), and at the end of the eight‐week intervention. Gym staff at the Mardyke Arena performed fitness assessments at baseline, at the midpoint (4 weeks), and at the end of the eight‐week intervention. Adverse events were assessed at each supervised session and at endpoint assessments through participant self‐report and clinician enquiry.

After the third cycle (*n* = 24 participants), the midpoint (4‐week) assessment was no longer completed as patients perceived the assessment to be an onerous time commitment, which led to a high dropout rate. Thus, the remaining 16 participants had assessments at baseline and at the end of the eight‐week intervention only.

### 2.7 Randomisation and Blinding

We recruited participants for a maximum of one month for each cycle, until the number of people who would attend the exercise sessions reached capacity. We stratified participants by sex, with treatment allocation within each stratum determined by a 1:1 simple randomisation process using a manual envelope draw. Clinical staff (and thus outcome assessors) were aware of participants' allocation. Due to the nature of the intervention, it was not possible to blind participants to their group allocation. Each participant in the control group was offered the same intervention after the 8‐week study period had finished.

### 2.8 Sample Size Estimation

As a feasibility study, this sample size was not determined by a formal power calculation for efficacy, but was instead chosen to provide robust estimates of recruitment and retention rates, as well as to determine the variance of our primary and secondary outcomes (Thabane et al. 2010).

### 2.9 Data Analysis

#### 2.9.1 Data Preparation and Missing Data Analysis

We conducted all statistical analyses using R (v4.5.3) and the lme4 (Bates et al. 2015), tidyverse (Wickham et al. 2019), mice (van Buuren and Groothuis‐Oudshoorn 2011), and naniar packages (Tierney and Cook 2023). Prior to analysis, we inspected variables for distribution and outliers. We used logistic regression to investigate missing data patterns to determine the mechanism of missingness.

#### 2.9.2 Multiple Imputation

We employed Multiple Imputation by Chained Equations (MICE) as a sensitivity analysis to investigate the robustness of the exploratory findings. To prevent model instability due to the small sample size relative to the number of variables, we constructed a predictor matrix which restricted each variable's imputation model to a maximum of 8 predictors, which included forced key variables (Baseline score, Group, Age, Gender) and the highest correlated auxiliary variables. We generated 50 complete datasets using Predictive Mean Matching (PMM) to ensure imputed values remained within plausible observed ranges.

#### 2.9.3 Outcome Analysis

We analysed outcomes using an Analysis of Covariance (ANCOVA). The models predicted the Endpoint value adjusting for the Baseline value and Group allocation (Endpoint ~ Baseline + Group). This method estimates the “Adjusted Difference” between groups at the endpoint, conditional on their starting levels, which provides greater statistical power than analysing change scores alone. These analyses were exploratory and intended to estimate effect sizes and variability for future trials rather than to test definitive hypotheses. Our primary analysis used Intention‐to‐Treat, and we conducted a Per‐Protocol analysis as a sensitivity analysis, where Per‐Protocol was defined as attending at least 50% of the exercise sessions.

For the multiple imputation analysis, we ensured robustness against double missingness (participants with no data at either timepoint); we restricted the analysis to a modified intention‐to‐treat (mITT) population, defined as all randomised participants who provided a valid baseline measurement for the specific outcome being analysed. We excluded participants missing both baseline and endpoint data for a given variable from the analysis of that specific variable. We pooled results from the 50 datasets using Rubin's Rules to generate valid standard errors, confidence intervals and *p*‐values that account for the uncertainty introduced by the missing data. We set statistical significance at alpha = 0.05.

## 3 Results

**FIGURE 1:**
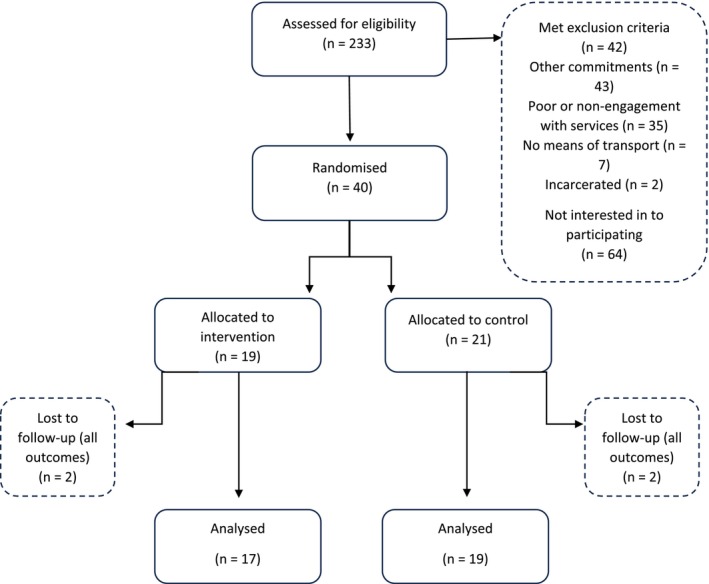
Study flowchart.

Forty individuals with a FEP participated in the study, 19 in the intervention group and 21 in the control group. Figure 1 displays the flow of recruited and analysed participants and Table 1 displays baseline data of all participants. Participants were mostly male (*n* = 24, 60%) and had an average age of 34.6 years (SD 11.6 years). At baseline, the 40 participants exhibited a high prevalence of cardiometabolic risk factors: 23 of 38 had an elevated BMI (≥ 25 kg/m^2^), 24 of 35 presented with elevated cholesterol (≥ 5.0 mmol/L), and 19 of 38 met the gender‐specific criteria for increased waist circumference, while 6 of 35 had HbA1c levels within the pre‐diabetic or diabetic range (≥ 42 mmol/mol). There were no differences in the characteristics of the people assigned to the control group compared to those assigned to the intervention group.

**TABLE 1:** Baseline characteristics of participants. Test statistics used: Wilcoxon rank sum test for continuous; Pearson's Chi‐squared test for categorical data.

Characteristic	*N*	Overall *N* = 40 mean (SD)	Control *N* = 21 mean (SD)	Intervention *N* = 19 mean (SD)	*p*
Demographics					
Age (years)	40	34.6 (11.6)	35.3 (12.9)	33.7 (10.4)	0.745
Gender	40				0.366
Female		16 (40%)	7 (33%)	9 (47%)	
Male		24 (60%)	14 (67%)	10 (53%)	
Anthropometrics					
Weight (kg)	38	76.4 (13.6)	76.0 (12.9)	76.8 (14.5)	> 0.999
Body mass index (kg/m^2^)	38	26.8 (4.5)	26.6 (4.2)	27.0 (4.8)	0.930
Waist circumference (cm)	38	92.4 (13.5)	93.3 (13.7)	91.5 (13.7)	0.549
Hip circumference (cm)	38	102.5 (8.3)	103.2 (8.5)	101.8 (8.3)	0.501
Systolic blood pressure (mmHg)	30	112.9 (11.6)	113.8 (12.3)	112.2 (11.3)	0.600
Physical fitness					
Mean hand grip strength (kg)	23	32.9 (9.2)	29.9 (8.2)	35.2 (9.6)	0.277
6‐min walk distance (m)	32	515.6 (94.6)	537.3 (69.6)	496.5 (110.7)	0.413
20 m shuttle run (level)	30	4.5 (1.9)	4.3 (2.1)	4.8 (1.7)	0.492
Psychometrics					
Anxiety (STAI Total)	37	78.4 (35.1)	67.2 (36.2)	89.1 (31.3)	0.080
Global functioning (GAF)	32	61.4 (10.5)	60.9 (11.2)	61.8 (10.3)	> 0.999
Depression (CDSS)	35	6.6 (6.1)	5.8 (6.2)	7.3 (6.1)	0.407
SANS/SAPS combined score	33	7.2 (6.0)	8.1 (6.6)	6.3 (5.5)	0.480
Quality of life (EQ‐5D Index)	35	0.7 (0.3)	0.8 (0.2)	0.7 (0.4)	0.128
Cognitive (CANTAB)					
Motor screening: mean latency (ms)	31	959.6 (341.0)	1002.7 (414.6)	913.6 (246.1)	0.599
Verbal memory: free recall (total)	31	5.0 (2.1)	5.1 (2.5)	5.0 (1.8)	0.994
Verbal memory: immediate recognition	31	30.8 (4.5)	30.5 (3.8)	31.0 (5.2)	0.425
Verbal memory: delayed recognition	30	30.3 (4.3)	30.6 (4.3)	30.1 (4.4)	0.601
Reaction time: simple RT (ms)	20	360.7 (48.4)	361.7 (40.5)	359.9 (56.0)	0.824
Reaction time: 5‐choice RT (ms)	20	419.6 (59.8)	410.5 (38.9)	427.1 (73.7)	0.766
Executive function: OTS problems solved	20	9.1 (3.3)	9.2 (1.8)	8.9 (4.2)	0.878
Executive function: OTS latency (s)	20	17.5 (11.5)	18.9 (14.8)	16.4 (8.5)	0.882
Emotion recognition: total hits	15	26.9 (8.3)	31.0 (6.5)	24.1 (8.5)	0.098
Biomarkers					
Total cholesterol (mmol/L)	35	5.5 (1.1)	5.5 (1.0)	5.5 (1.2)	0.895
HbA1c (mmol/mol)	35	36.3 (4.4)	36.7 (4.5)	35.9 (4.4)	0.691
C‐reactive protein (mg/L)	33	2.7 (2.8)	2.5 (2.0)	3.1 (3.5)	0.732

### 3.1 Feasibility Metrics

**FIGURE 2:**
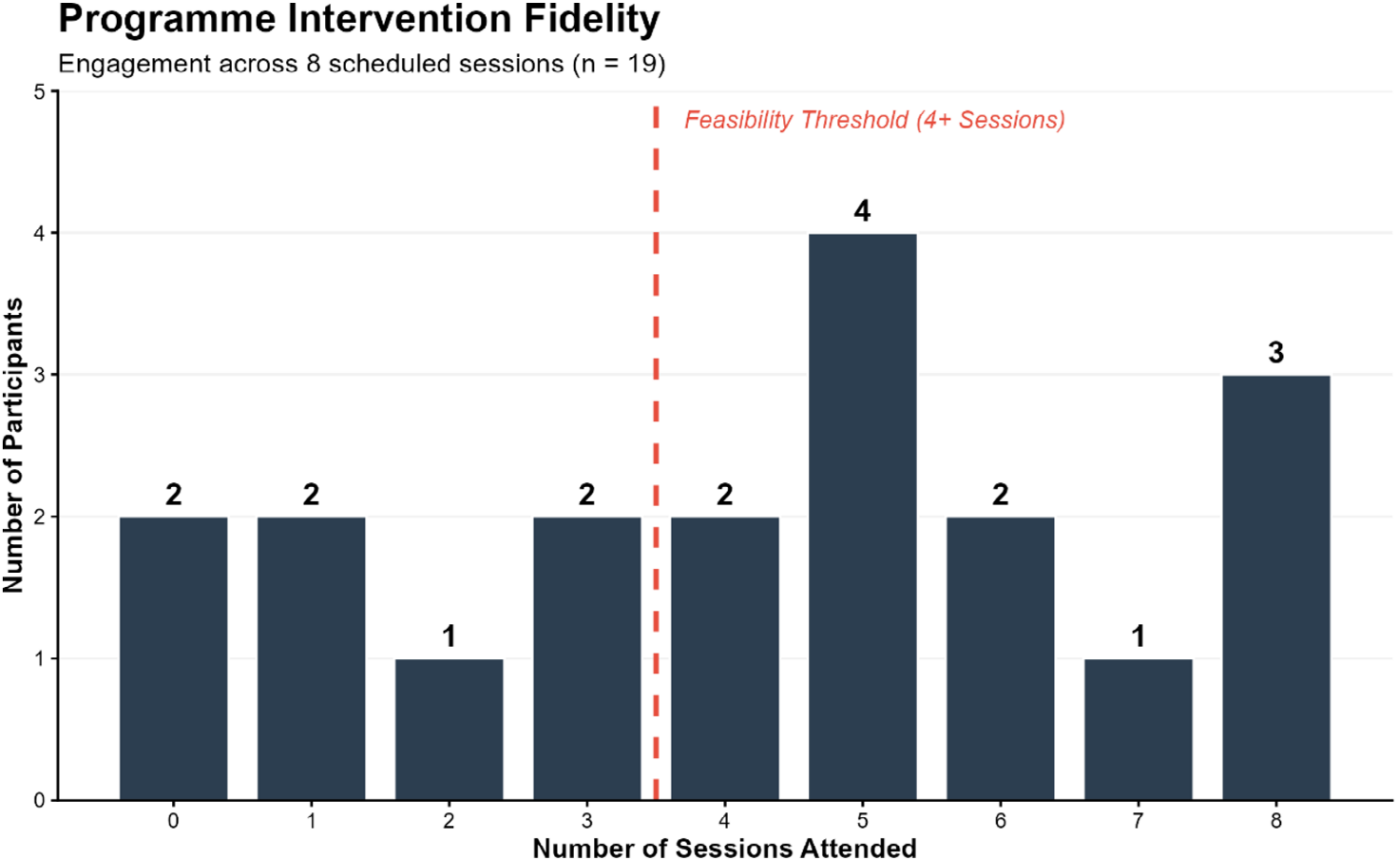
Histogram of the percentage of sessions attended by participants in the intervention group.

Of the 19 people recruited to the intervention arm, 7 (36.8%) attended less than half of the 8 sessions, and 2 (10.6%) did not attend any of the sessions. The total percentage of individual‐sessions attended was 53.3%. Figure 2 displays the frequency at which participants in the intervention group attended the scheduled sessions.

The study encountered significant attrition at the follow‐up stage, leading to low completion rates across all assessment domains. While only four participants were lost to follow‐up, participants often completed only a subset of the assessments at both baseline and after the intervention. Complete case data for physical health outcomes ranged from only 11 participants (27.5%) to 28 participants (70%). Fitness metrics had low completion rates, with between 11 and 13 participants completing both baseline and endpoint assessments (27.5% and 32.5%, respectively). The CANTAB assessments also suffered from significant attrition: 16 participants (40%) underwent both baseline and endpoint assessments, and just five completing the Emotion Recognition Test (the final part of the battery). The logistic regression analysis for attrition showed that no variable systematically predicted missingness. A matrix displaying the predictors of attrition can be found in the Supporting Information results.

### 3.2 Outcomes

**FIGURE 3:**
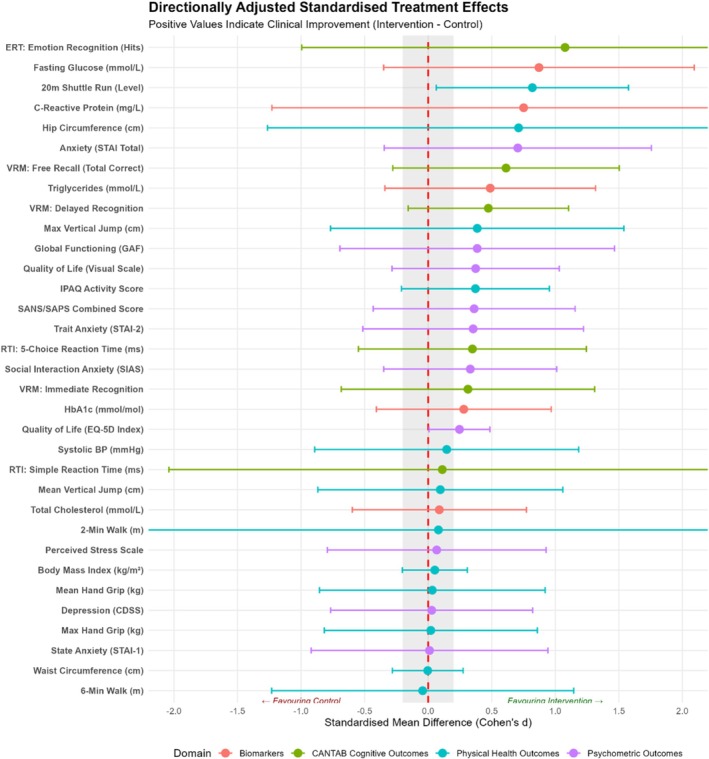
Visual summary of the effect of the intervention.

Table 2 displays a numerical summary of the effect of the intervention on selected variables among complete cases, while Figure 3 displays a visual summary of all variables Statistically significant improvements in the 20 m shuttle run (mean difference 1.52 levels [95% CI: 0.12 to 2.93], *p* = 0.036) and EQ‐5D‐5L (mean difference 0.08 [95% CI: 0.01, 0.16], *p* = 0.031) were imprecise and sensitive to missing data assumptions. Although the mean IPAQ score for the intervention group doubled from 2346 to 4492, this had a large standard error and was due to one outlier in the intervention group. When this outlier was removed, the mean score fell to 3234 (SD 2574). No consistent pattern of benefit or harm was observed across outcomes. The Per‐Protocol analysis had similar findings, and can be seen in Supporting Information results.

**TABLE 2:** Outcomes before and after the intervention in the control and intervention groups.

Outcome measure		Baseline	Endpoint		Baseline	Endpoint		
	Control group			Intervention group			Analysis	
	*N*	Mean (SD)	Mean (SD)	*N*	Mean (SD)	Mean (SD)	Adj. diff [95% CI]	*p*
Physical health outcomes								
Body mass index (kg/m^2^)	12	28.3 (3.5)	28.5 (4.0)	14	27.3 (5.1)	27.4 (5.1)	−0.13 [−1.13, 0.86]	0.783
Systolic BP (mmHg)	7	120.7 (7.0)	114.1 (8.4)	11	113.0 (12.5)	110.5 (9.7)	−1.81 [−11.92, 8.29]	0.708
6 min walk (m)	7	554.3 (51.3)	557.1 (53.1)	5	568.0 (59.3)	562.0 (57.6)	−2.40 [−68.27, 63.46]	0.936
20 m shuttle run (level)	7	4.4 (2.2)	4.1 (2.5)	6	4.6 (1.4)	5.8 (2.3)	1.52 [0.12, 2.93]	0.036
Max vertical jump (cm)	6	25.7 (7.7)	25.2 (9.2)	5	33.6 (8.2)	32.8 (2.6)	3.07 [−6.10, 12.24]	0.463
Psychometric outcomes								
Social interaction anxiety	9	21.7 (15.8)	21.8 (16.7)	16	38.9 (22.9)	26.5 (21.0)	−8.34 [−19.65, 2.97]	0.140
Depression (CDSS)	11	6.2 (5.8)	6.2 (6.9)	15	7.3 (6.3)	6.6 (4.8)	−0.11 [−4.37, 4.14]	0.957
SANS/SAPS combined	11	7.8 (6.3)	7.1 (6.1)	12	6.8 (5.6)	4.6 (3.6)	−2.25 [−6.43, 1.93]	0.274
EQ‐5D‐5L index	10	0.8 (0.2)	0.8 (0.2)	13	0.7 (0.4)	0.8 (0.3)	0.08 [0.01, 0.16]	0.031
Global functioning (GAF)	10	62.1 (11.1)	60.6 (13.1)	12	56.8 (6.8)	60.8 (13.3)	5.62 [−3.51, 14.74]	0.213
CANTAB cognitive outcomes								
VRM: free recall (correct)	6	6.3 (2.5)	5.3 (1.4)	10	4.8 (1.5)	5.0 (2.4)	−0.50 [−3.10, 2.11]	0.687
VRM: immediate recog (correct)	6	33.2 (2.6)	30.8 (3.8)	10	31.2 (5.5)	28.4 (5.0)	−1.13 [−5.16, 2.89]	0.553
VRM: delayed recog (correct)	6	31.0 (6.2)	31.2 (3.4)	8	31.2 (3.4)	28.9 (2.4)	−2.38 [−5.24, 0.48]	0.095
RTI: simple reaction time (ms)	5	351.6 (36.3)	427.5 (125.2)	5	358.1 (81.2)	415.0 (86.1)	−19.64 [−151.53, 112.26]	0.735
RTI: 5‐choice reaction time (ms)	5	425.0 (45.5)	462.7 (92.9)	5	434.5 (102.1)	447.7 (96.7)	−25.18 [−92.93, 42.58]	0.409
OTS: problems solved (1st Choice)	5	10.0 (1.9)	9.2 (1.9)	4	8.2 (4.0)	11.2 (2.8)	2.96 [−0.23, 6.15]	0.063
OTS: mean latency (ms)	5	23089.0 (19445.0)	17839.5 (13744.8)	4	16200.2 (10530.8)	15608.3 (7776.1)	2301.93 [−5794.67, 10398.53]	0.513
ERT: Emotion Recog (Total Hits)	3	27.7 (5.9)	30.3 (2.3)	2	26.0 (1.4)	25.5 (0.7)	−4.59 [−13.42, 4.24]	0.155

*Note:* Data presented as Mean (SD). Difference = Intervention minus Control (adjusted for baseline value). *N* reflects participants with valid data at both timepoints (Complete Case).

Participants in the intervention group did not suffer any harms related to the exercise programme. No participants suffered a relapse of psychosis during the study timeframe.

### 3.3 Imputed Data

To verify the complete cases results, we conducted a sensitivity analysis using multiple imputation, whose full results can be seen in the Supporting Information results. The complete case estimates generally matched the imputed models in direction and size, but the significance pattern changed for the 20 m shuttle run. Unlike the primary imputed model, the complete case data showed a significant difference in the shuttle run and the EQ5D5L. Psychometric outcomes remained almost identical across both methods.

## 4 Discussion

### 4.1 Summary of Findings

This pragmatic, service‐coordinated randomised controlled trial demonstrates the challenges of implementing an exercise intervention in an early intervention for psychosis service and provides insight for future research in the area. High rates of attrition in completing research assessments led to small sample sizes for assessments of outcomes. Additionally, the intervention was somewhat poorly attended, despite efforts by staff to encourage attendance. This study demonstrates the challenges of implementing and evaluating an exercise intervention in a real‐world FEP service. While exploratory analyses showed a directionally favourable trend for the intervention, the study was not powered for efficacy, and findings were highly sensitive to missing data.

### 4.2 Interpretation of Results and Implications for Implementation and Future Research

The primary contribution of this study is methodological and implementation‐focused, rather than providing evidence of clinical efficacy. These findings highlight the need for adapted trial designs when evaluating lifestyle interventions in early psychosis services. Our study faced challenges in participant retention for both the exercise intervention and at follow‐up outcome assessments. Despite the sessions being just once per week, over a third of participants attended less than half the offered exercise sessions. Such low attendance has been reported in a study using a similar group‐exercise intervention to our own (Midtgaard et al. 2021), and even in studies offering online exercise sessions (French et al. 2026). Meanwhile studies using a participant‐selected exercise have reported higher rates of attendance (Fisher et al. 2020; Dunleavy et al. 2024). The average dropout rate in RCTs assessing exercise in psychosis is just over 25% (Vancampfort et al. 2016), while the average attendance has not been systematically investigated. Low attendance at exercise interventions may plausibly be ascribed to the motivational challenges faced by people with psychosis (Arnautovska et al. 2022; Firth, Rosenbaum, Stubbs, Gorczynski, et al. 2016), and attendance rates are higher at non‐exercise‐based group interventions (Sedgwick et al. 2021). Contingency management strategies appear to improve attendance rates, although this may spark ethical concerns regarding coercion (Sedgwick et al. 2021). Our study also faced challenges in recruitment, with low rates of enrolment compared to the overall caseload across the two clinics. This is a problem evident in other studies of exercise in FEP (Midtgaard et al. 2021; Dunleavy et al. 2024), with studies reporting higher recruitment rates seeming to be selecting from a pre‐screened cohort (French et al. 2026; Firth et al. 2018). This is evidently problematic, as the low rates of attendance in studies appear to come from the most motivated of the FEP cohort. This may raise questions about the scalability and generalisability of exercise interventions in FEP services.

While gym‐based cardio and weight‐training may be the most acceptable forms of exercise, this is not universal (Firth, Rosenbaum, Stubbs, Vancampfort, et al. 2016). Additionally, identifying and utilising the patient's motivation to exercise would likely increase attendance (Firth, Rosenbaum, Stubbs, Gorczynski, et al. 2016). Thus, future research and clinical‐level exercise programmes should avoid a one‐size‐fits‐all approach to exercise prescription in FEP (Stubbs et al. 2026). While a service‐embedded exercise physiologist would be the gold standard solution, this not an option in most services. In the absence of a dedicated exercise professional, other clinical staff should do as much as possible to individualise exercise advice and prescription for patients. Simple steps could include enquiring about the person's interests, available time for exercise and the goals they have, for example, improve cardiovascular fitness, increase strength, enhance longevity, or purely aesthetic benefits. This would allow for personalised prescription (which could be provided in a written format as it would be for medications), outlining the frequency, intensity, time and type (FITT) (Bricca et al. 2026). Types of exercise would be guided by patient preference and could consider method (e.g., resistance training vs. running), social context (e.g., group vs. solo), and competitive nature (e.g., sport vs. non‐competitive). Regional differences in availability of groups and modalities would further guide prescription, for example walking groups for people with low cardiorespiratory capacity or sports clubs for people who would prefer this type of exercise. This should ideally be performed using motivational interviewing principles, with as much assistance given as is required by the individual (Stubbs et al. 2026).

The high rates of attrition for outcome assessments led us to no longer collect midpoint data after three cycles. While we aimed for a broad coverage of outcomes as part of an exploratory study, the high rates of attrition reflected an infeasible assessment battery. This was particularly evident in the rates of completion of the CANTAB subtests. The CANTAB itself faced difficulties in engagement, with 16 participants (40%) completing the first test (Free Recall) at both baseline and endpoint assessments. By the time the CANTAB reached the last test (Emotion Recognition), completion at both baseline and endpoint dropped to just five people (12.5%). Each additional outcome adds to the likelihood that the participant tires of the assessment and skips outcome measures (Jeong et al. 2023). This is likely to be accentuated in trials including people with psychosis, whose cognitive and negative symptoms would make long assessments more tiring still (Arnautovska et al. 2022; McCutcheon et al. 2023). Future research should be mindful of the time and cognitive burden of assessments for people with psychosis and be judicious in the amount and duration of assessments.

### 4.3 Clinical Implications

No definitive conclusions regarding clinical effectiveness can be drawn. While some exploratory estimates were directionally favourable, these were imprecise and sensitive to missing data assumptions. The findings primarily support the rationale for further adequately powered and methodologically streamlined trials.

People with psychosis have been persistently shown to have poor cardiorespiratory fitness, a problem that worsens in step with the progression of their psychotic illness (Heggelund et al. 2020). Given the importance of cardiorespiratory fitness in preventing morbidity and mortality (Kodama et al. 2009), it is essential that this problem is addressed at an early stage. Failure to do so may lead to a deterioration in fitness which becomes increasingly difficult to reverse (Bouchard and Rankinen 2001). While exercise may lack the magnitude of effect of pharmacological interventions in specific domains, it has a global positive impact, lack of adverse effects, and is more likely to engender the autonomy, self‐actualisation and self‐esteem which are crucial in overcoming the stigma associated with psychotic disorders (Firth et al. 2019). Exercise might be particularly important in promoting recovery in younger people, by integrating their notion of being a young person with being a patient with a psychiatric diagnosis (Nielsen et al. 2016). Any exercise intervention should be delivered as part of a multi‐pronged approach to improving physical health, while ensuring continuity and long‐term physical health monitoring (Nielsen et al. 2026).

**Box 1: Implications for Design of Trials Assessing Exercise in First Episode Psychosis**

- *Prioritise core outcomes*: In an attempt to investigate a broad range of outcomes, our study was likely overinclusive of outcome measures. Future trials should focus on circumscribed research questions; both to avoid the problem of multiple testing and to maximise completion of the most important outcome of the study.
- *Reduce assessment burden*: While our study achieved above‐average levels of retention, many of the outcomes were not fully completed. This was likely due to our assessments being overly time consuming, leading to fatigue and aversion to the assessment. Future trials should be conscious of the time and cognitive burden of assessments. This is particularly true in FEP cohort who face cognitive and motivational difficulties.
- *Individualise intervention*: While our exercise intervention allowed for some flexibility in intensity, all participants followed a set programme of exercises. Trials which have allowed for individualisation of exercise prescription have achieved higher rates of attendance. This literature suggests that allowing the participant some input into the type (e.g., cardiovascular vs. resistance training) or subtype (e.g., machine weights vs. free weights) would likely increase enjoyment and attendance.
- *Consider hybrid effectiveness‐implementation designs*: Our study was performed in a clinical setting, with healthcare staff performing outcomes and attempting to maximise engagement and attendance. This presents challenges not captured by interventions delivered in a research environment. Future research using hybrid effectiveness‐implementation designs could provide clarity to clinical staff and managers on how to choose both the most effective interventions and how to best implement those interventions within their service.
- *Qualitative research*: Our study experienced high attrition rates within assessments, that is, participants attended outcome assessments but experienced difficulty in completing all aspects of the assessment. This was particularly evident in the fitness‐related assessments. Future qualitative research assessing participants' experience of burdensome outcome assessments could clarify solutions to this problem, and highlight how to optimise assessments for both researchers and participants.

### 4.4 Study Limitations

The main limitation of the study is the small sample size, mostly due to a high dropout rate. The small sample size of this trial led to some directional imbalances at baseline. Although we adjusted for these differences statistically, the potential for regression to the mean or floor effects in the control group cannot be entirely ruled out and should be considered when interpreting the magnitude of any effects. The high dropout rate could also lead to attrition bias. Our analysis using imputed data suggests this attrition bias may have led to the sole statistically significant findings of the study, that is, that participants who improved least may have been most likely to drop out. This is on top of the likely selection bias, which is evident by the low rate of enrolment in our study from the total pool of patients in the two services. The high rate of attrition was likely due to the long time needed (over 2 h) to complete the full battery of assessments and should guide future research. This assessment battery was also unblinded, which carries particular importance for clinician‐rated scales, that is, GAF, SANS/SAPS, and CDSS. Our intervention itself was not universally attended, which likely attenuated any effects that may have occurred for a fully attended exercise programme. Additionally, our exercise intervention was relatively short in duration, which likely restricted the magnitude of any effects. We also did not record heart rate or rate of perceived exertion, which could have provided subjective and objective intensity of the exercise. Lastly, we did not correct for false‐discovery rates, and the study should be interpreted as exploratory and hypothesis‐generating.

## 5 Conclusion

Overall, the findings indicate that future research in this area should take steps to maximise retention at exercise sessions and outcome assessments. Such steps may include limiting barriers in time and effort for participants to complete outcome data and by individualising exercise prescription. The absence of change in metabolic outcomes should be interpreted as neutral rather than negative in the context of an 8‐week pilot study with variable attendance and completion rates. This lack of consistent improvement across outcome domains could be both an indicator of an underpowered study or a small effect size of the intervention.

## Funding

B.W.O'M is supported by the Health Research Board (ICAT‐2022‐001) and the ICAT Programme, which is supported by the Health Service Executive, National Doctors Training and Planning, the Health and Social Care, Research and Development Division, the Northern Ireland Medical and Dental Training Agency, the Department of Agriculture, Food and the Marine and the College of Anaesthesiologists of Ireland.

## Conflicts of Interest

G.C. received honoraria from Janssen, Heel Pharmaceuticals, Probi, Boehringer Ingelheim and Apsen as an invited speaker; and research funding from Pharmavite, Reckitt, Tate and Lyle, Nestle and Fonterra. G.C. is or has been a paid consultant for Heel Pharmaceuticals, Bayer Healthcare, Yakult and Zentiva. All other authors declare no competing interests.

## Supporting information


**Figure X.** Trace plots for imputed data.

**Figure Y.** Distribution plots for imputed data.

**Table S1:** Results of the Intention‐to‐treat analysis with imputed data.

## Data Availability

The data that support the findings of this study are available on request from the corresponding author. The data are not publicly available due to privacy or ethical restrictions.
