# Supplementary figures and images for: A Randomised Feasibility Trial of a Service‐Coordinated Exercise Intervention in First‐Episode Psychosis: Challenges in Implementation and Outcome Assessment
Source: Early Interv Psychiatry. 2026 Jul 9;20(7):e70193. doi: 10.1111/eip.70193 (PMC13349341; doi:10.1111/eip.70193)

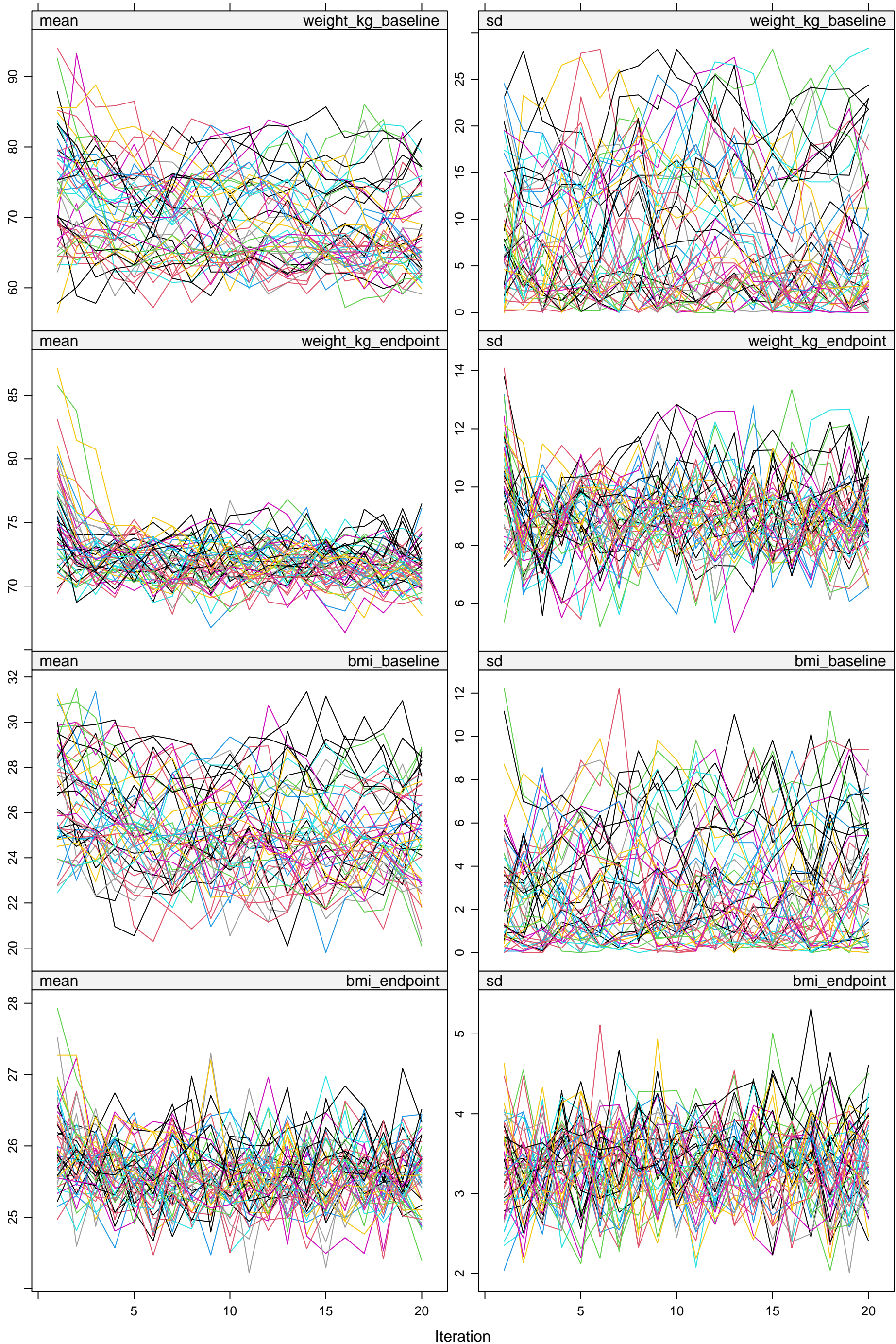

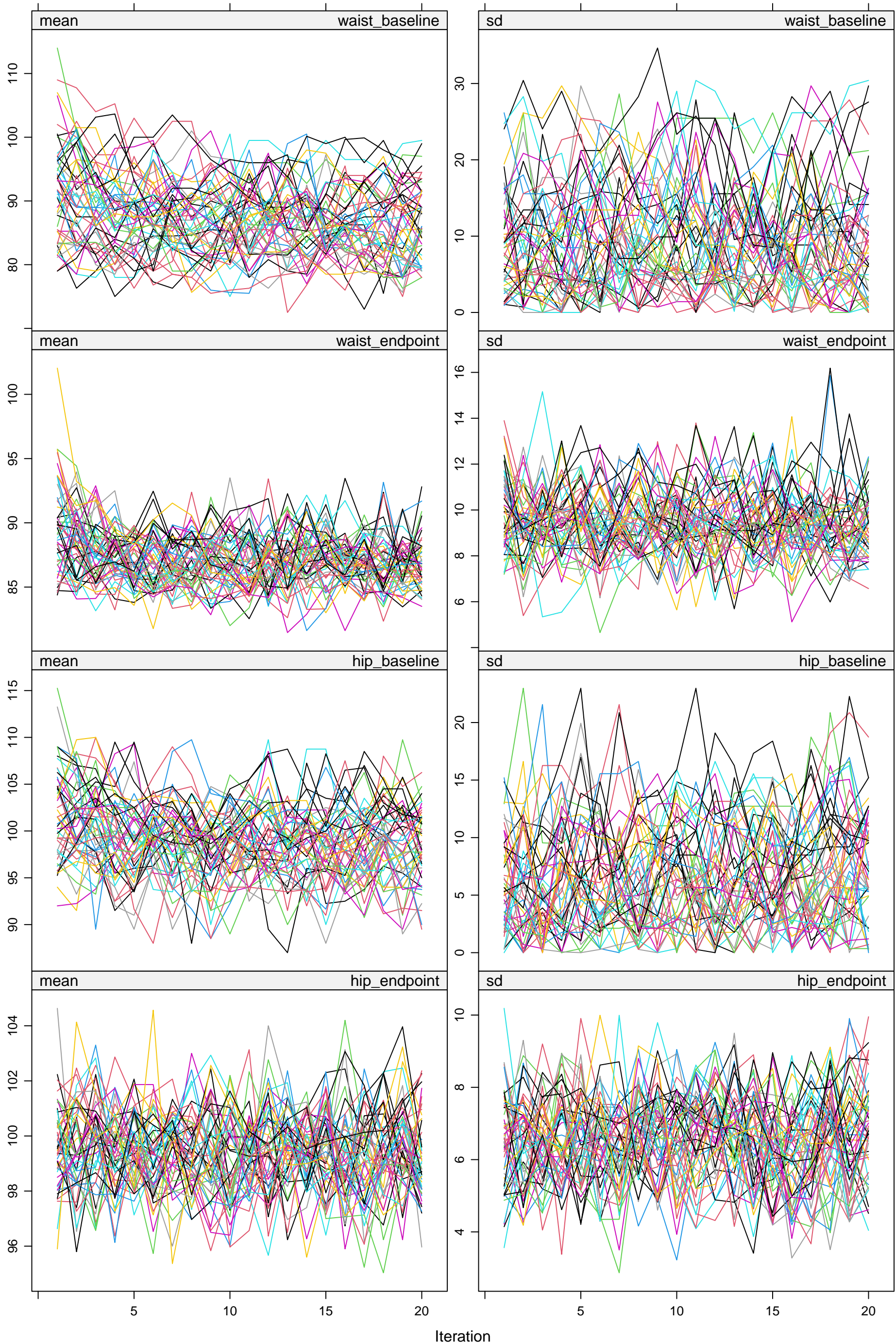

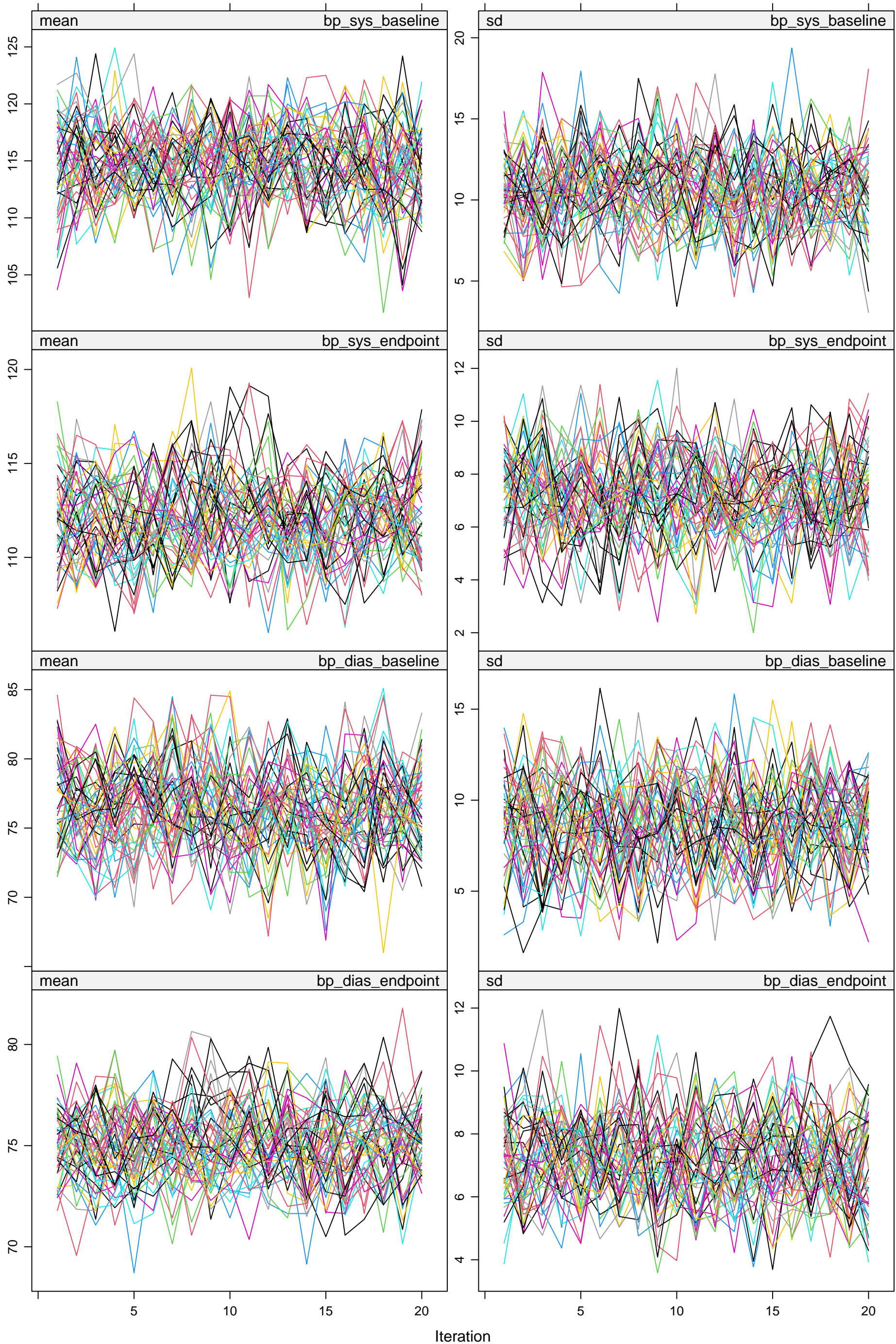

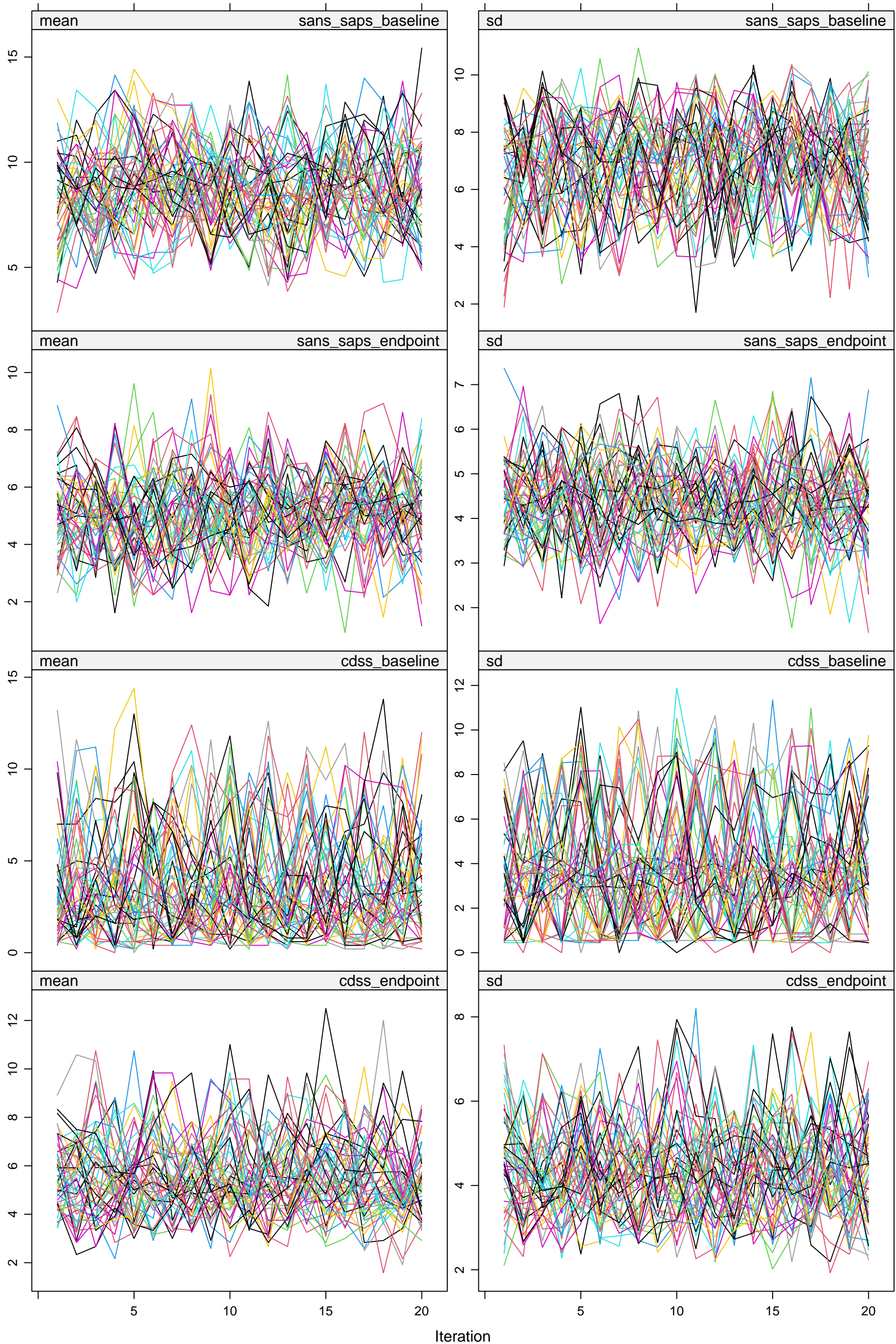

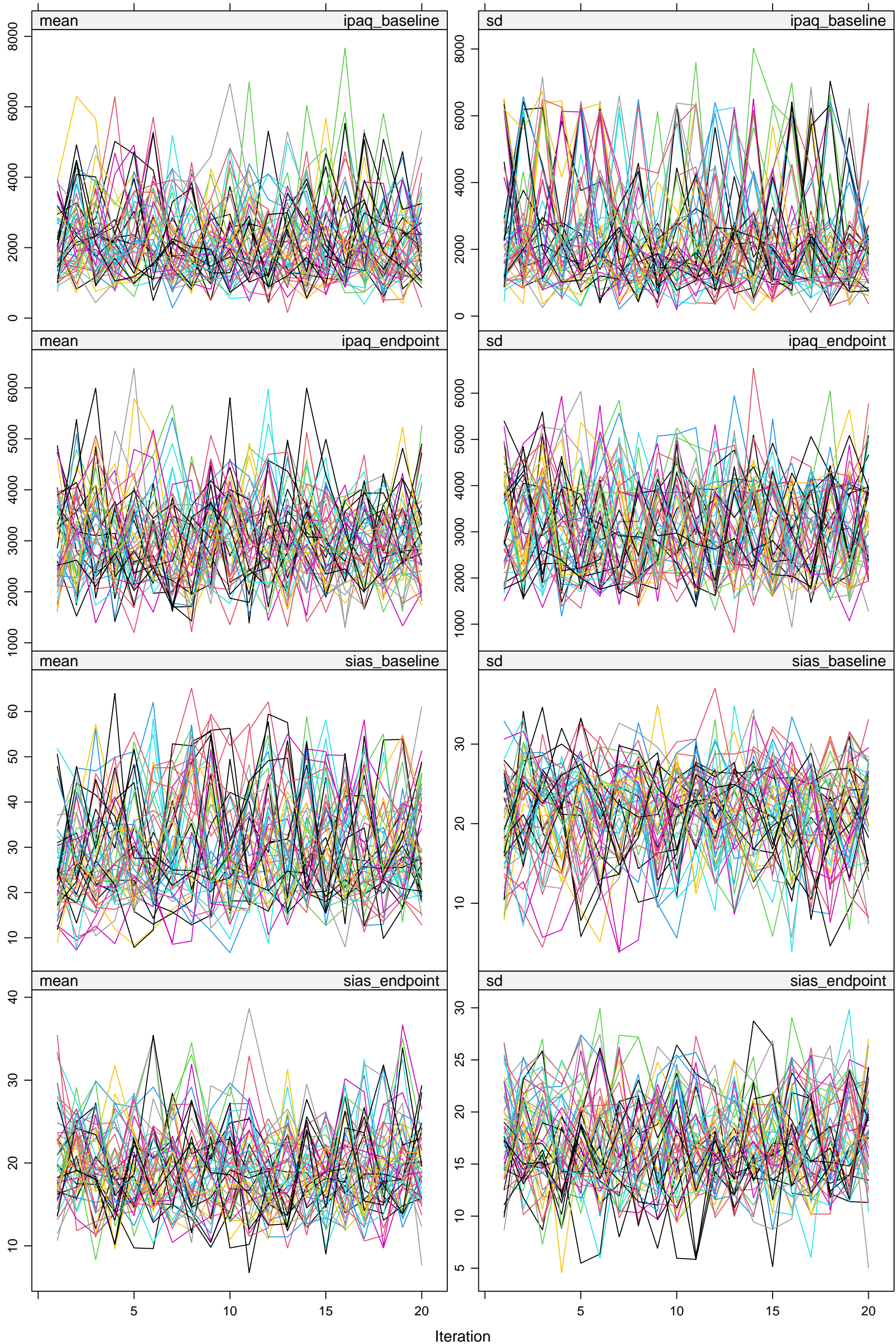

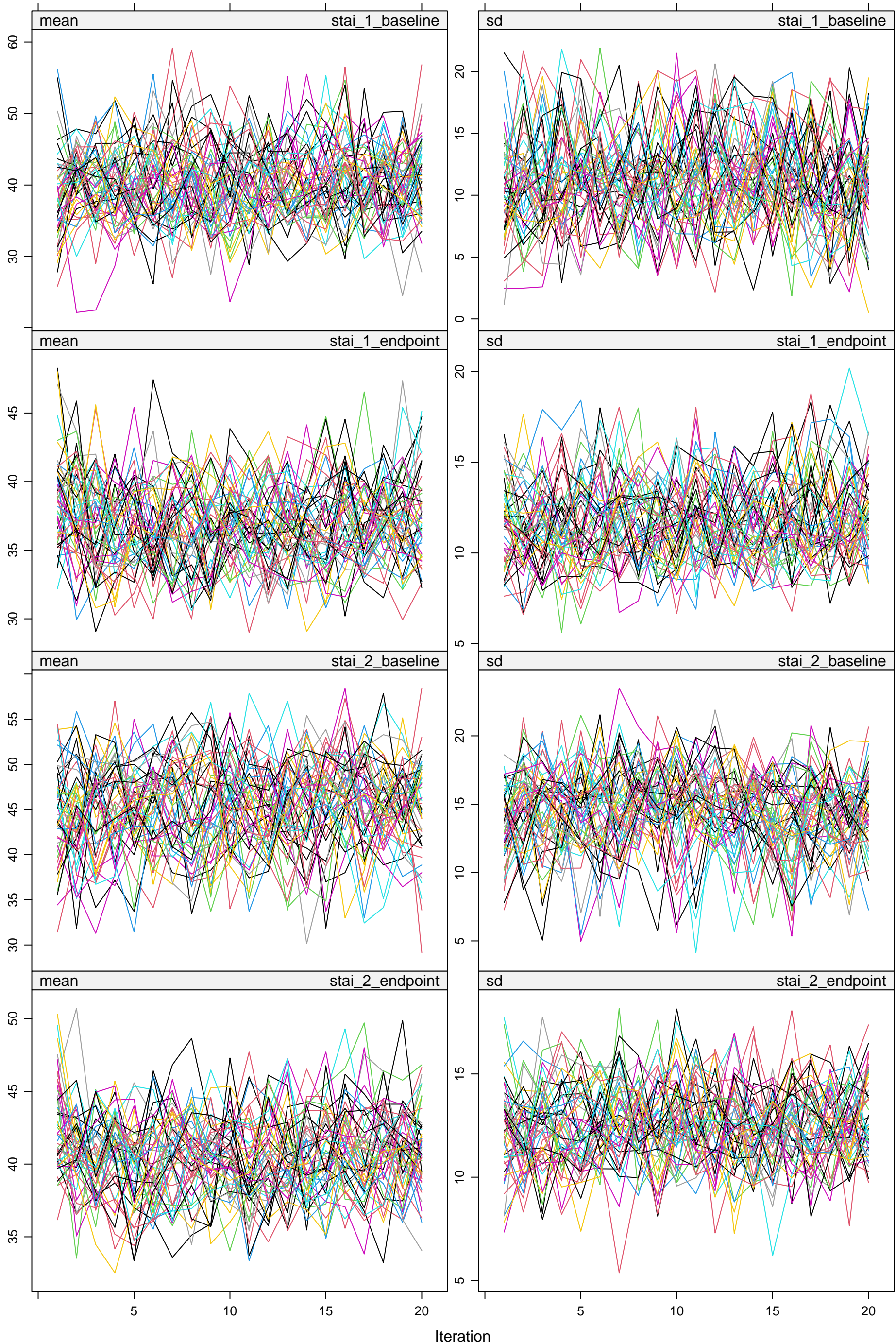

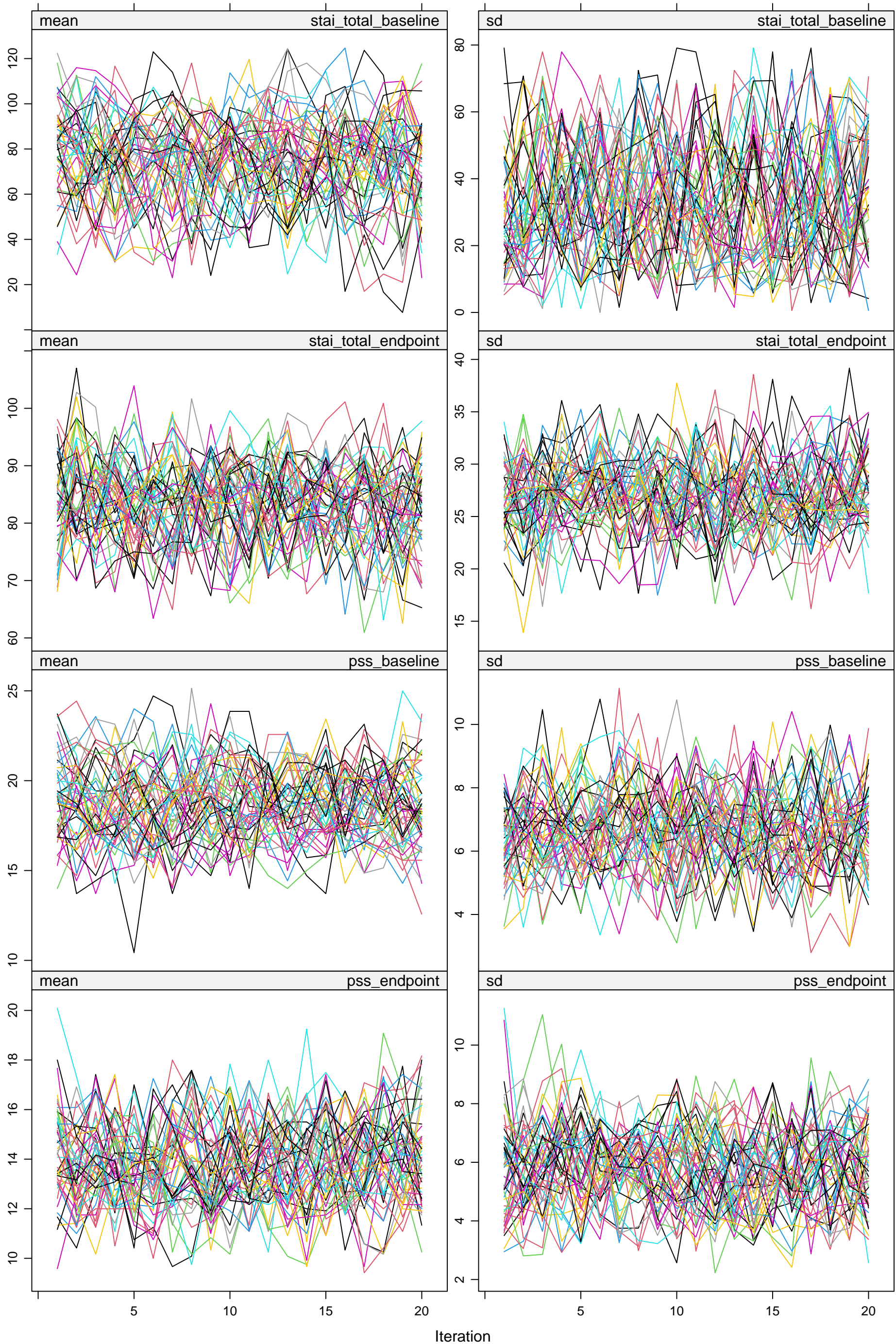

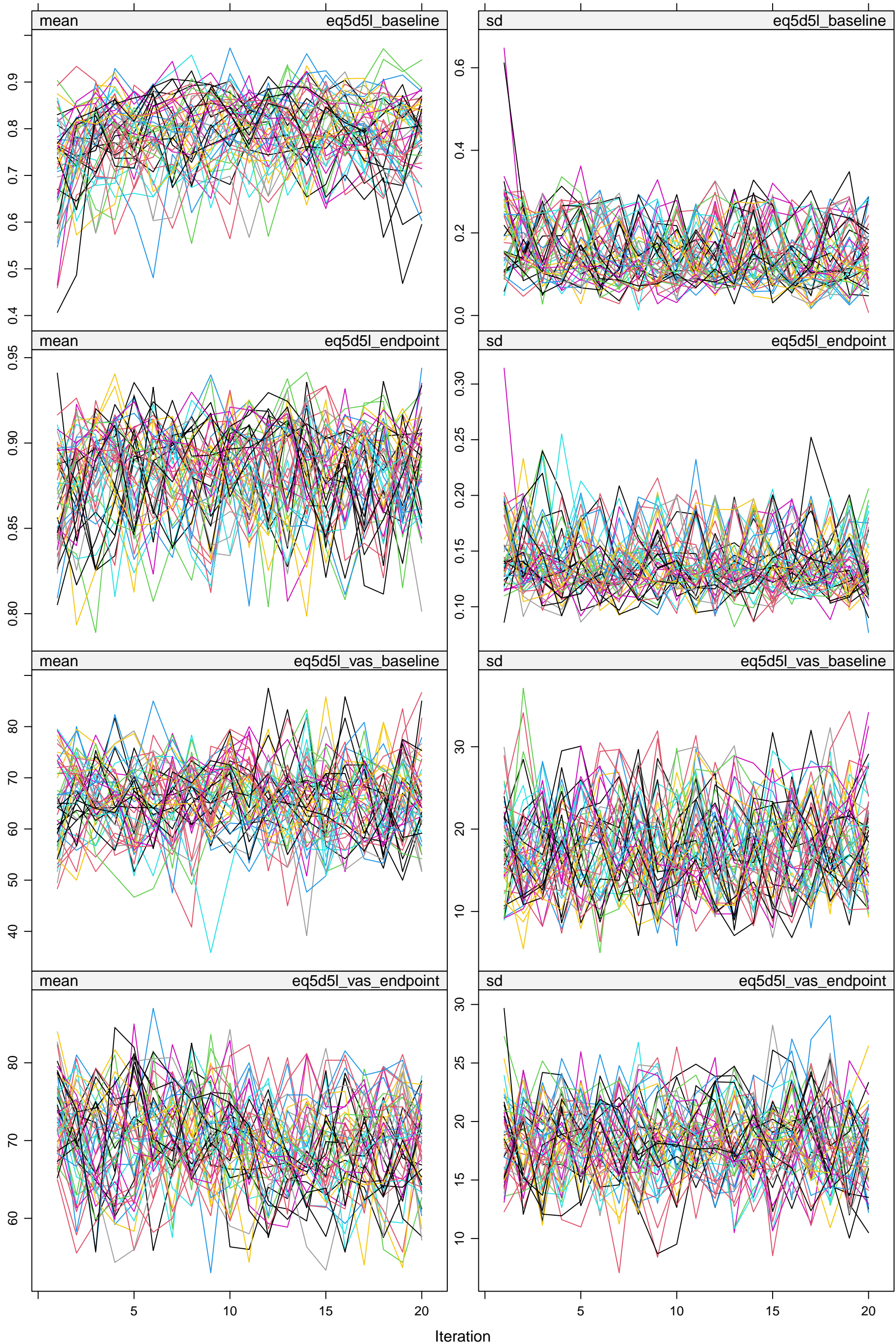

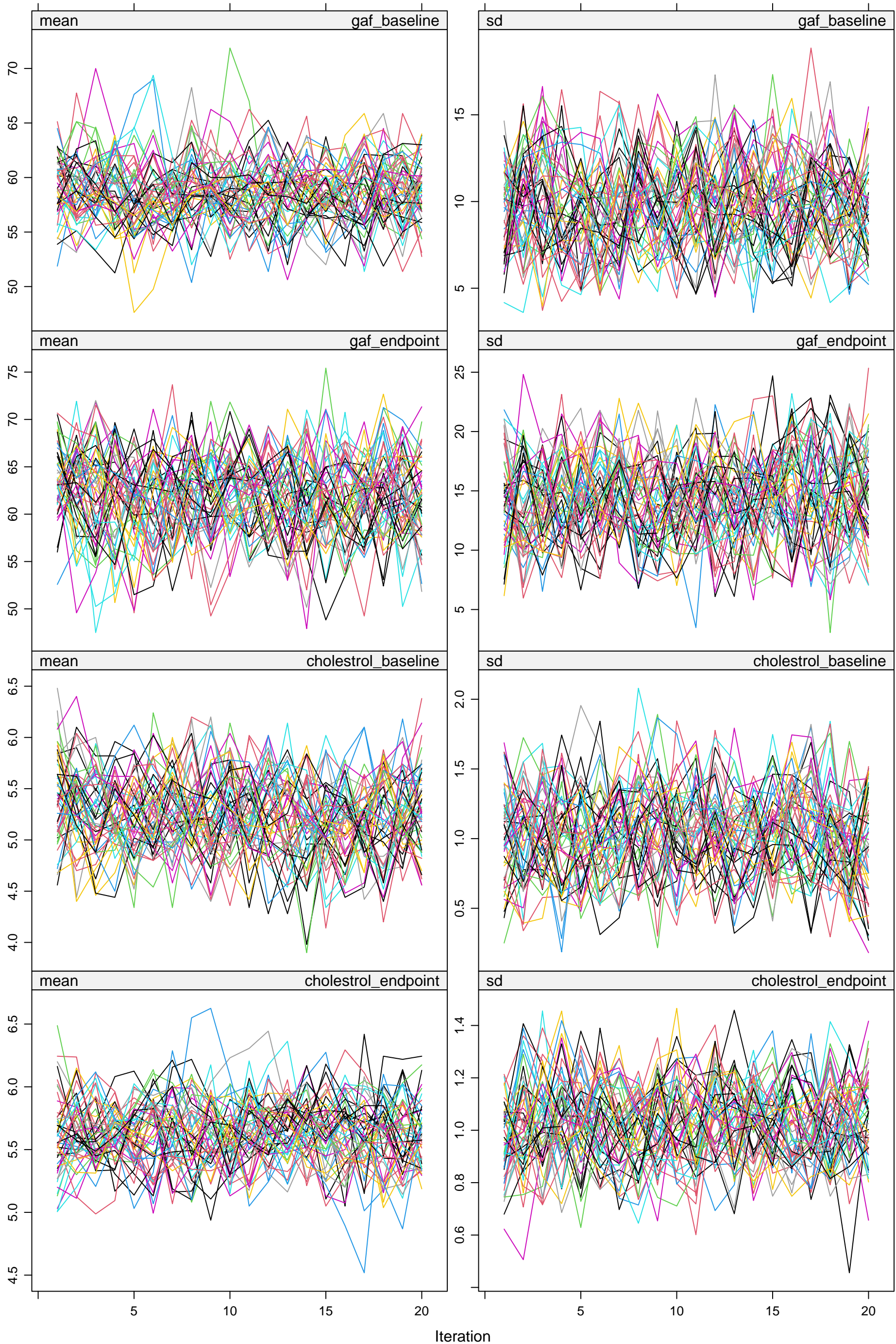

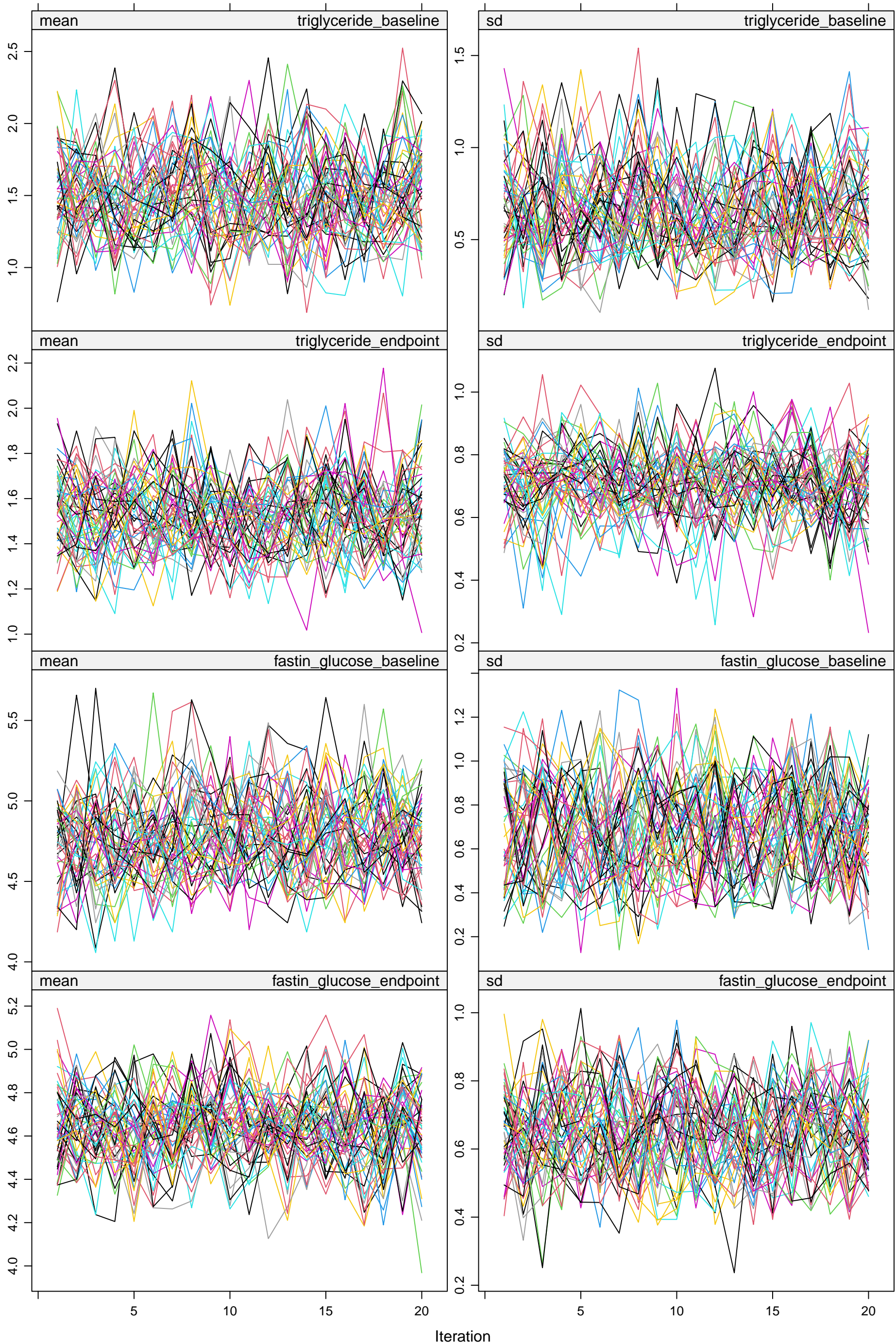

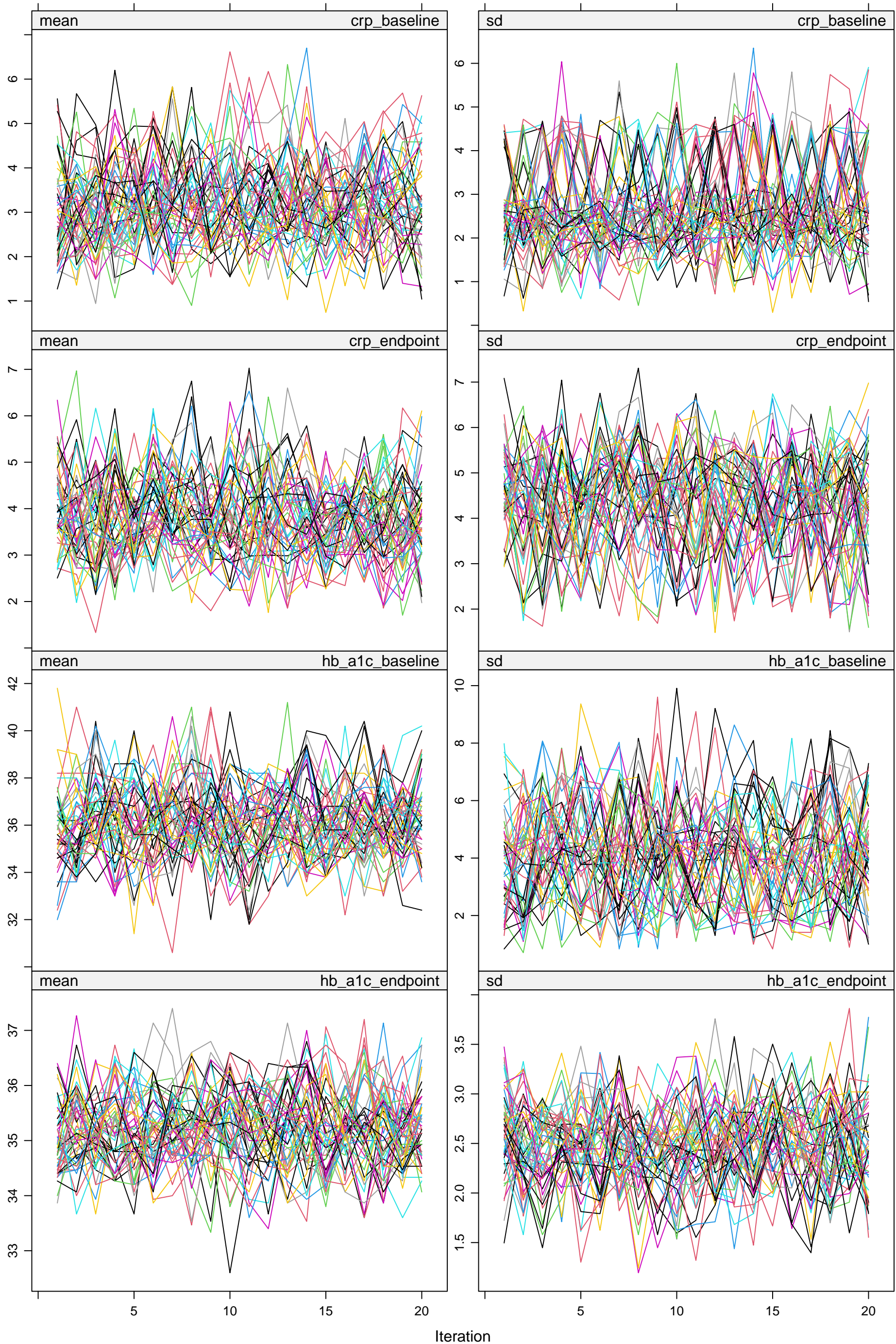

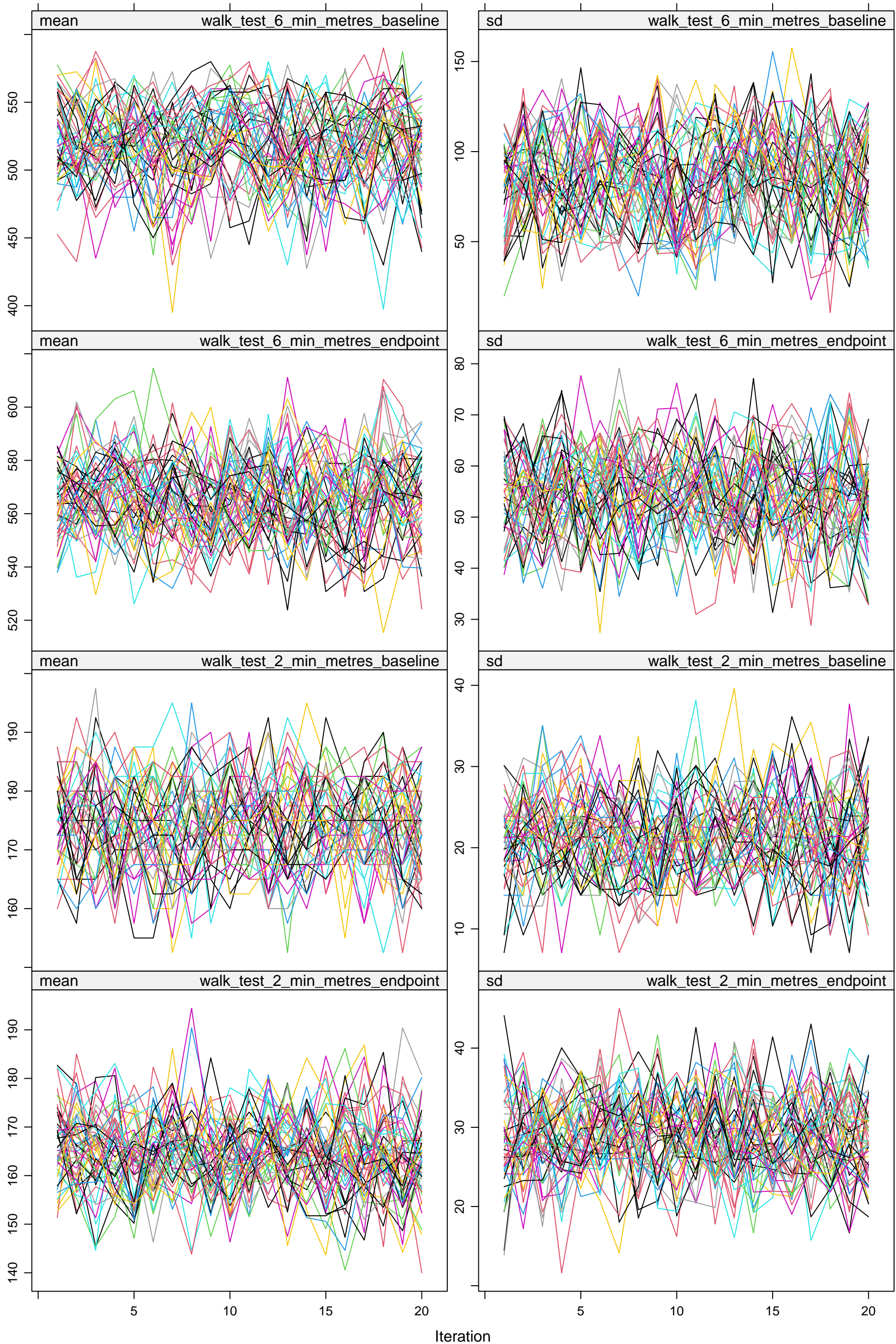

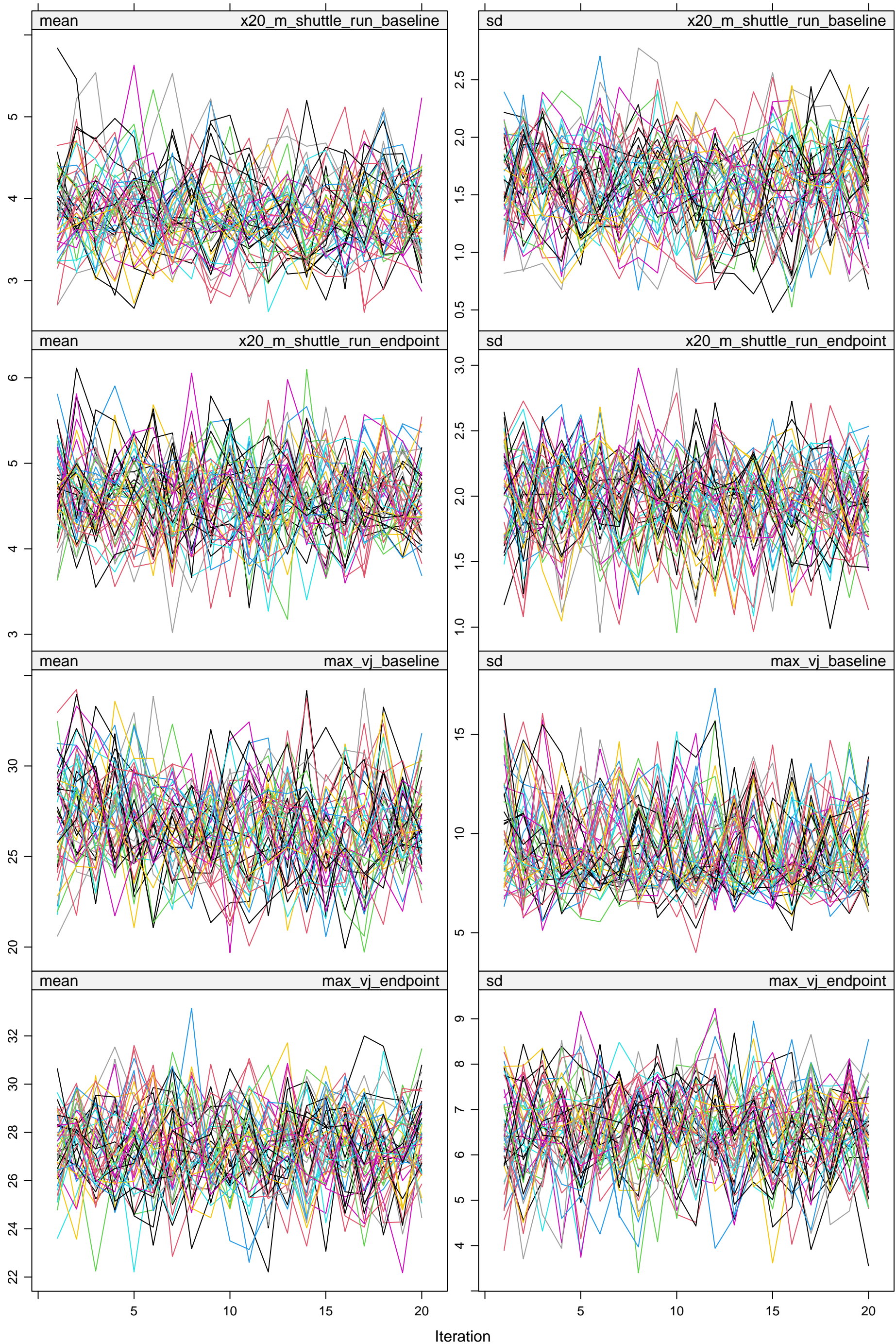

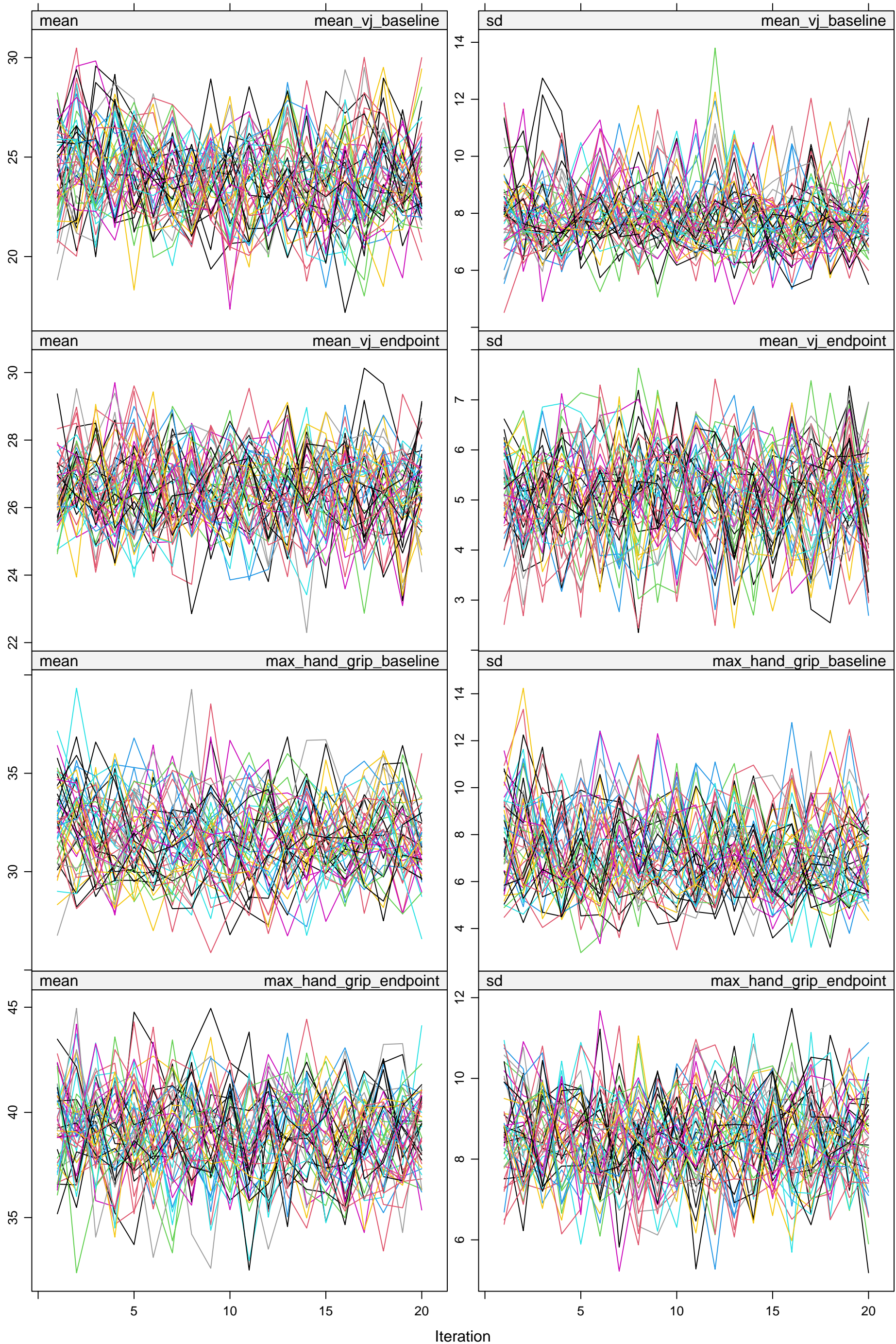

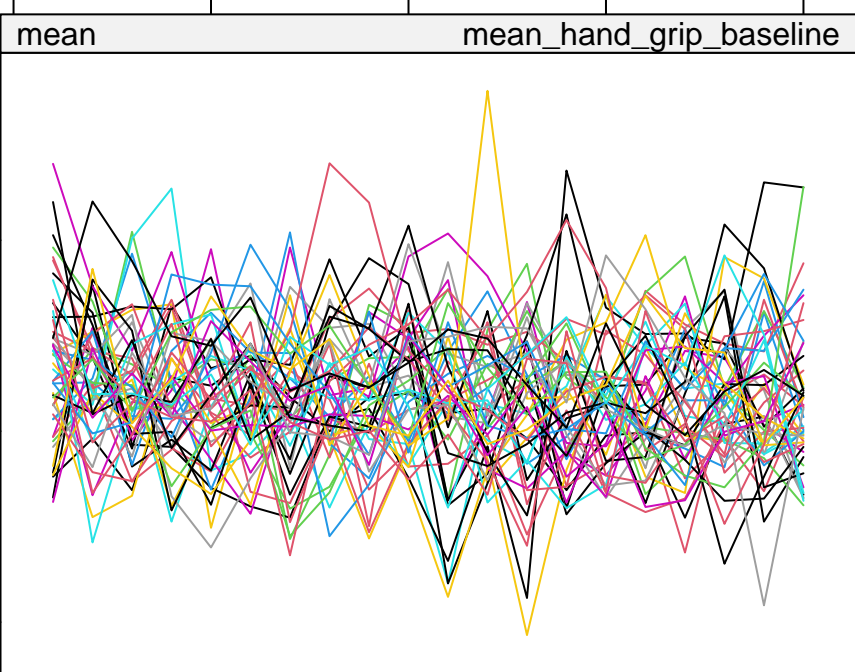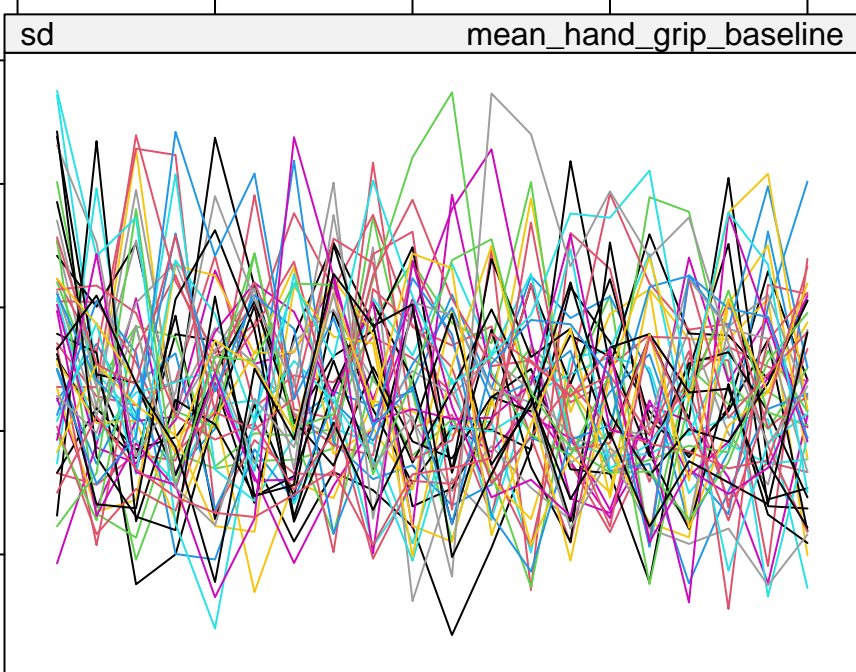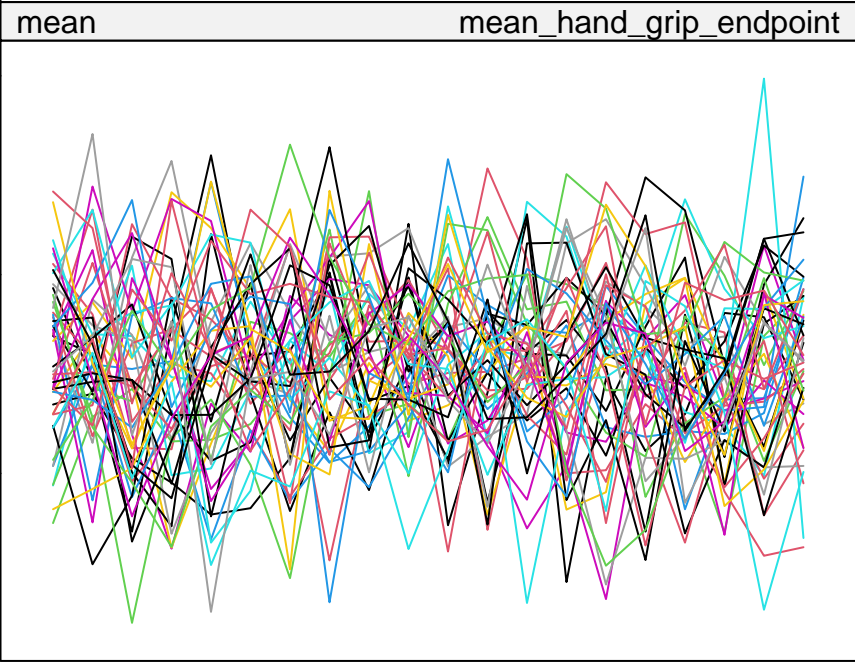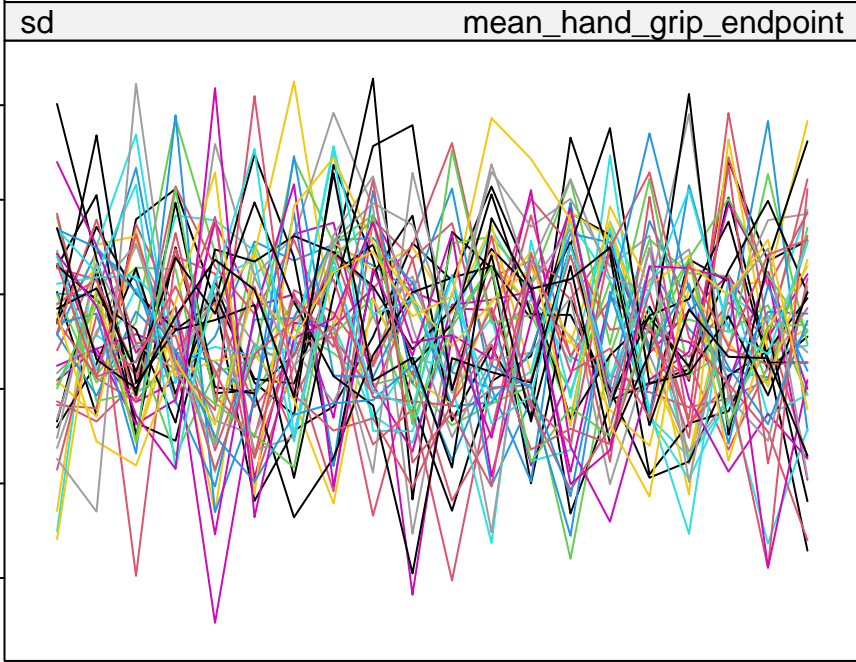

Iteration

Supplement: Supplementary file 1 — Figure X. Trace plots for imputed data. [file EIP-20-0-s002.pdf]
