# Supplementary material for: A Randomised Feasibility Trial of a Service‐Coordinated Exercise Intervention in First‐Episode Psychosis: Challenges in Implementation and Outcome Assessment
Source: Early Interv Psychiatry. 2026 Jul 9;20(7):e70193. doi: 10.1111/eip.70193 (PMC13349341; doi:10.1111/eip.70193)
Supplement: Supplementary file 2 — Figure Y. Distribution plots for imputed data. [file EIP-20-0-s003.pdf]

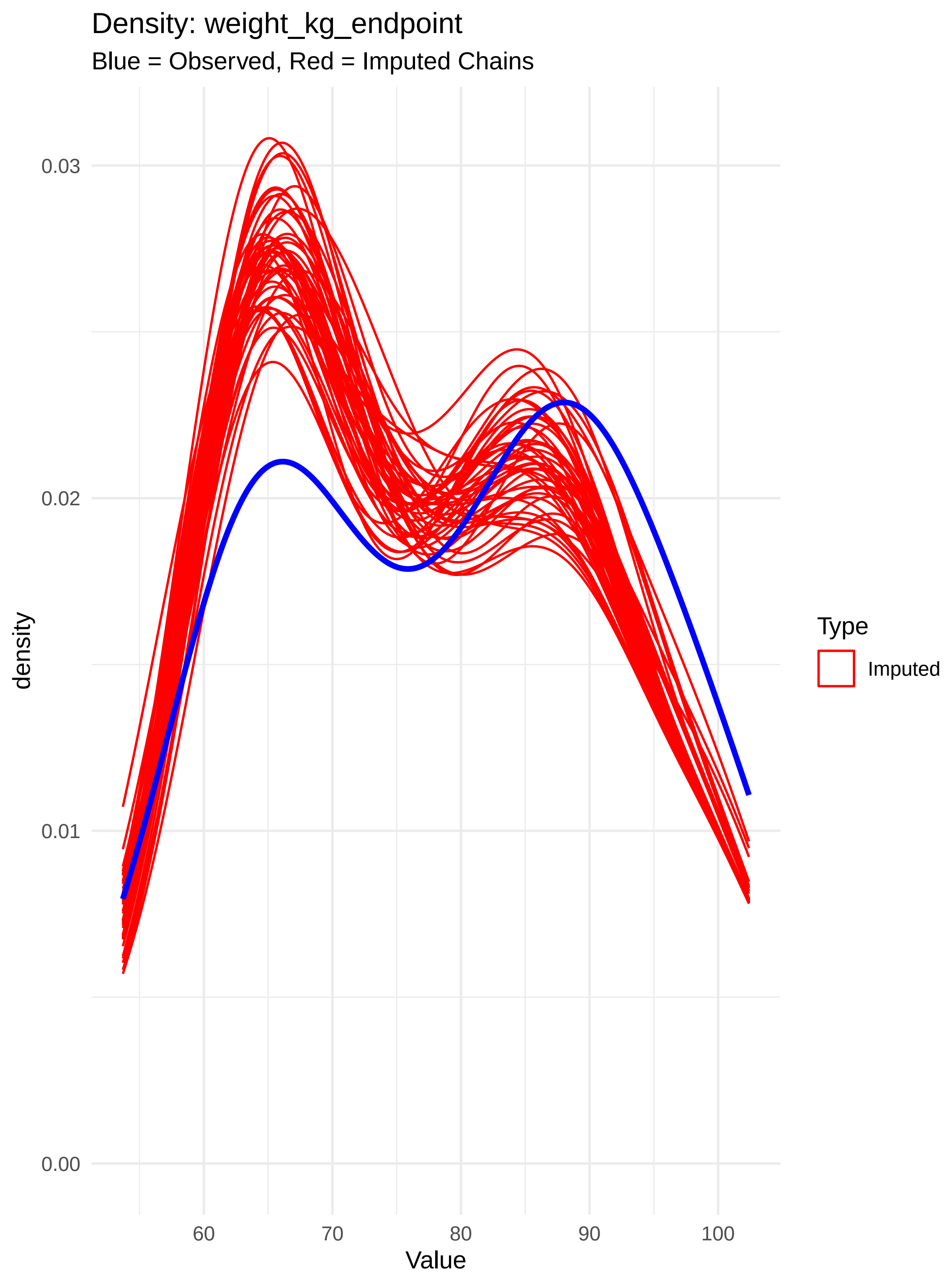
Density: weight_kg_endpoint
Blue = Observed, Red = Imputed Chains
density
0.00
0.01
0.02
0.03
60
70
80
90
100
Value
Type
Imputed

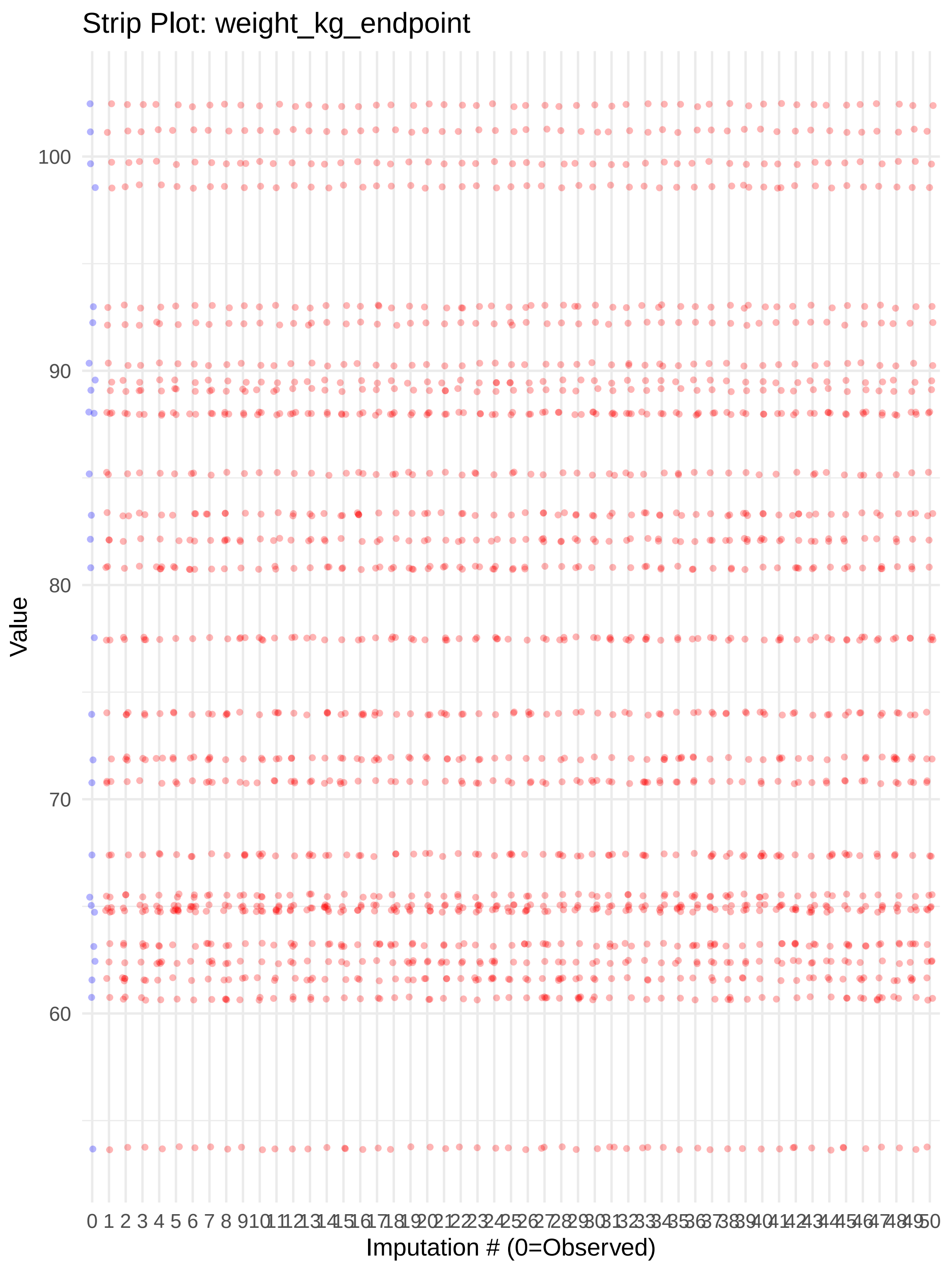
Strip Plot: weight_kg_endpoint
Value
60
70
80
90
100
Imputation # (0=Observed)

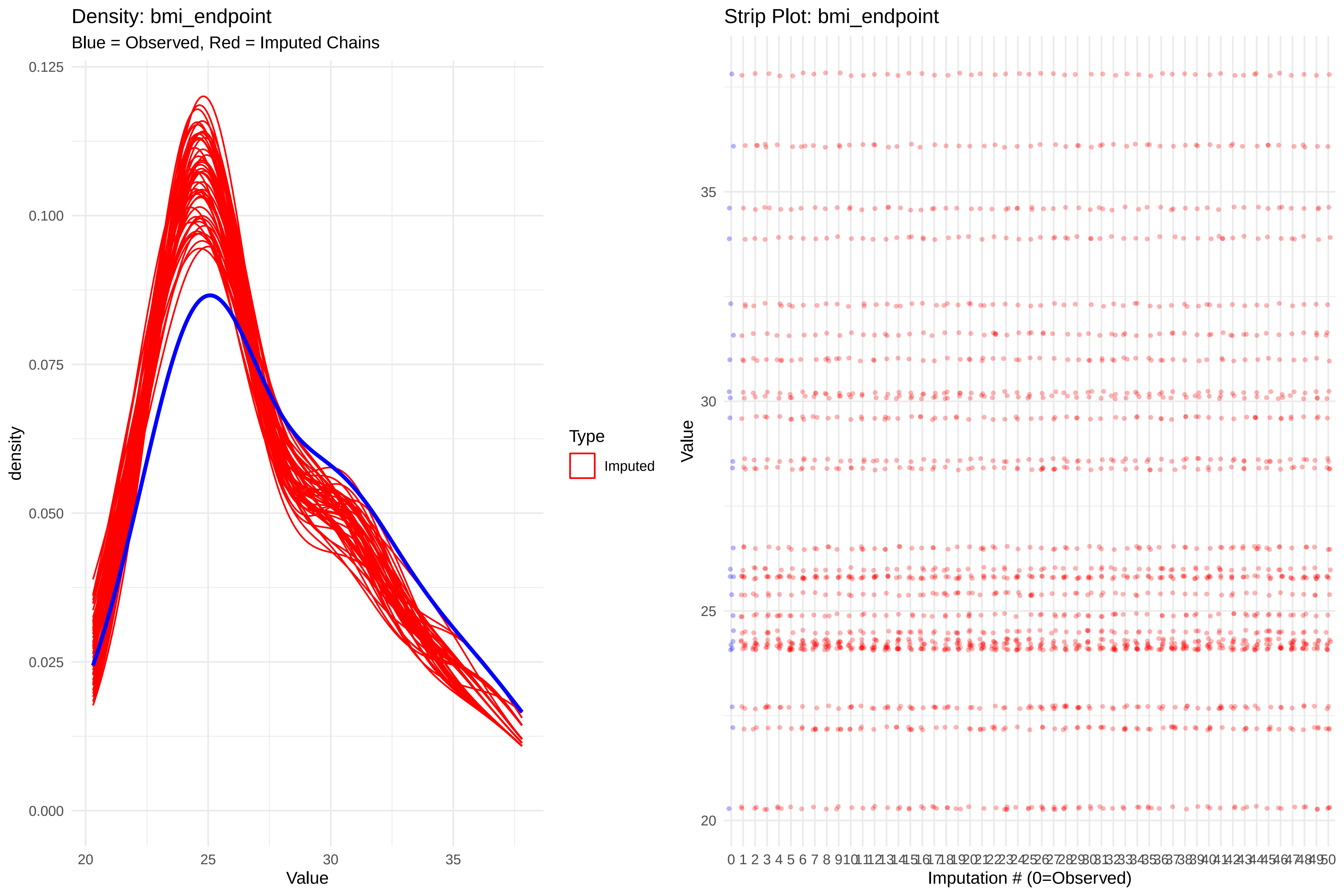

Density: bmi_endpoint
Blue = Observed, Red = Imputed Chains
density
Value
Type
Imputed
Strip Plot: bmi_endpoint
Value
Imputation # (0=Observed)

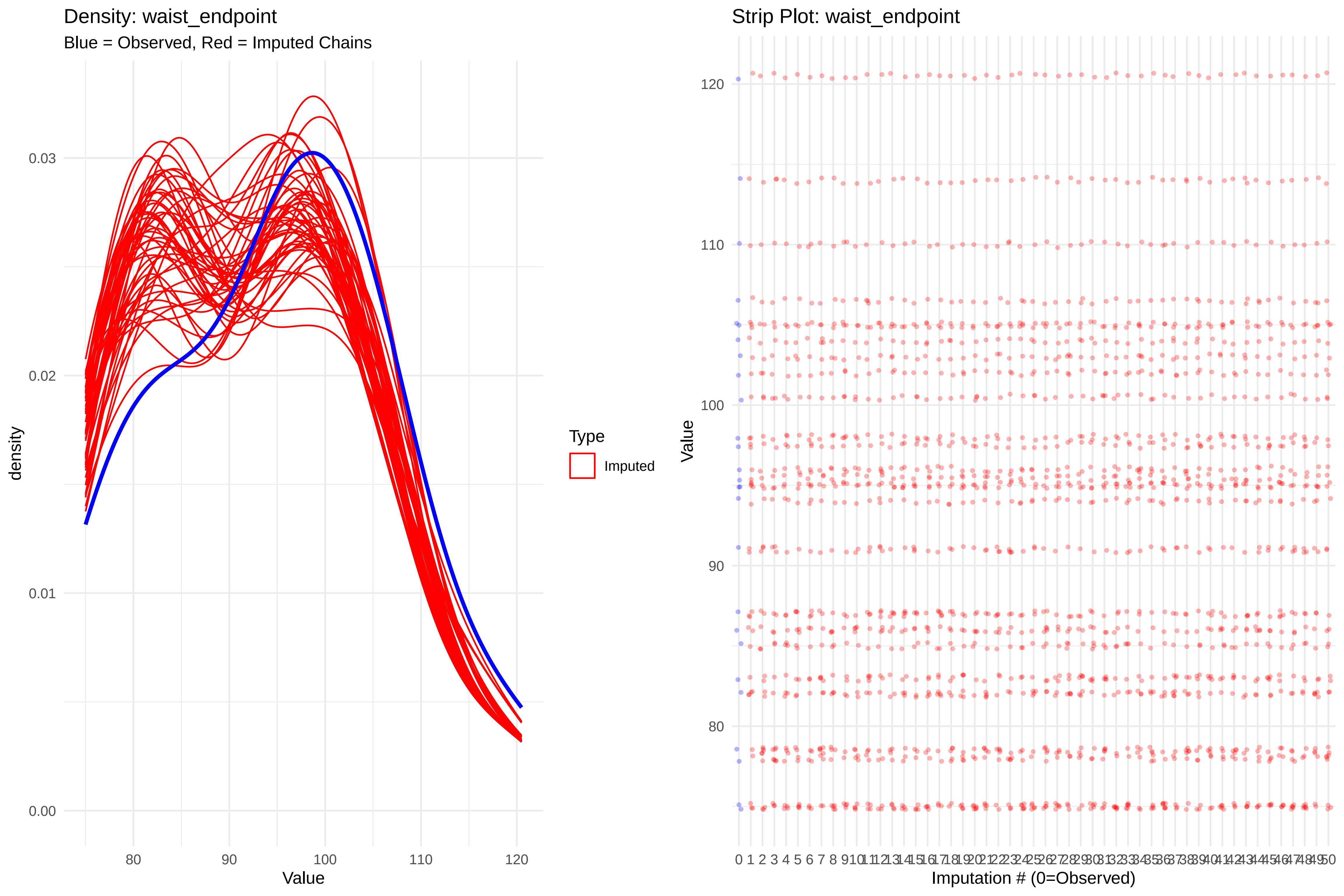

Density: waist_endpoint
Blue = Observed, Red = Imputed Chains
density
Value
Type
Imputed
Strip Plot: waist_endpoint
Value
Imputation # (0=Observed)

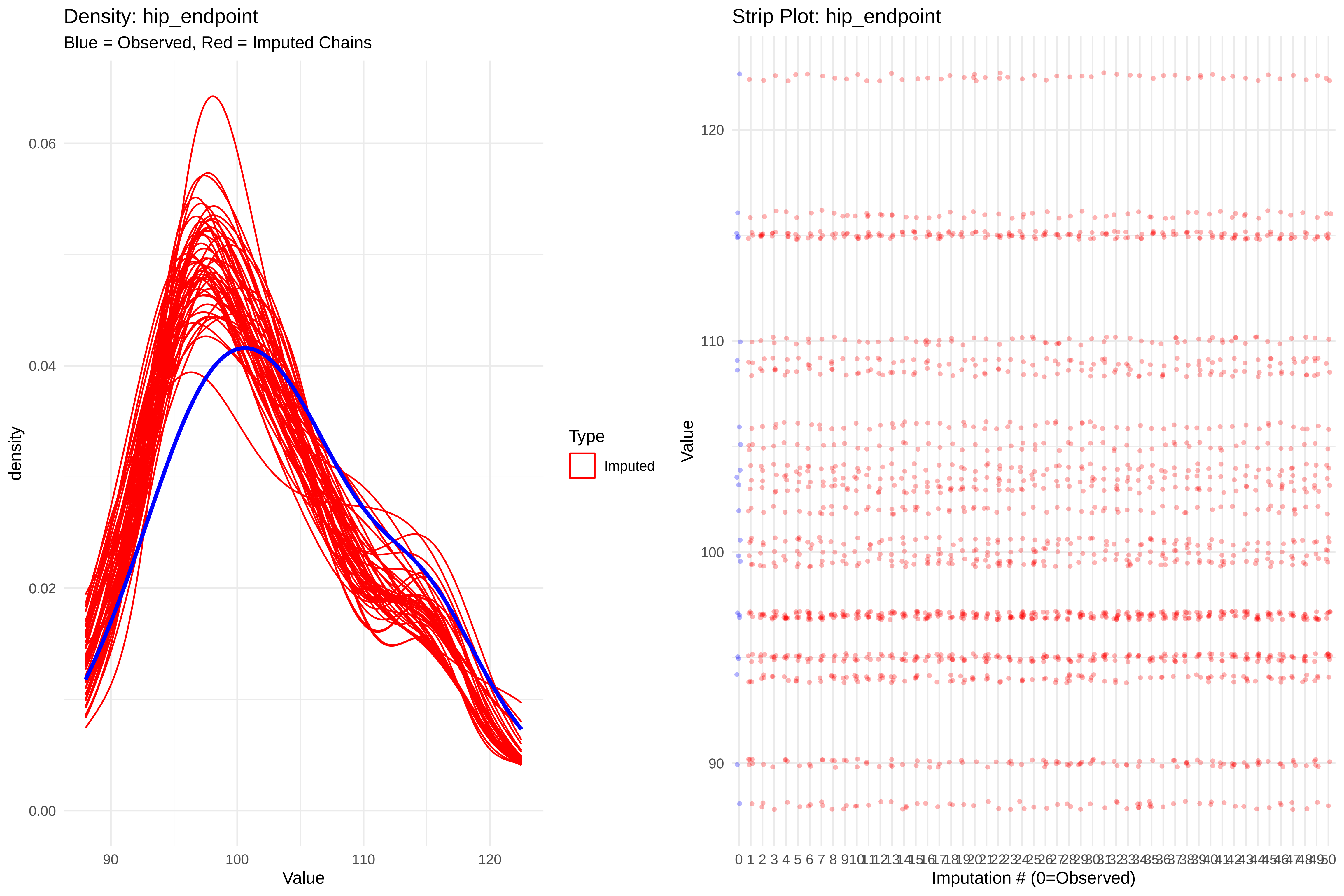

Density: hip_endpoint
Blue = Observed, Red = Imputed Chains
density
Value
Type
Imputed
Strip Plot: hip_endpoint
Value
Imputation # (0=Observed)

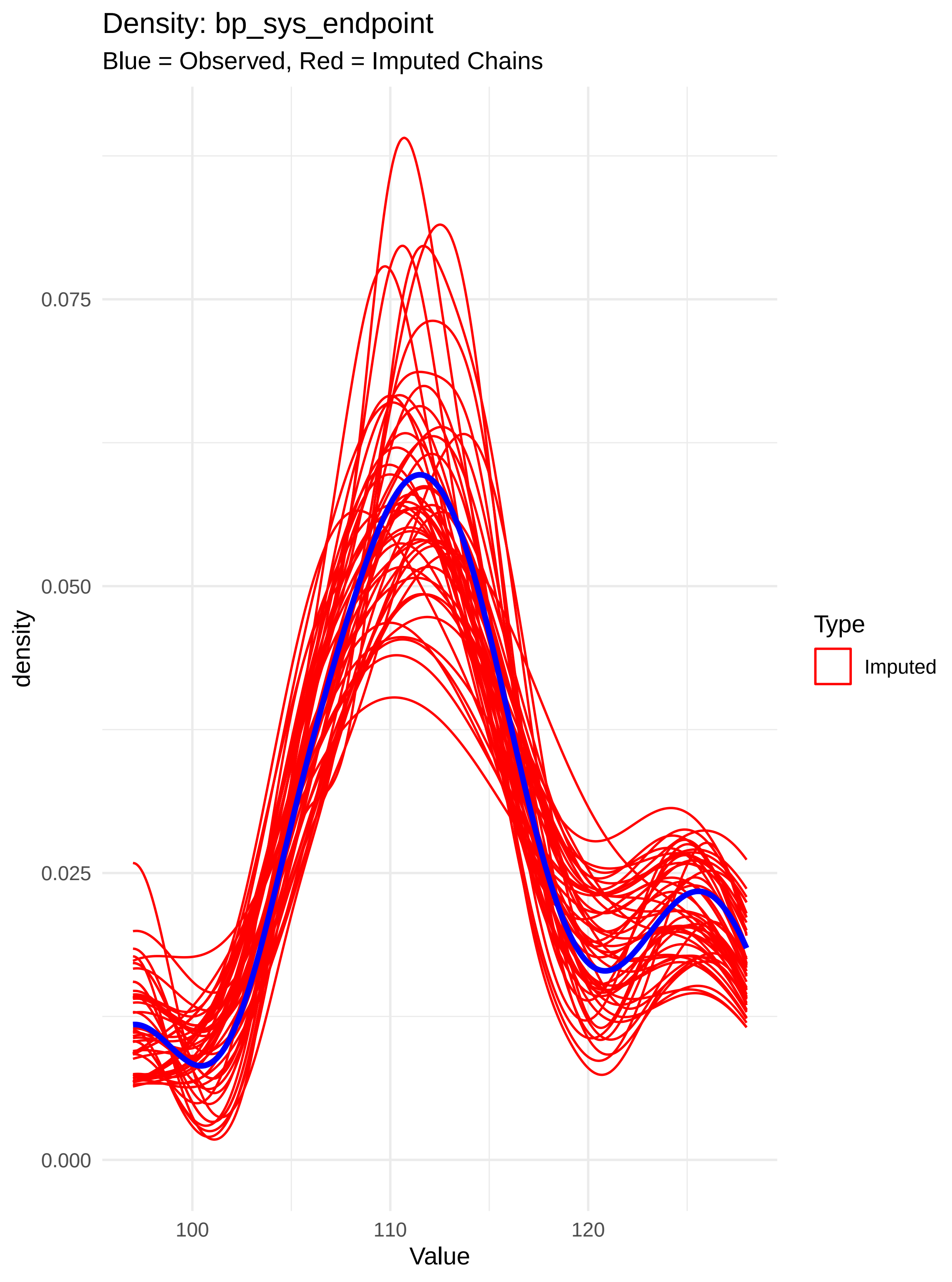
Density: bp_sys_endpoint
Blue = Observed, Red = Imputed Chains
density
0.000
0.025
0.050
0.075
100
110
120
Value
Type
Imputed

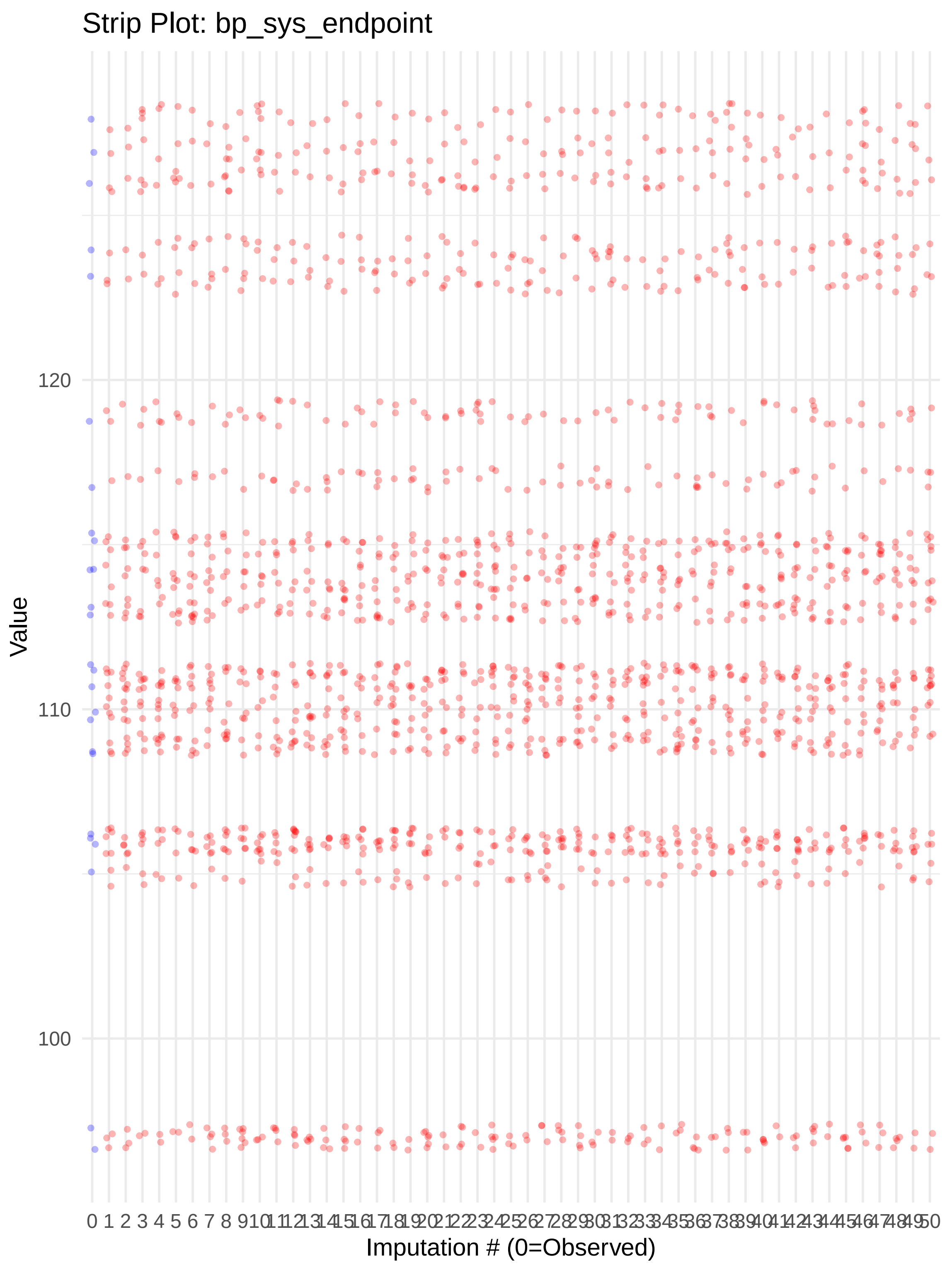
Strip Plot: bp_sys_endpoint
Value
100
110
120
Imputation # (0=Observed)

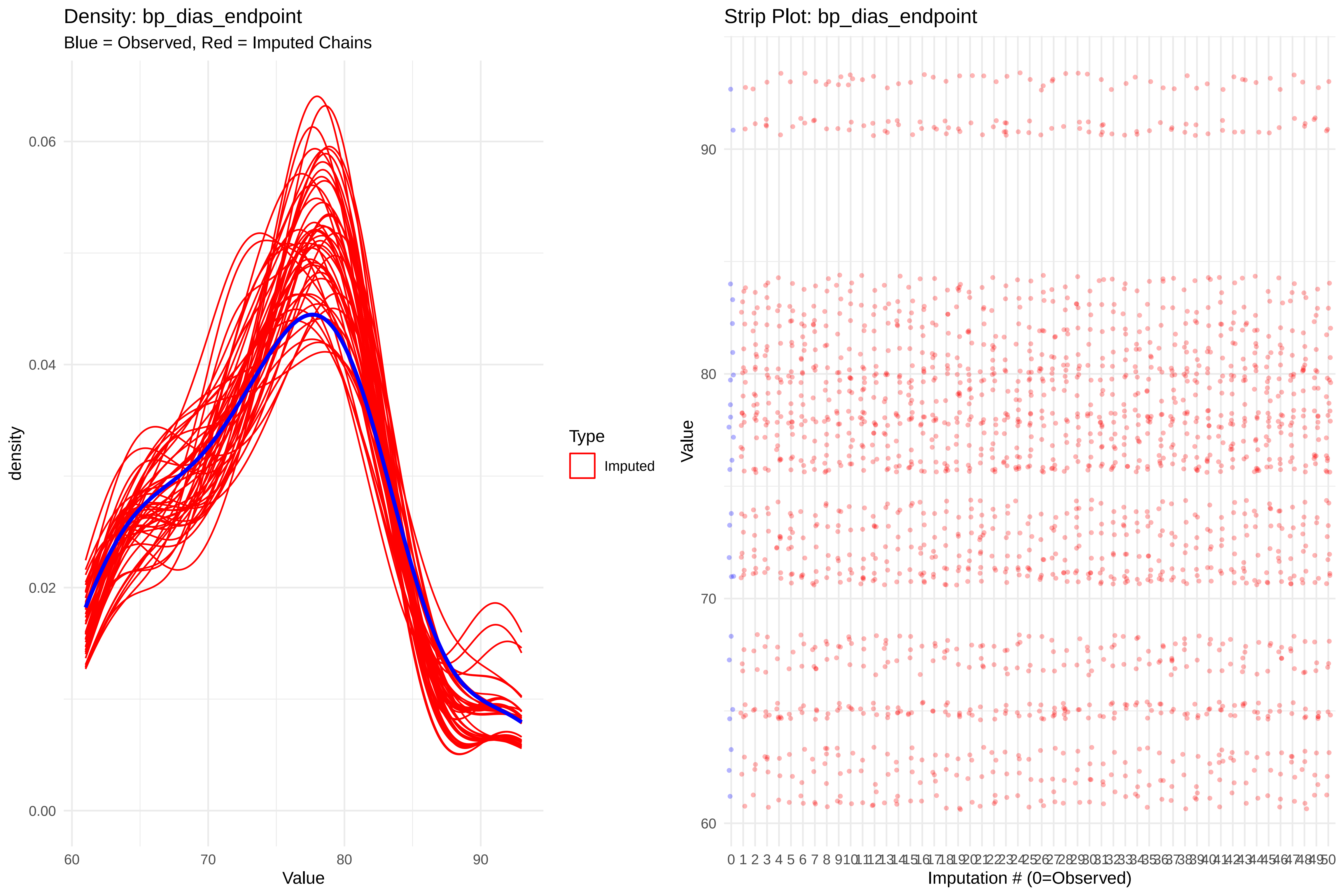

Density: bp_dias_endpoint
Blue = Observed, Red = Imputed Chains
density
0.00
0.02
0.04
0.06
60
70
80
90
Value
Type
Imputed
Strip Plot: bp_dias_endpoint
Value
60
70
80
90
Imputation # (0=Observed)

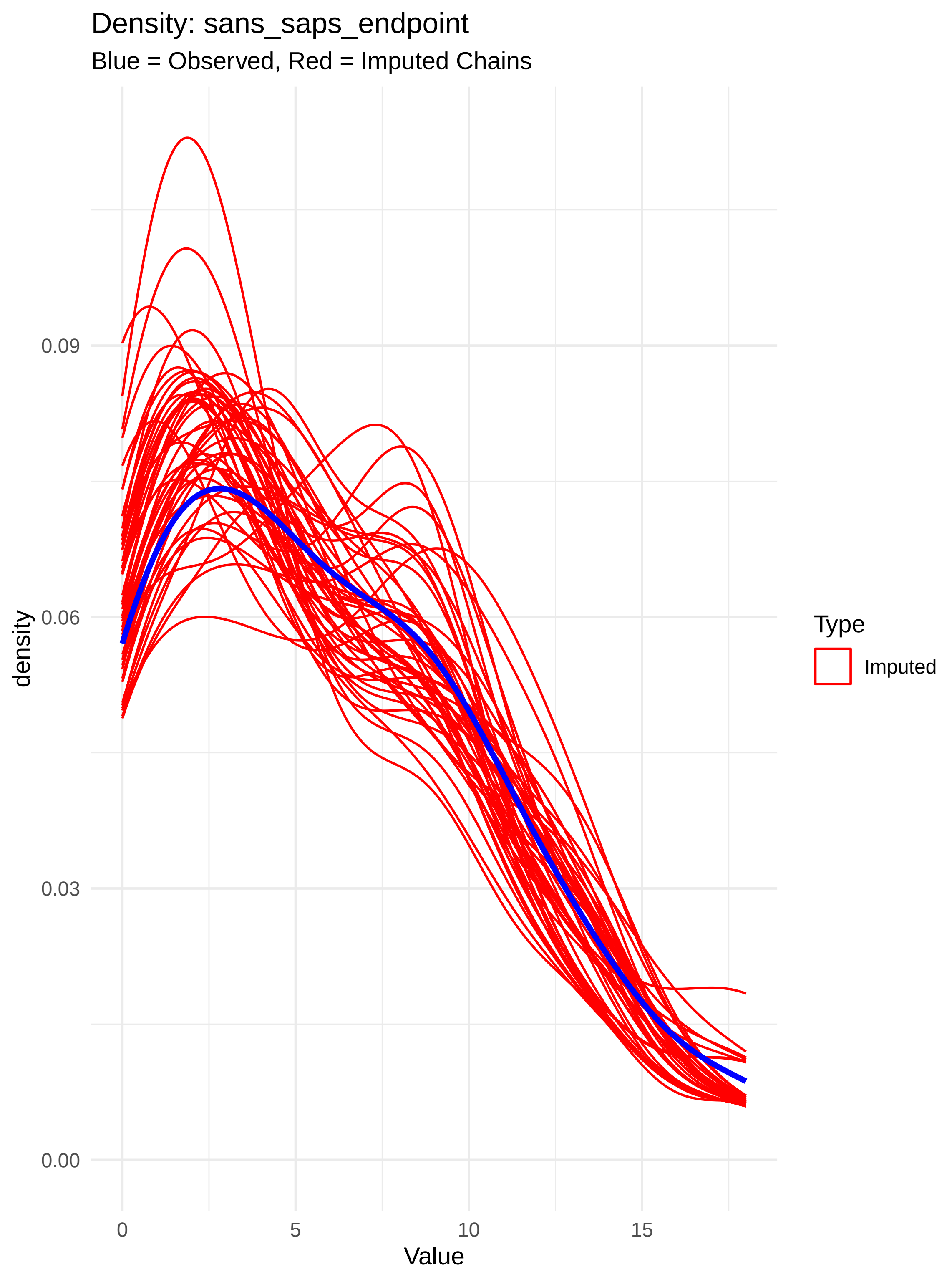
Density: sans_saps_endpoint
Blue = Observed, Red = Imputed Chains
density
0.00
0.03
0.06
0.09
0
5
10
15
Value
Type
Imputed

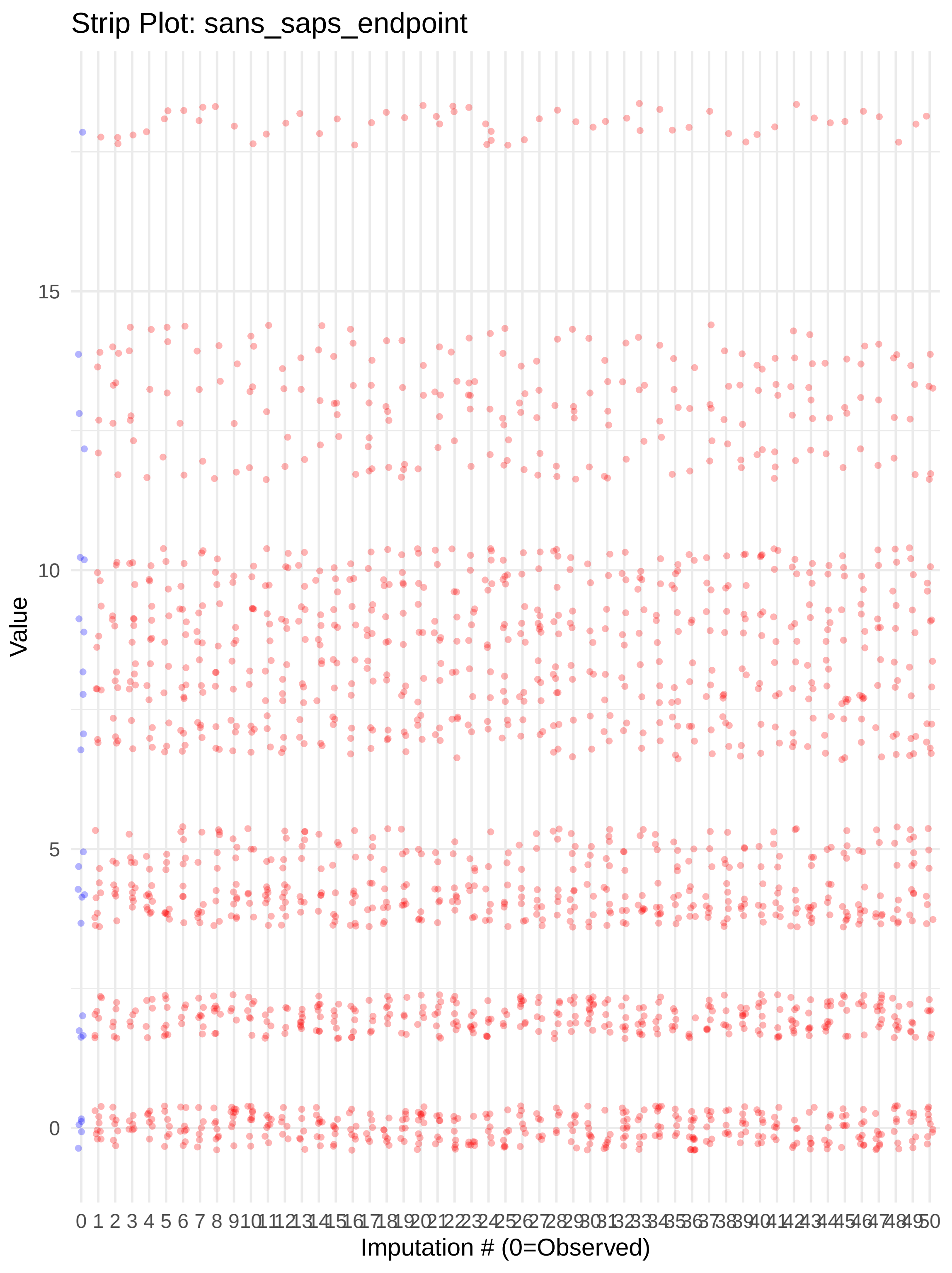
Strip Plot: sans_saps_endpoint
Value
0
5
10
15
Imputation # (0=Observed)

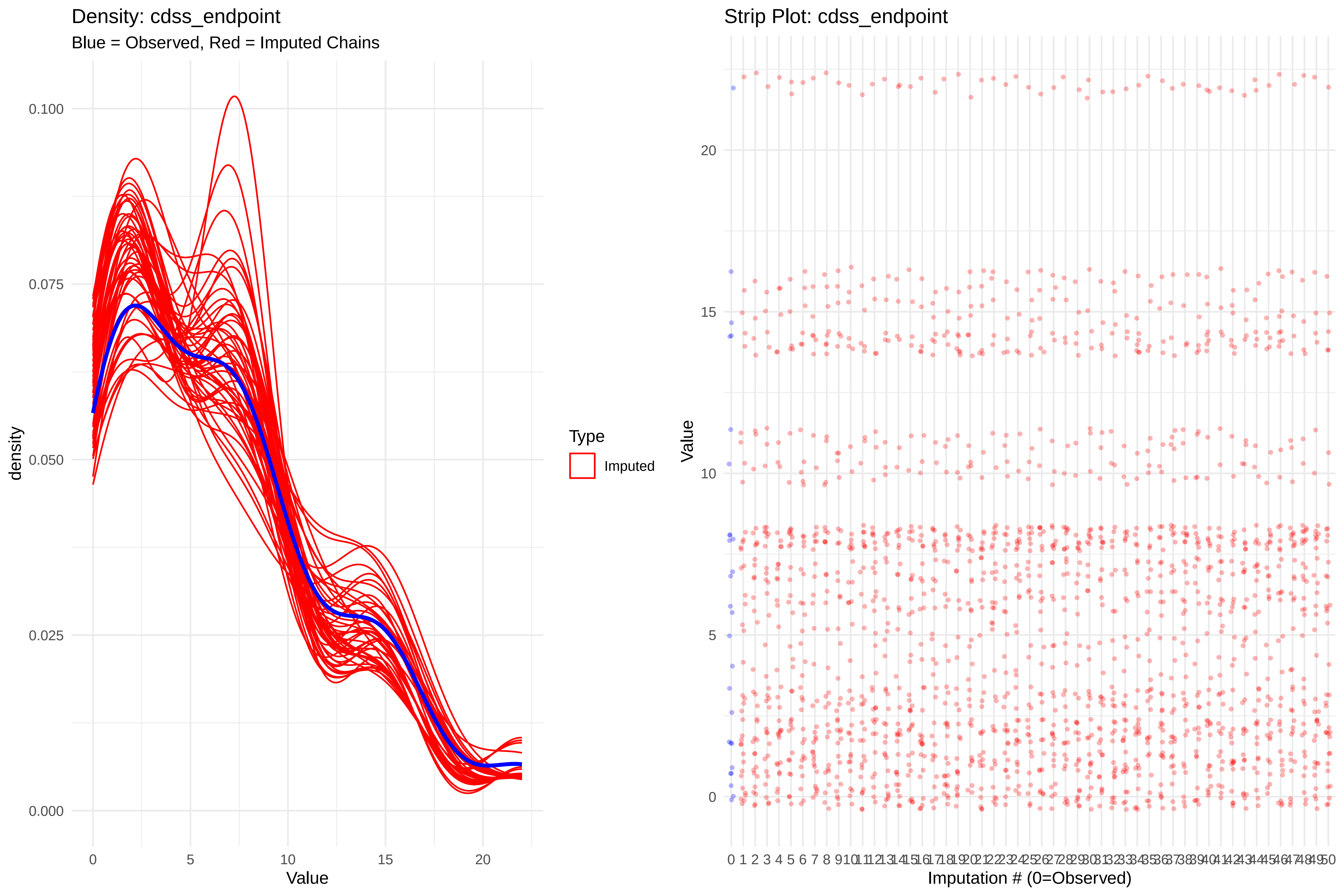
Density: cdss_endpoint
Blue = Observed, Red = Imputed Chains
density
Value
Type
Imputed
Strip Plot: cdss_endpoint
Value
Imputation # (0=Observed)

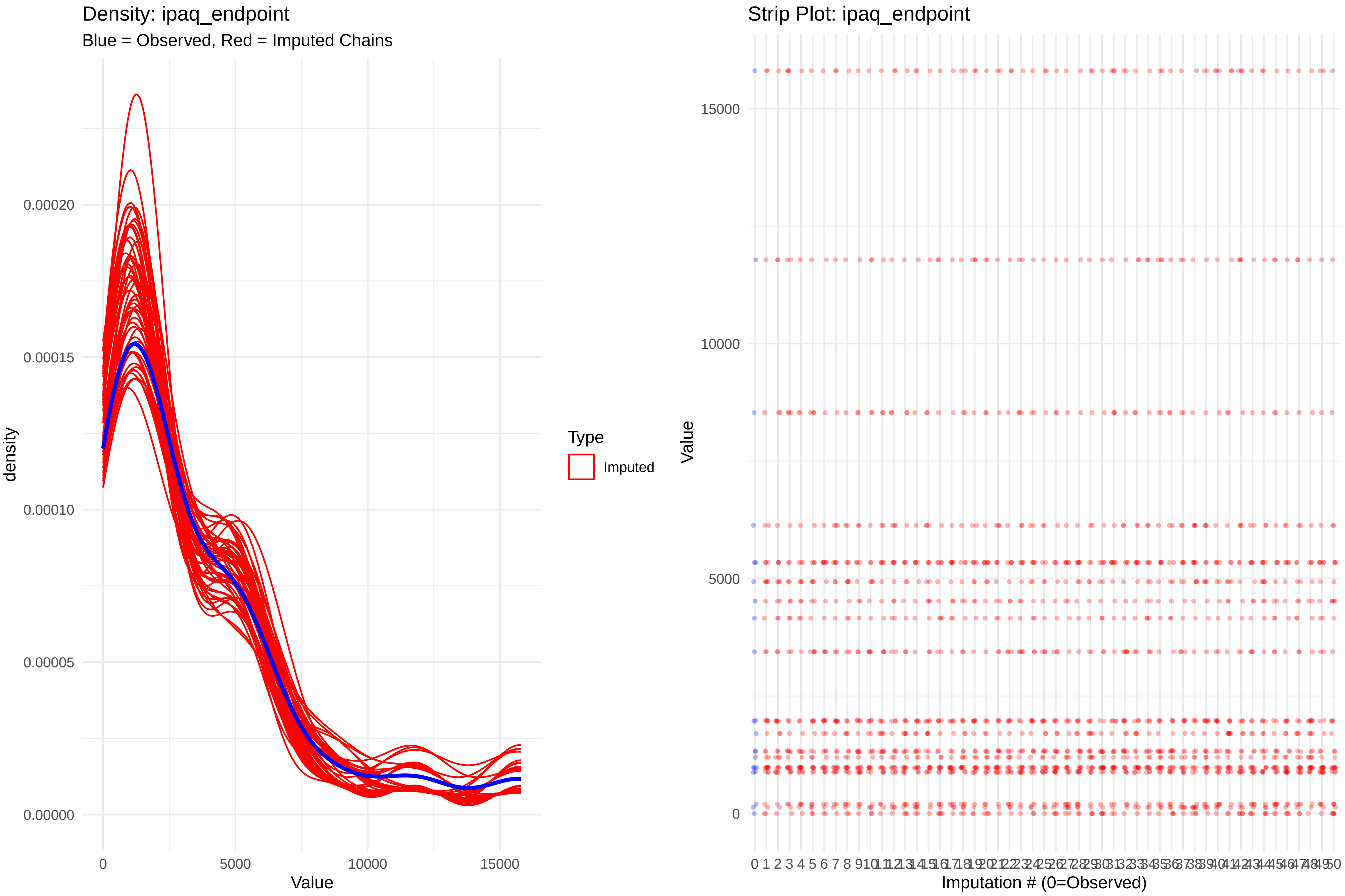
Density: ipaq_endpoint
Blue = Observed, Red = Imputed Chains
density
0.00000
0.00005
0.00010
0.00015
0.00020
0
5000
10000
15000
Value
Type
Imputed
Strip Plot: ipaq_endpoint
Value
0
5000
10000
15000
Imputation # (0=Observed)

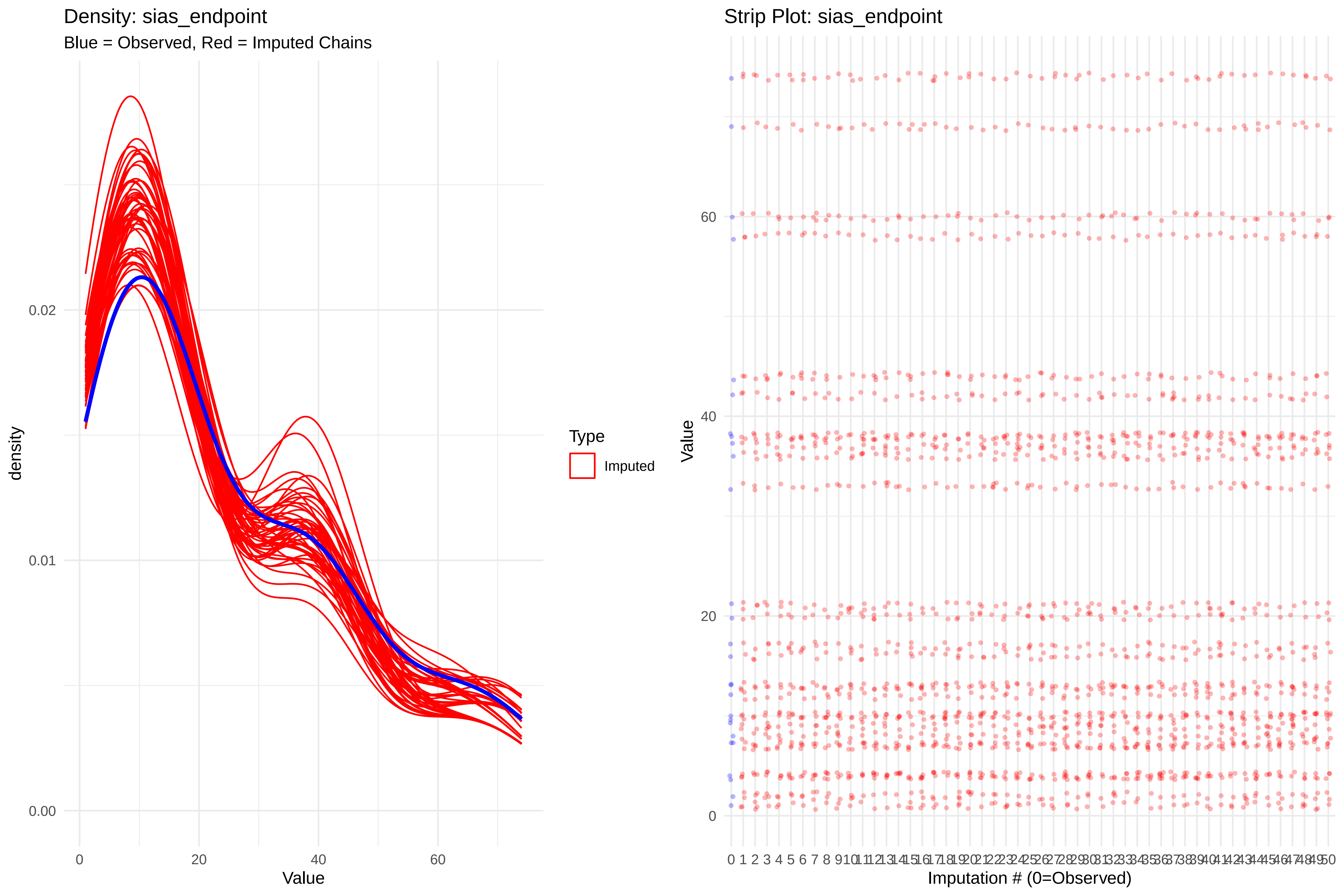
Density: sias_endpoint
Blue = Observed, Red = Imputed Chains
density
0.00
0.01
0.02
0
20
40
60
Value
Type
Imputed
Strip Plot: sias_endpoint
Value
0
20
40
60
Imputation # (0=Observed)

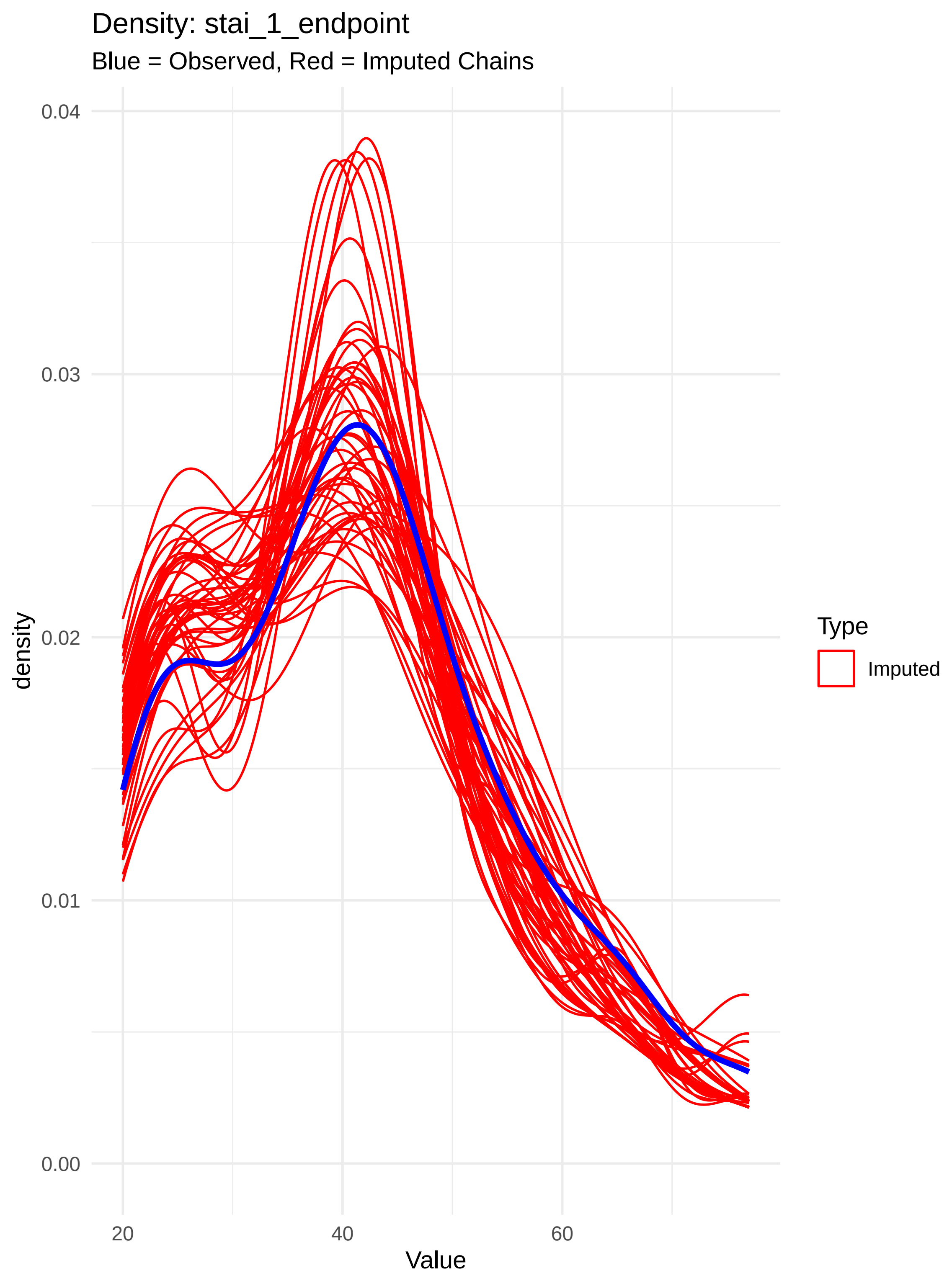
Density: stai_1_endpoint
Blue = Observed, Red = Imputed Chains
0.04
0.03
0.02
0.01
0.00
density
20
40
60
Value
Type
Imputed
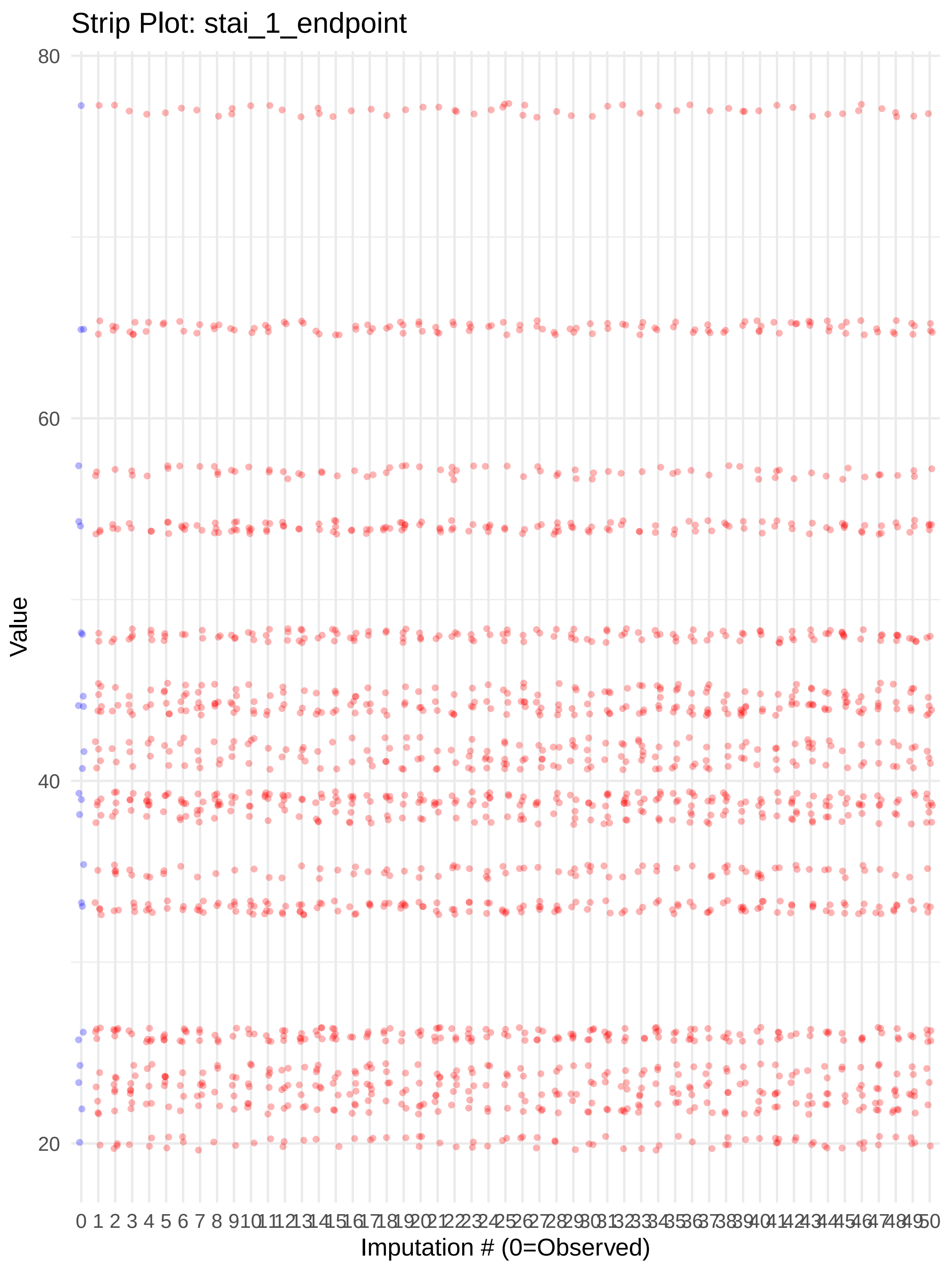
Strip Plot: stai_1_endpoint
80
60
40
20
Value
Imputation # (0=Observed)

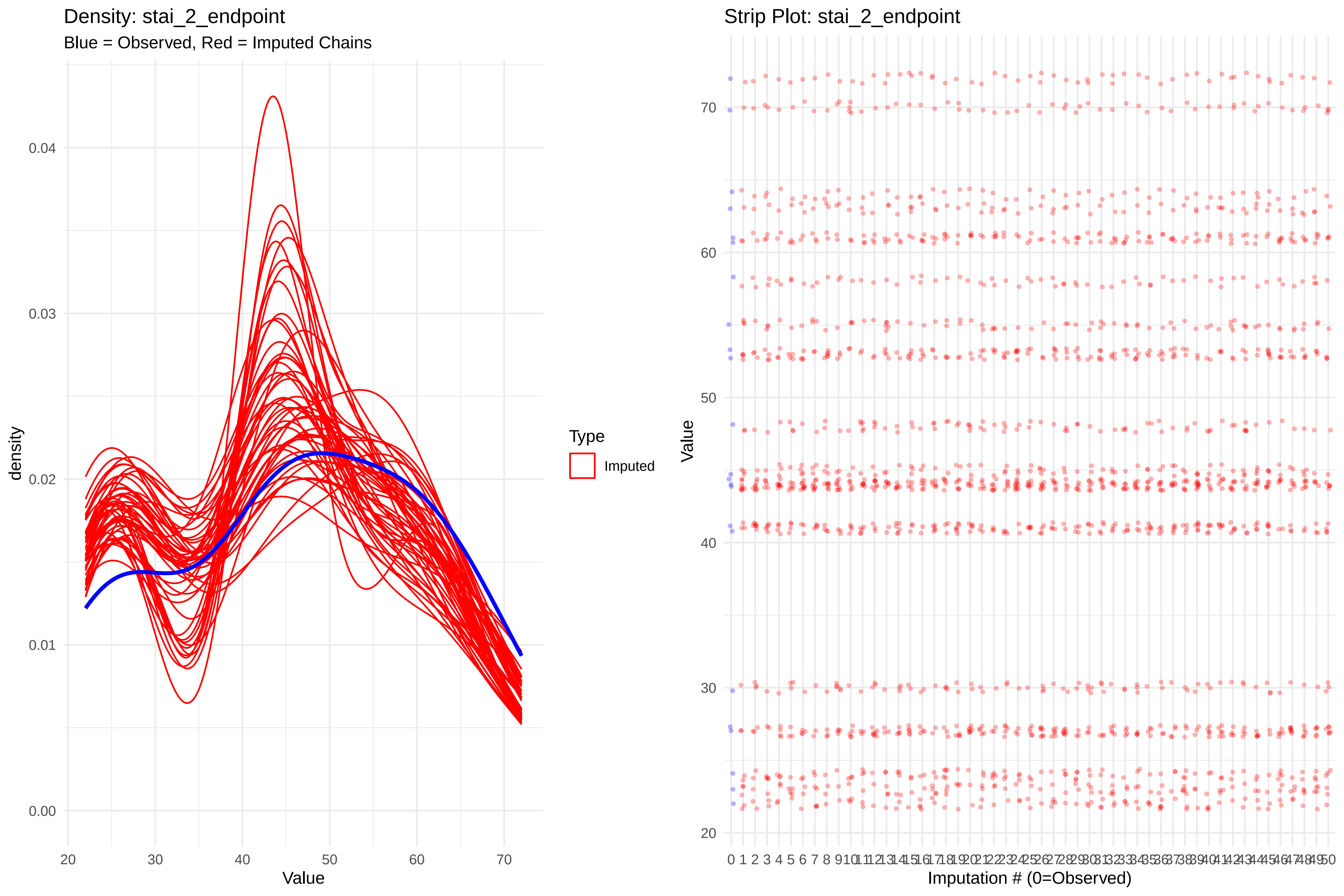

Density: stai_2_endpoint
Blue = Observed, Red = Imputed Chains
density
Value
Type
Imputed
Strip Plot: stai_2_endpoint
Value
Imputation # (0=Observed)

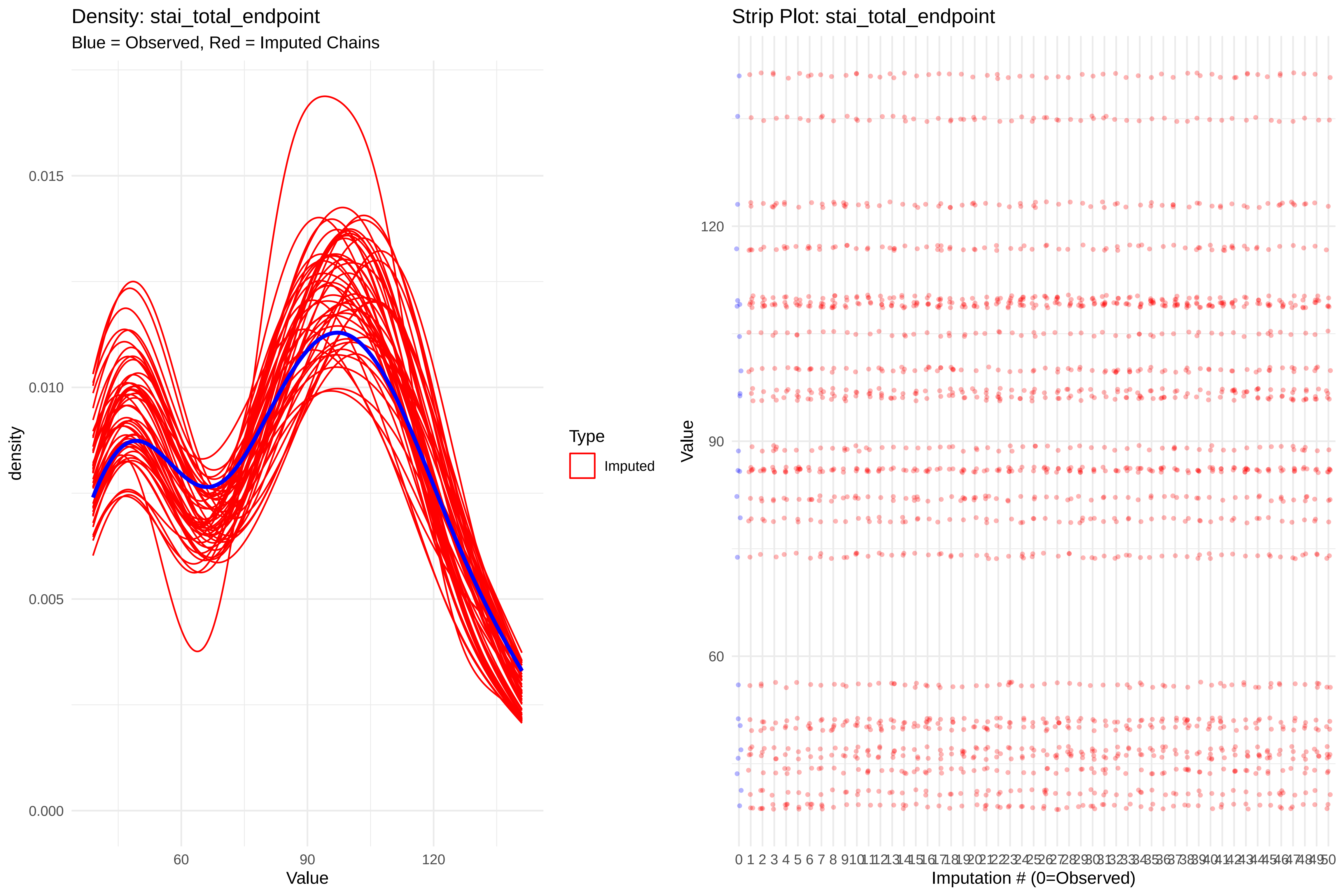
Density: stai_total_endpoint
Blue = Observed, Red = Imputed Chains
density
0.015
0.010
0.005
0.000
60
90
120
Value
Type
Imputed
Strip Plot: stai_total_endpoint
Value
120
90
60
Imputation # (0=Observed)

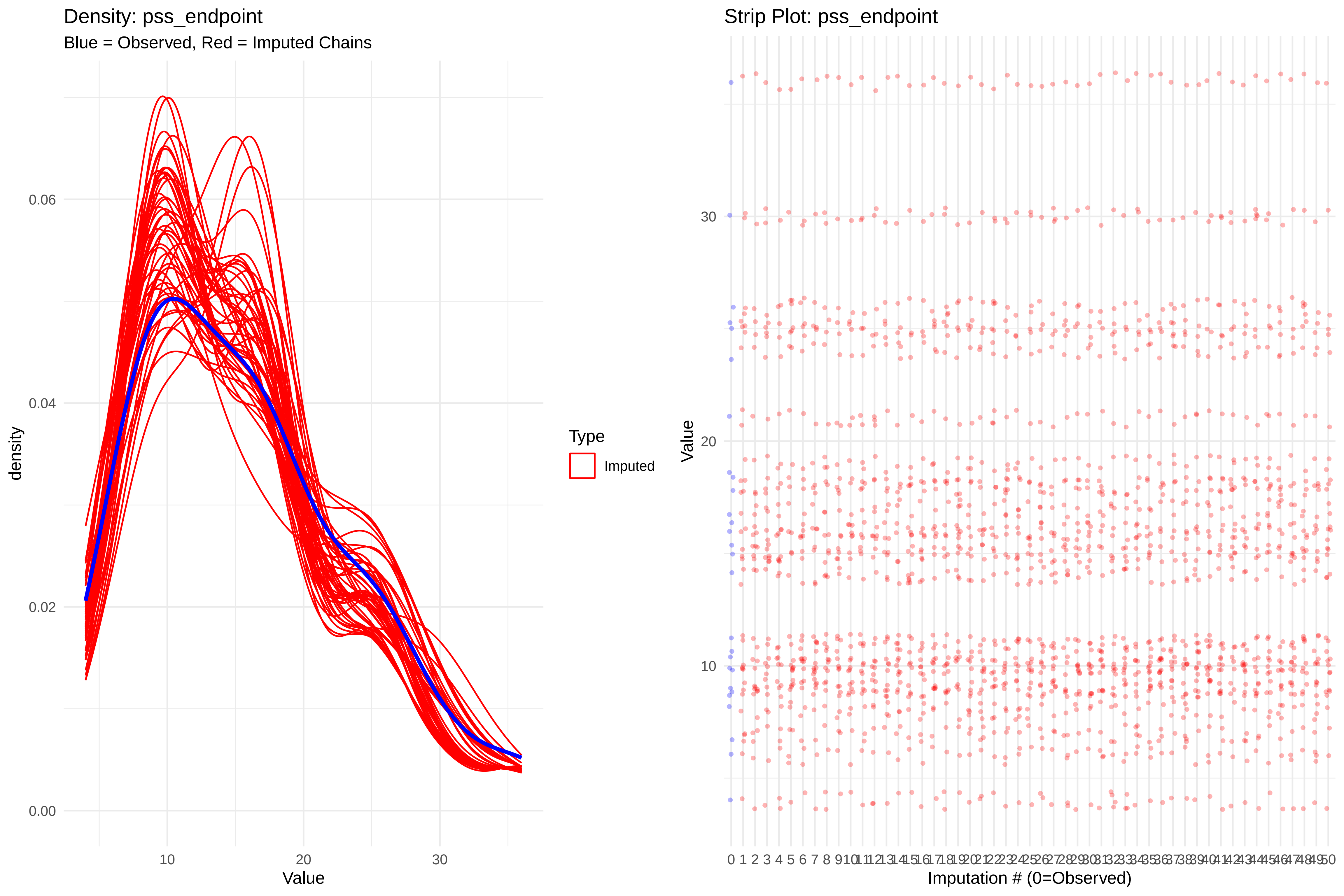

Density: pss_endpoint
Blue = Observed, Red = Imputed Chains
density
0.00
0.02
0.04
0.06
10
20
30
Value
Type
Imputed
Strip Plot: pss_endpoint
Value
10
20
30
Imputation # (0=Observed)

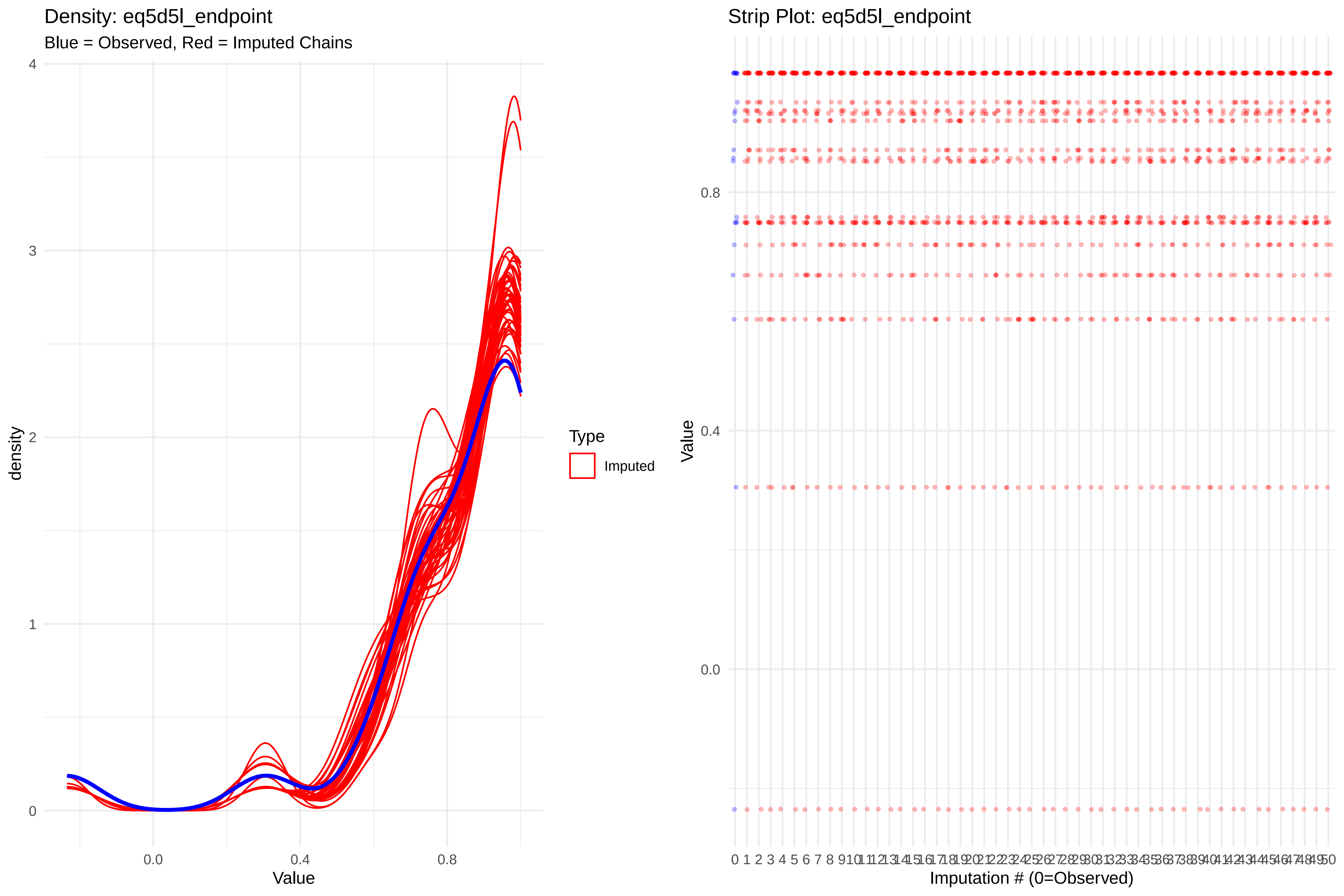

Density: eq5d5l_endpoint
Blue = Observed, Red = Imputed Chains
density
Value
Type
Imputed
Strip Plot: eq5d5l_endpoint
Value
Imputation # (0=Observed)

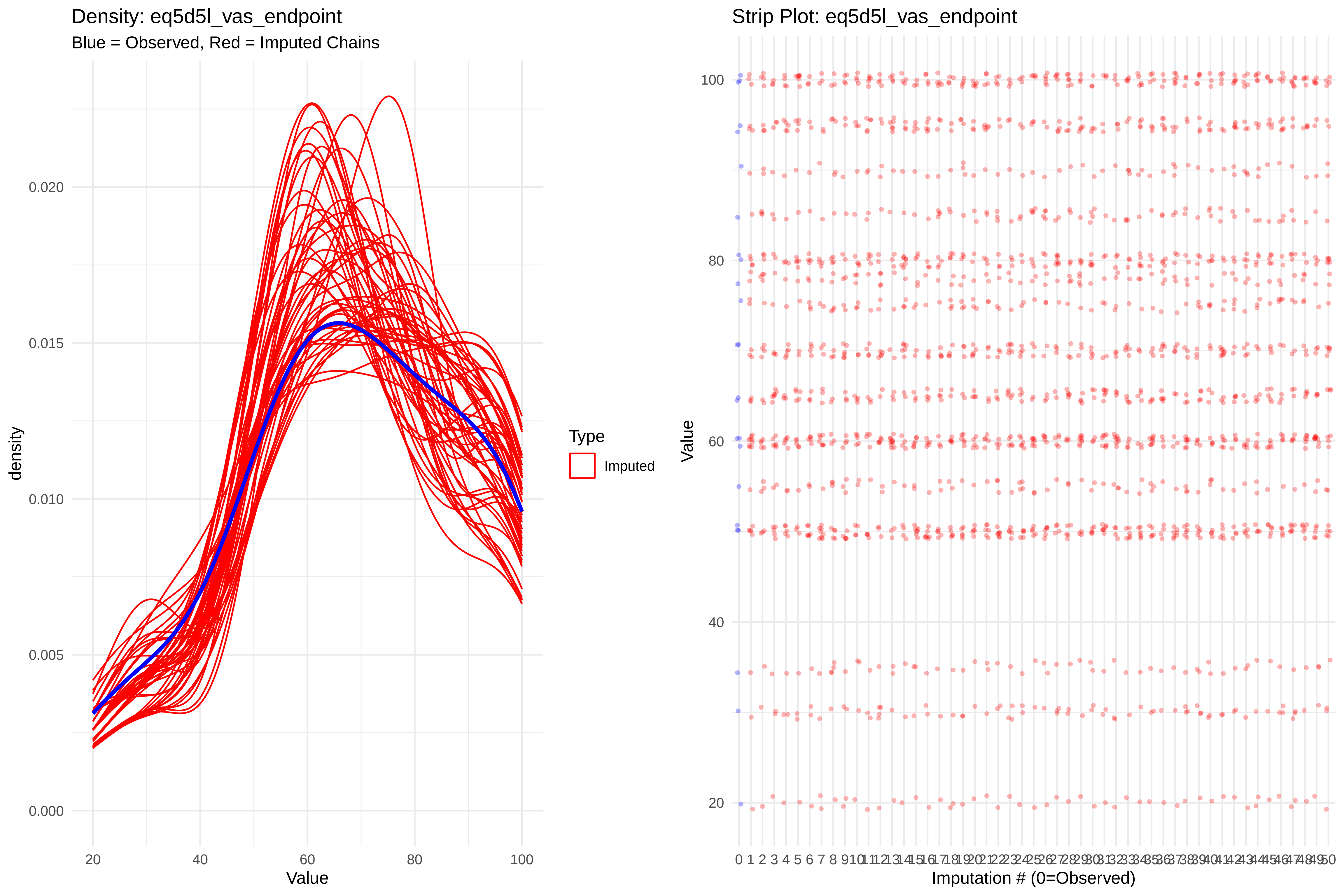

Density: eq5d5l_vas_endpoint
Blue = Observed, Red = Imputed Chains
density
Value
Type
Imputed
0.000
0.005
0.010
0.015
0.020
20
40
60
80
100
Strip Plot: eq5d5l_vas_endpoint
Value
Imputation # (0=Observed)

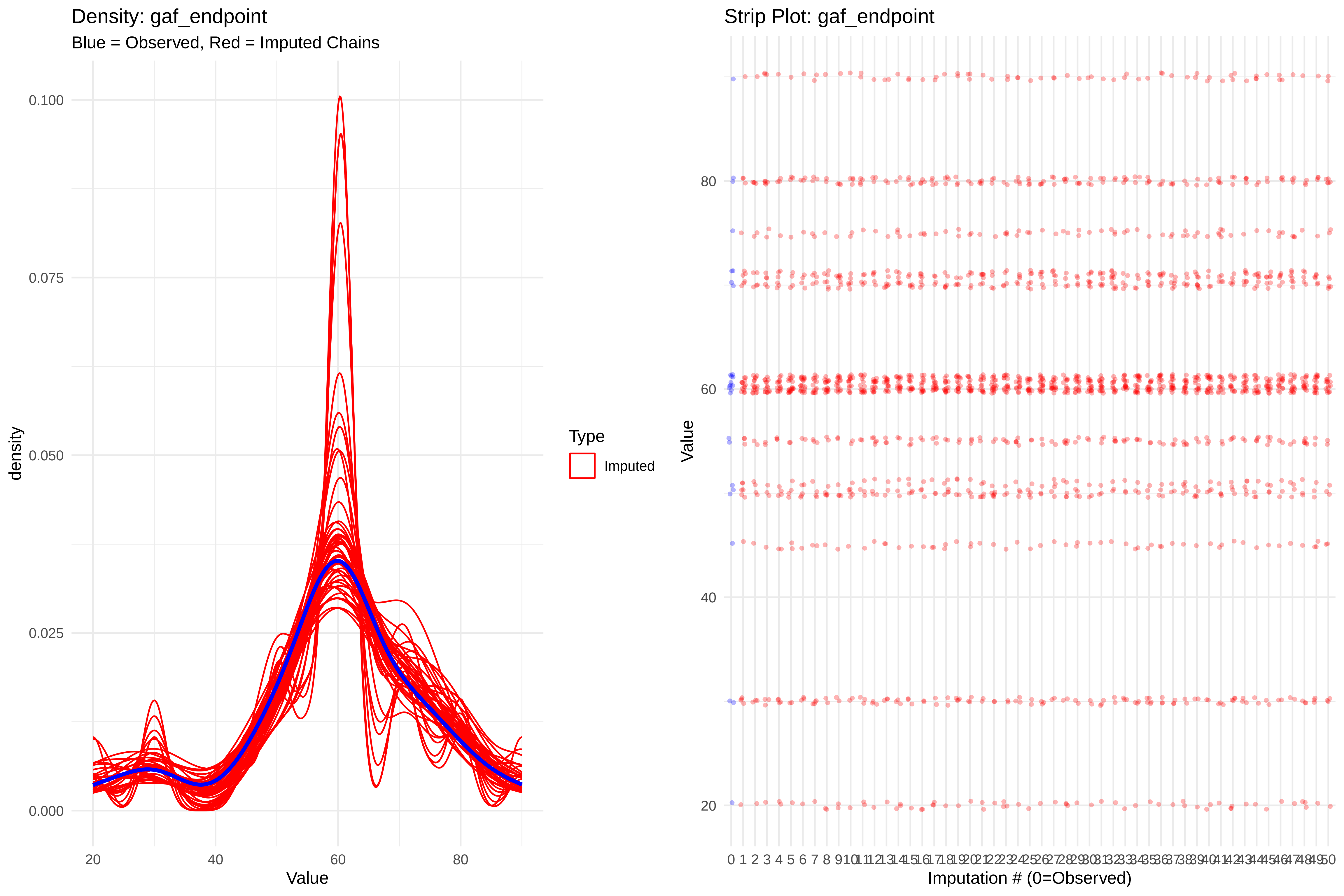

Density: gaf_endpoint
Blue = Observed, Red = Imputed Chains
density
Value
Type
Imputed
0.000
0.025
0.050
0.075
0.100
20
40
60
80
Strip Plot: gaf_endpoint
Value
Imputation # (0=Observed)
20
40
60
80

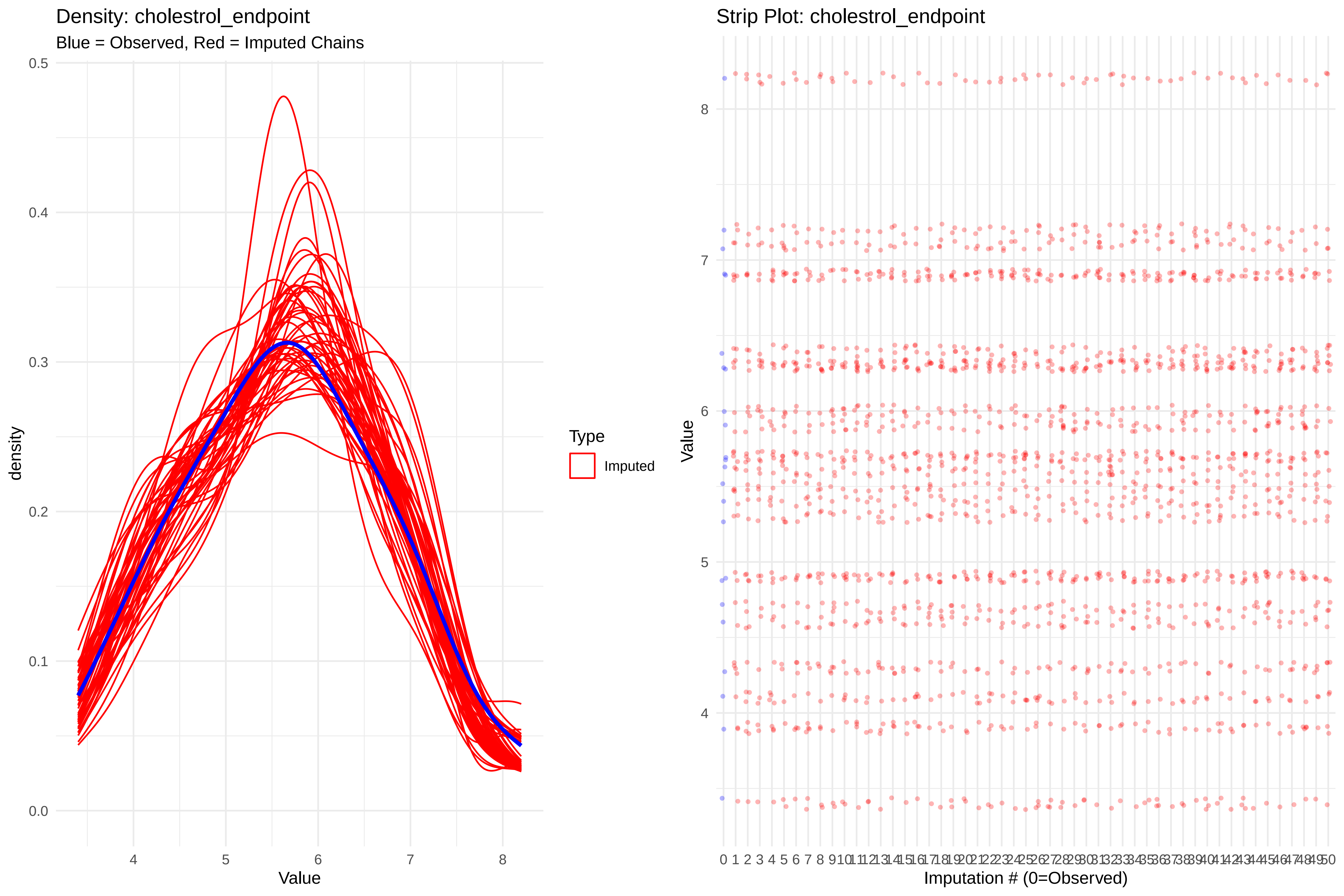
Density: cholestrol_endpoint
Blue = Observed, Red = Imputed Chains
density
0.0
0.1
0.2
0.3
0.4
0.5
4
5
6
7
8
Value
Type
Imputed
Strip Plot: cholestrol_endpoint
Value
4
5
6
7
8
Imputation # (0=Observed)

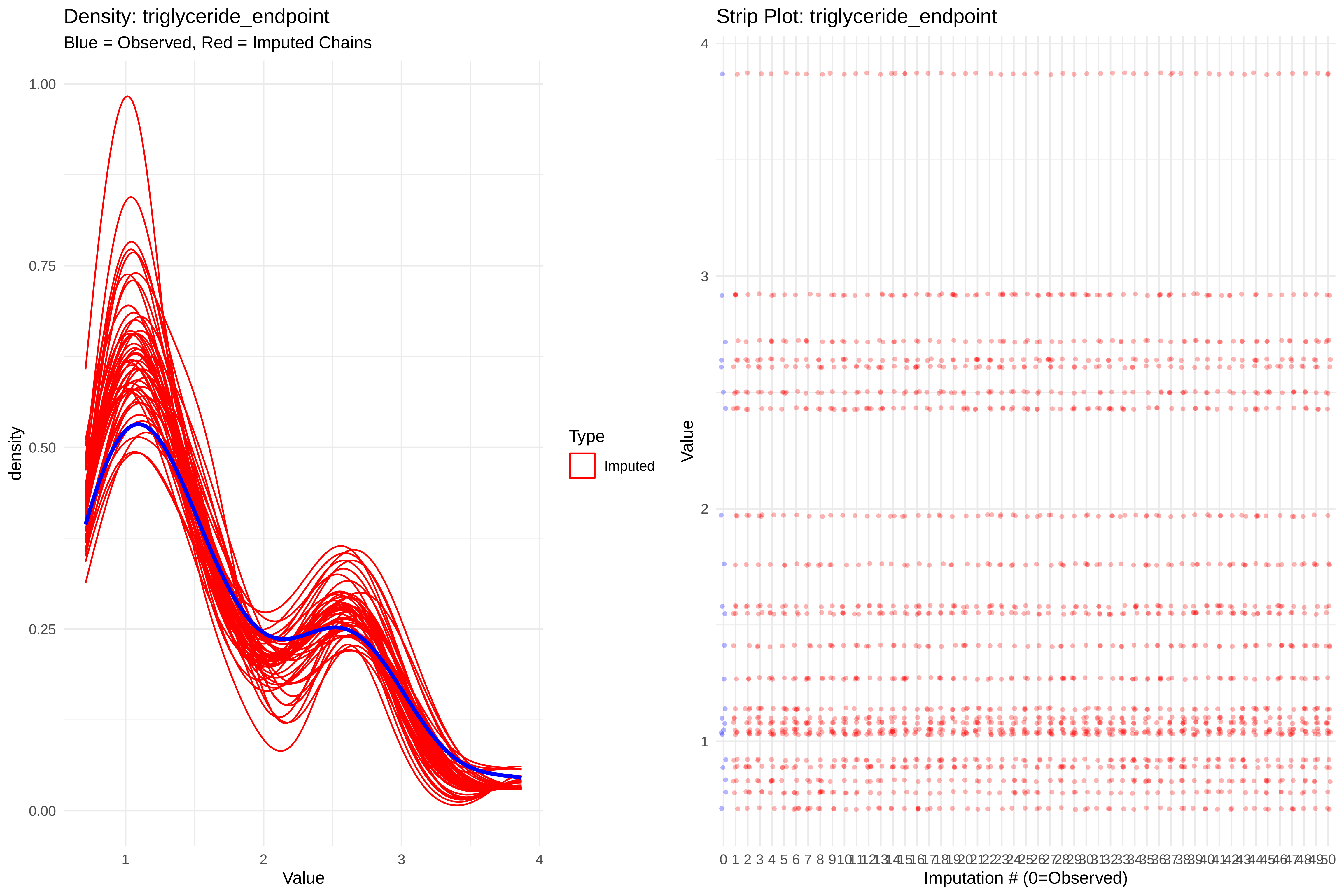
Density: triglyceride_endpoint
Blue = Observed, Red = Imputed Chains
density
1.00
0.75
0.50
0.25
0.00
1
2
3
4
Value
Type
Imputed
Strip Plot: triglyceride_endpoint
Value
4
3
2
1
Imputation # (0=Observed)

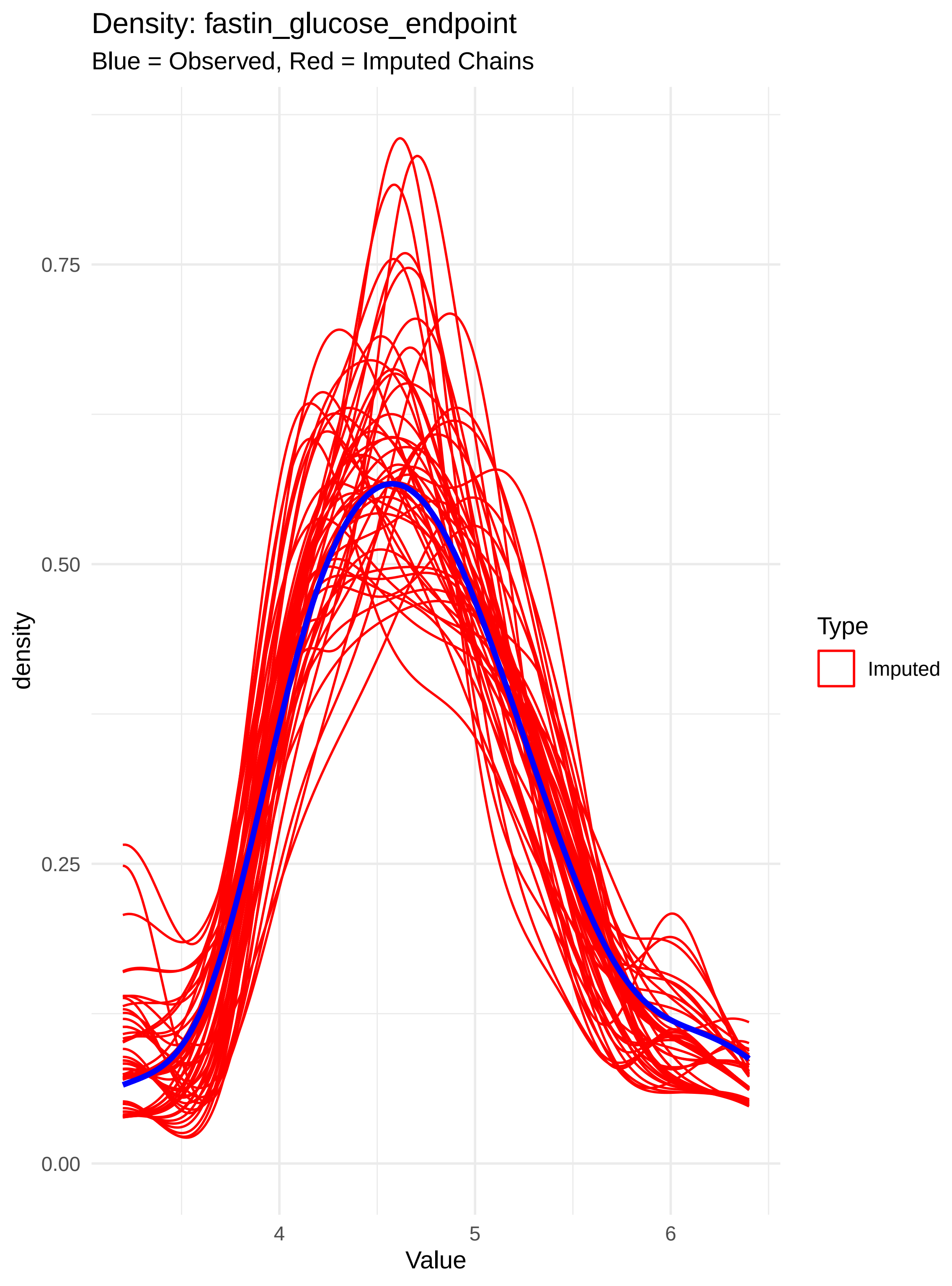
Density: fastin_glucose_endpoint
Blue = Observed, Red = Imputed Chains
density
0.00
0.25
0.50
0.75
4
5
6
Value
Type
Imputed

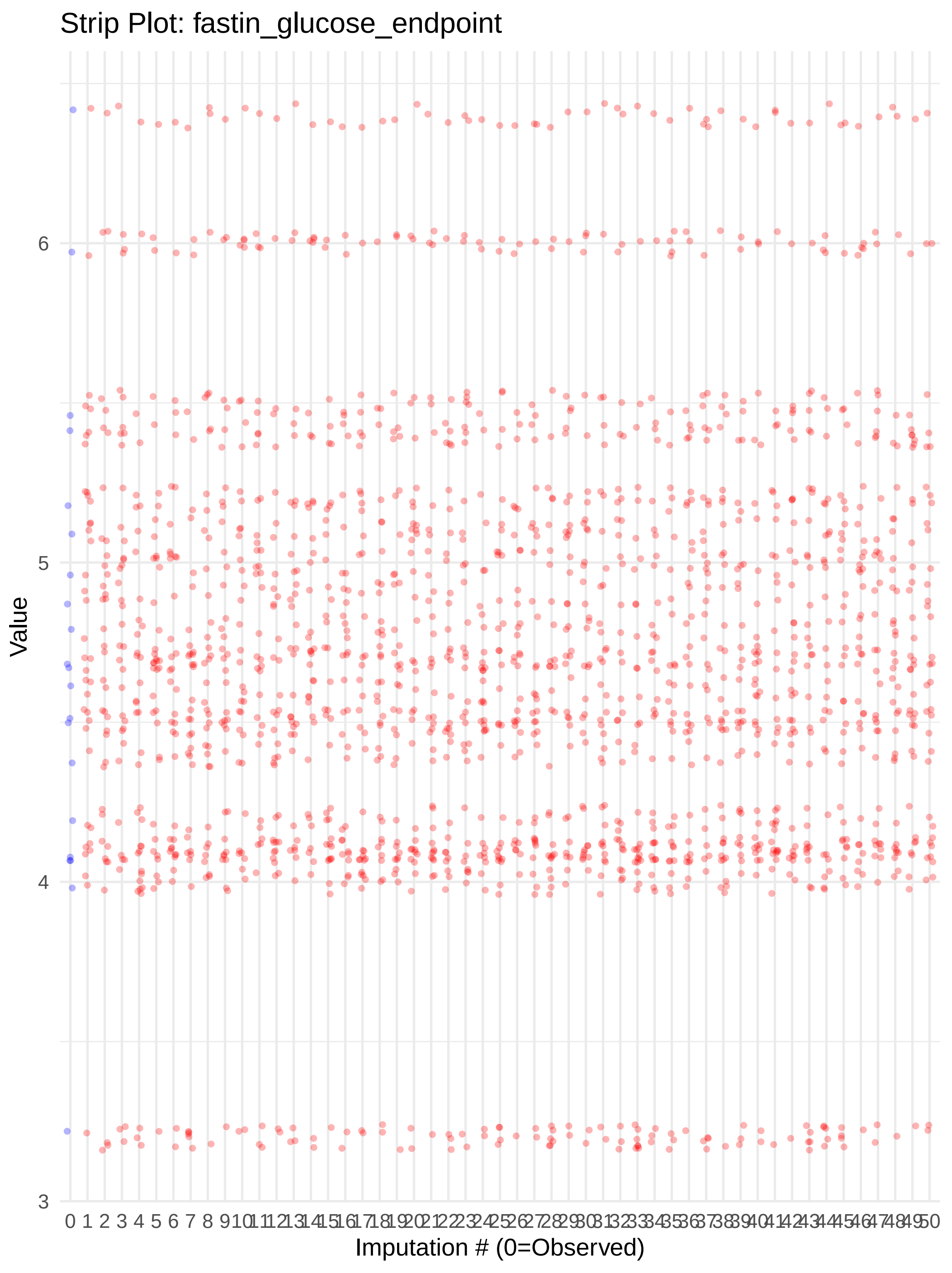
Strip Plot: fastin_glucose_endpoint
Value
3
4
5
6
Imputation # (0=Observed)

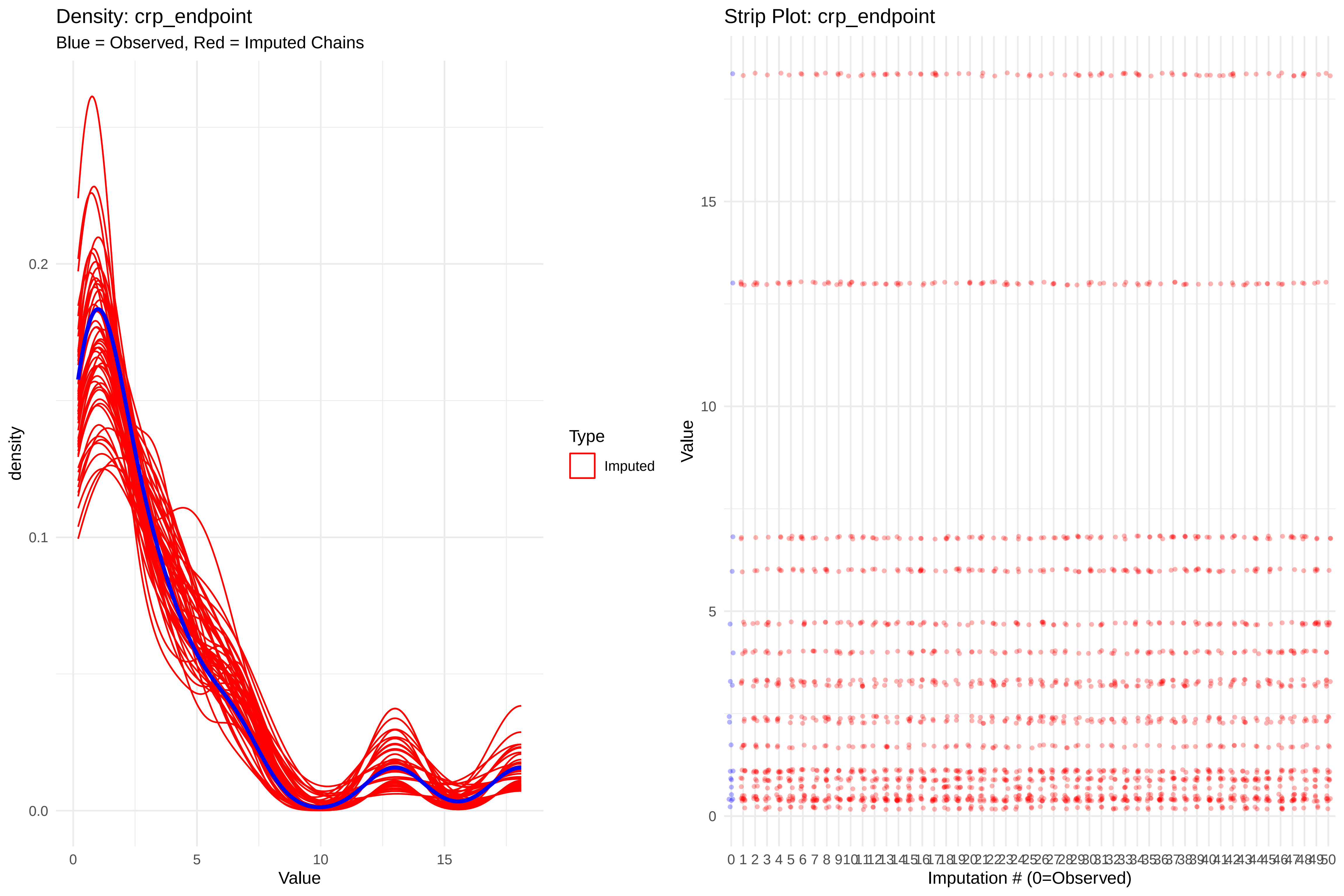

Density: crp_endpoint
Blue = Observed, Red = Imputed Chains
density
Value
Type
Imputed
Strip Plot: crp_endpoint
Value
Imputation # (0=Observed)

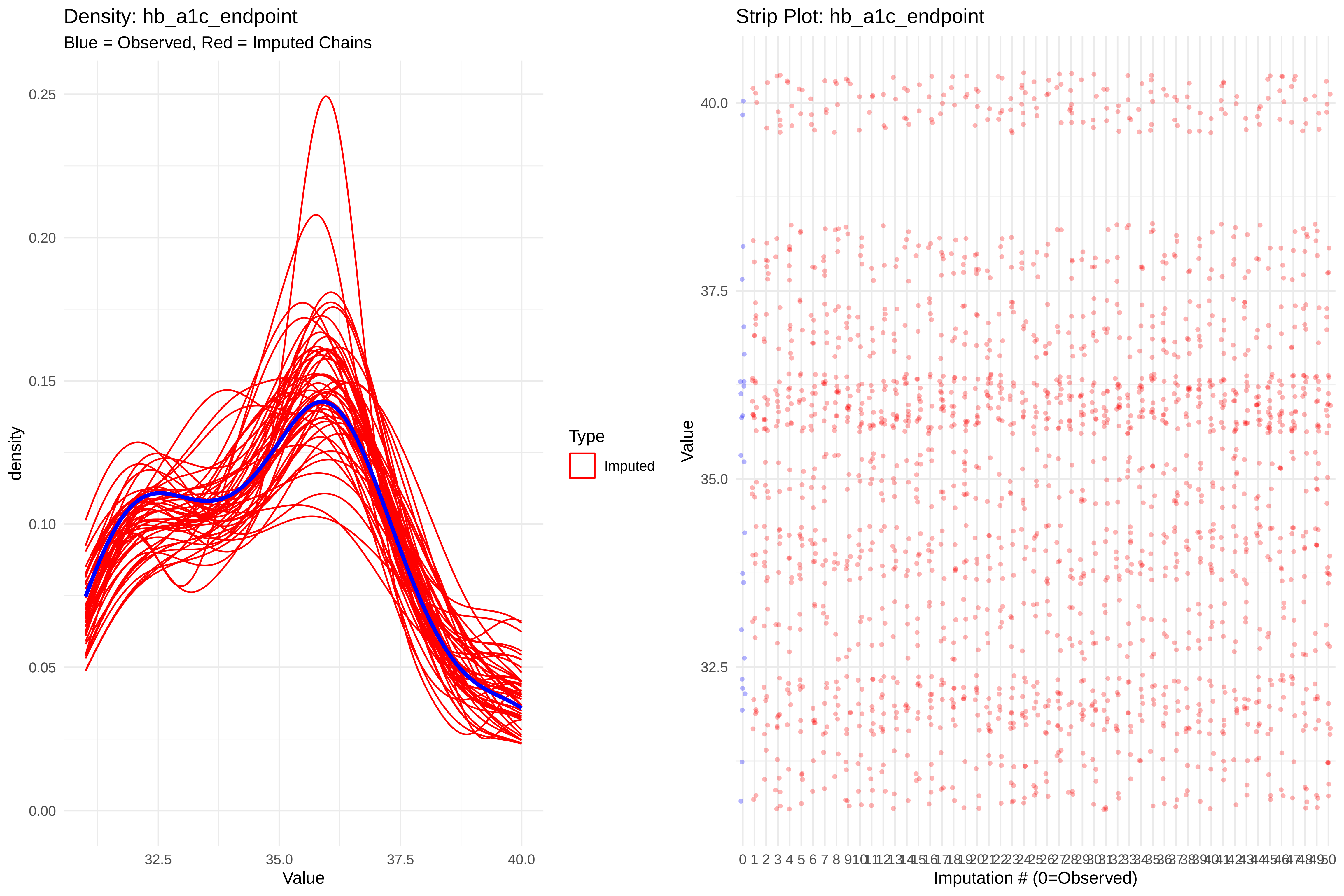

Density: hb_a1c_endpoint
Blue = Observed, Red = Imputed Chains
density
0.25
0.20
0.15
0.10
0.05
0.00
32.5
35.0
37.5
40.0
Value
Type
Imputed
Strip Plot: hb_a1c_endpoint
Value
40.0
37.5
35.0
32.5
Imputation # (0=Observed)

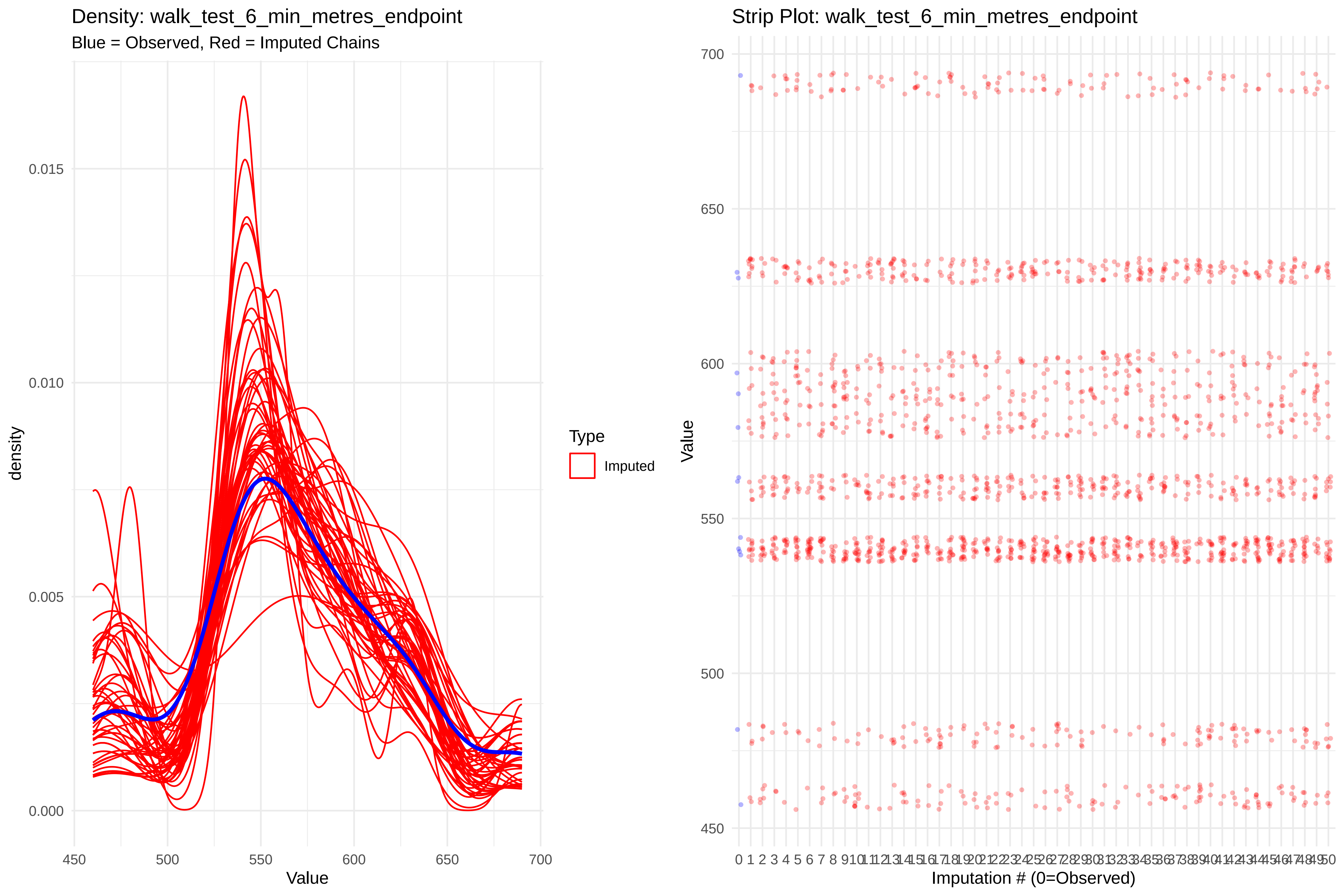
Density: walk_test_6_min_metres_endpoint
Blue = Observed, Red = Imputed Chains
density
0.015
0.010
0.005
0.000
450
500
550
600
650
700
Value
Type
Imputed
Strip Plot: walk_test_6_min_metres_endpoint
Value
700
650
600
550
500
450
0 1 2 3 4 5 6 7 8 9 10 11 12 13 14 15 16 17 18 19 20 21 22 23 24 25 26 27 28 29 30 31 32 33 34 35 36 37 38 39 40 41 42 43 44 45 46 47 48 49 50
Imputation # (0=Observed)

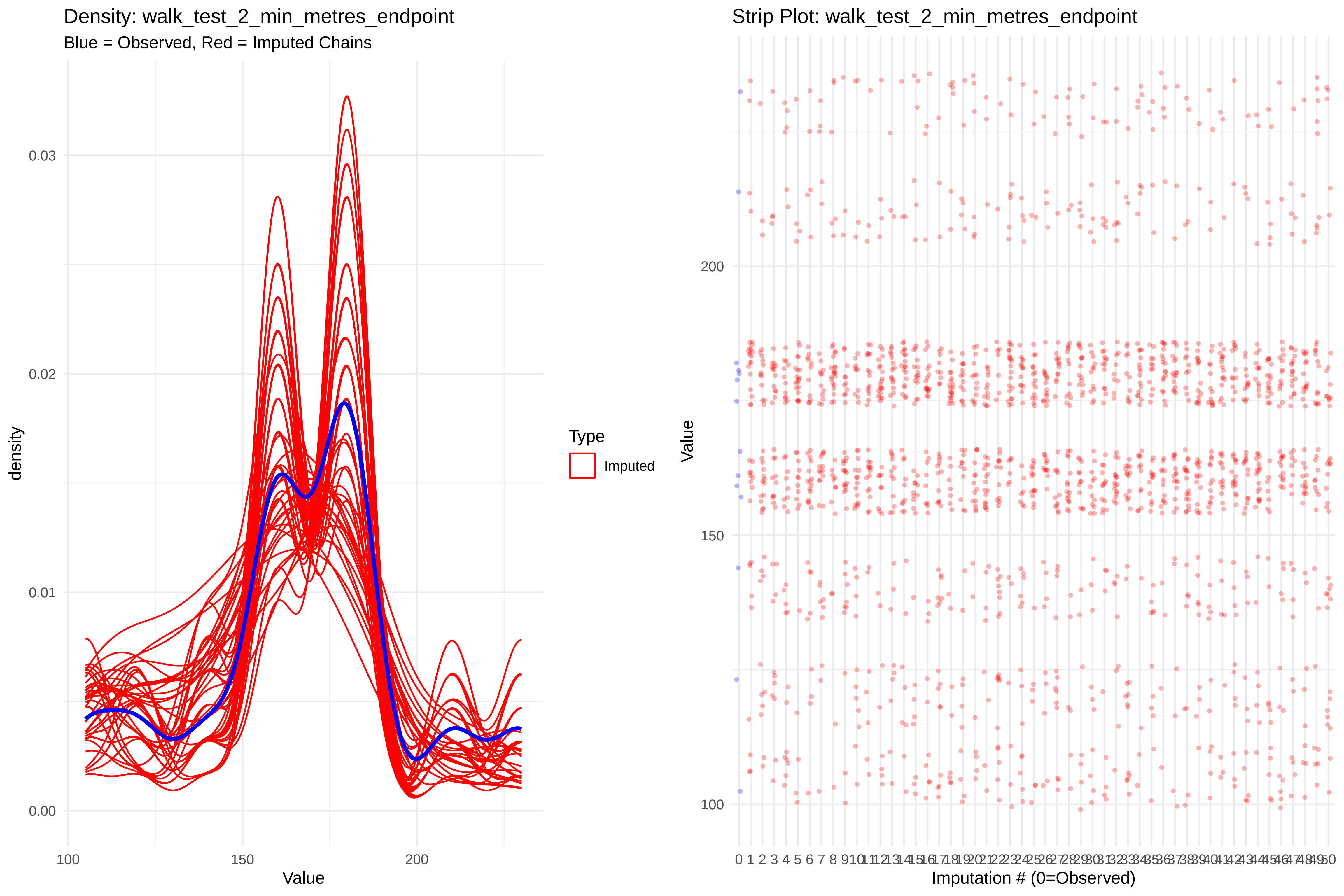
Density: walk_test_2_min_metres_endpoint
Blue = Observed, Red = Imputed Chains
density
0.00
0.01
0.02
0.03
100
150
200
Value
Type
Imputed
Strip Plot: walk_test_2_min_metres_endpoint
Value
100
150
200
Imputation # (0=Observed)

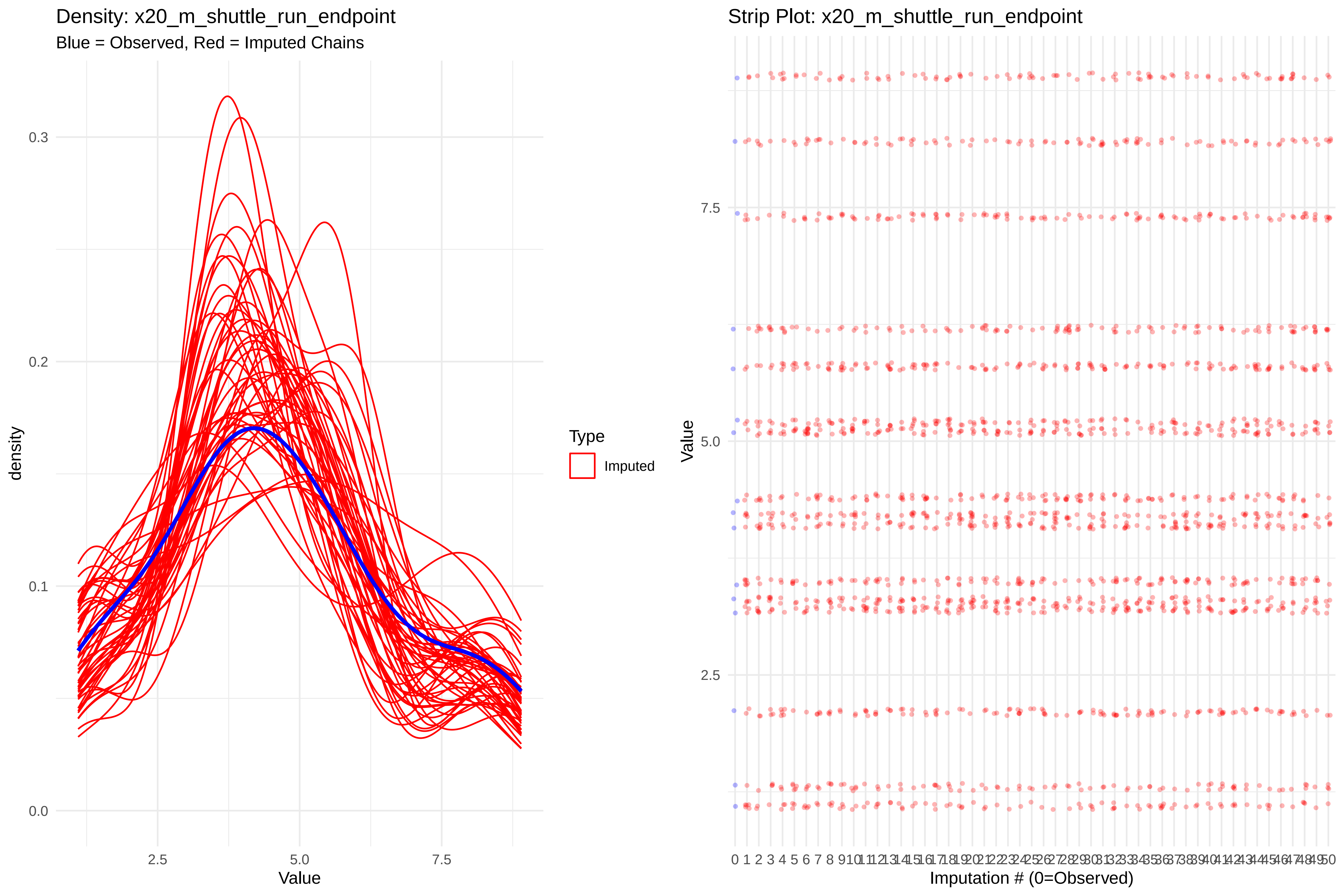

Density: x20_m_shuttle_run_endpoint
Blue = Observed, Red = Imputed Chains
density
Value
Type
Imputed
Strip Plot: x20_m_shuttle_run_endpoint
Value
Imputation # (0=Observed)

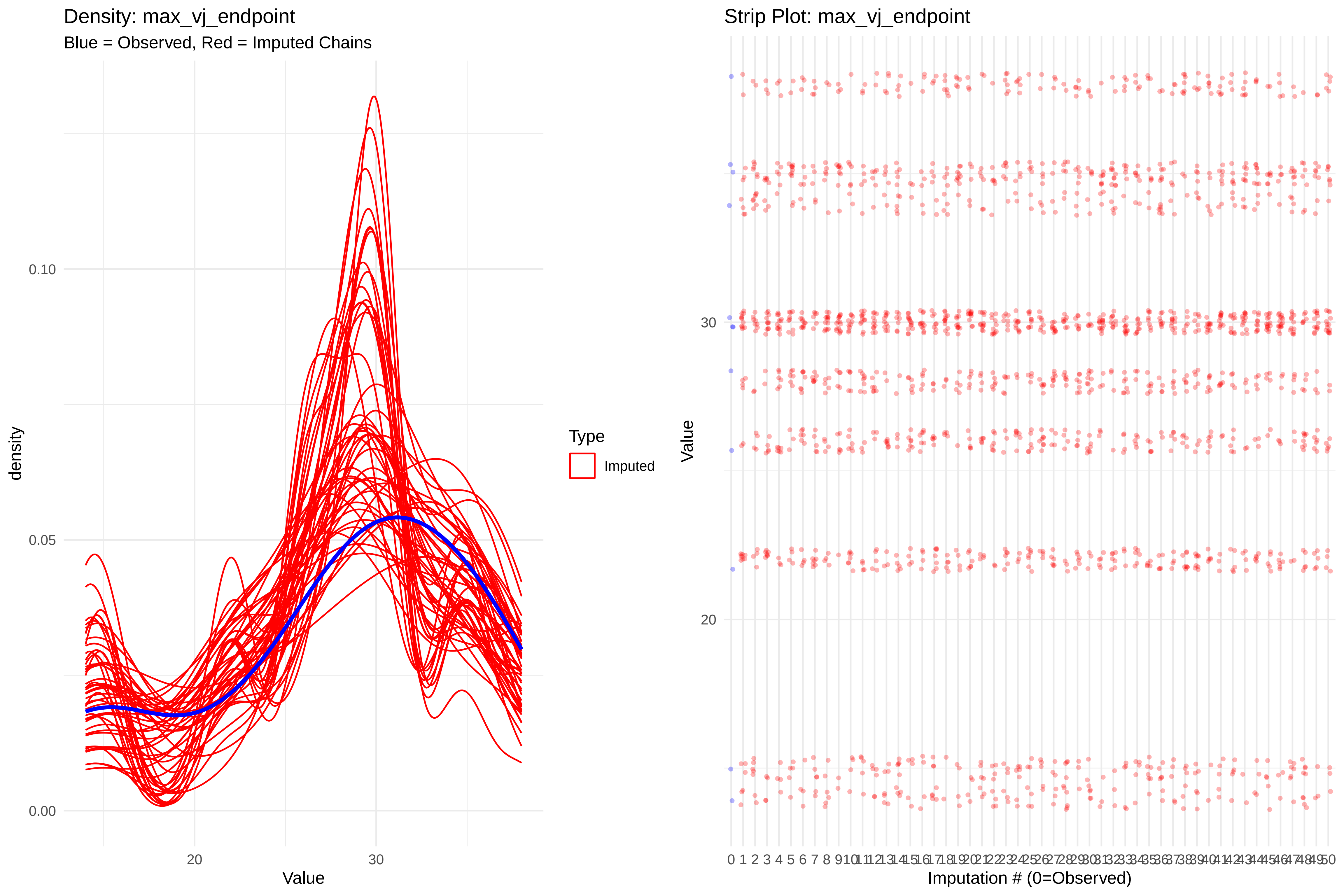

Density: max_vj_endpoint
Blue = Observed, Red = Imputed Chains
density
Value
0.00
0.05
0.10
20
30
Type
Imputed
Strip Plot: max_vj_endpoint
Value
20
30
Imputation # (0=Observed)

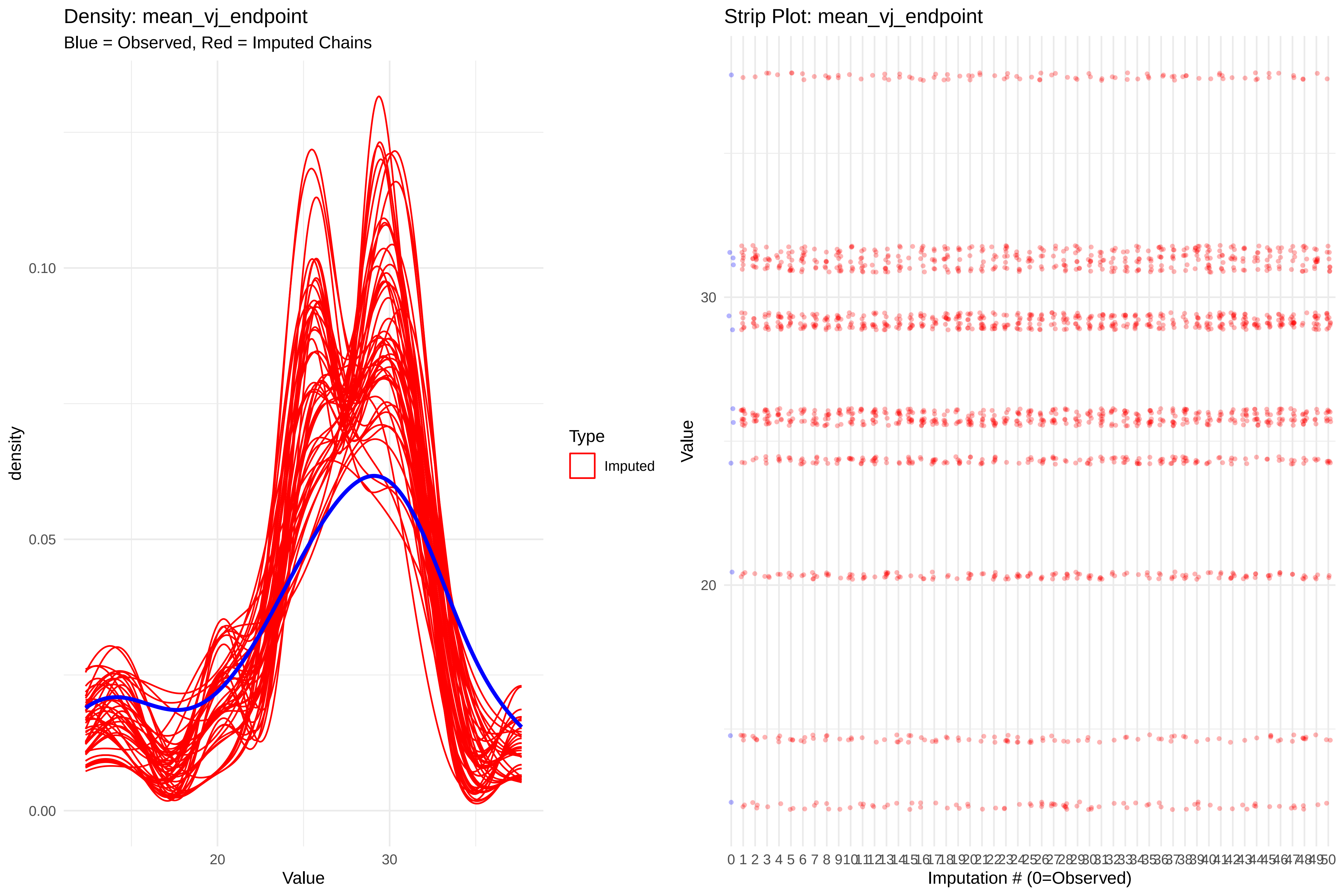

Density: mean_vj_endpoint
Blue = Observed, Red = Imputed Chains
density
Value
0.00
0.05
0.10
20
30
Type
Imputed
Strip Plot: mean_vj_endpoint
Value
20
30
Imputation # (0=Observed)

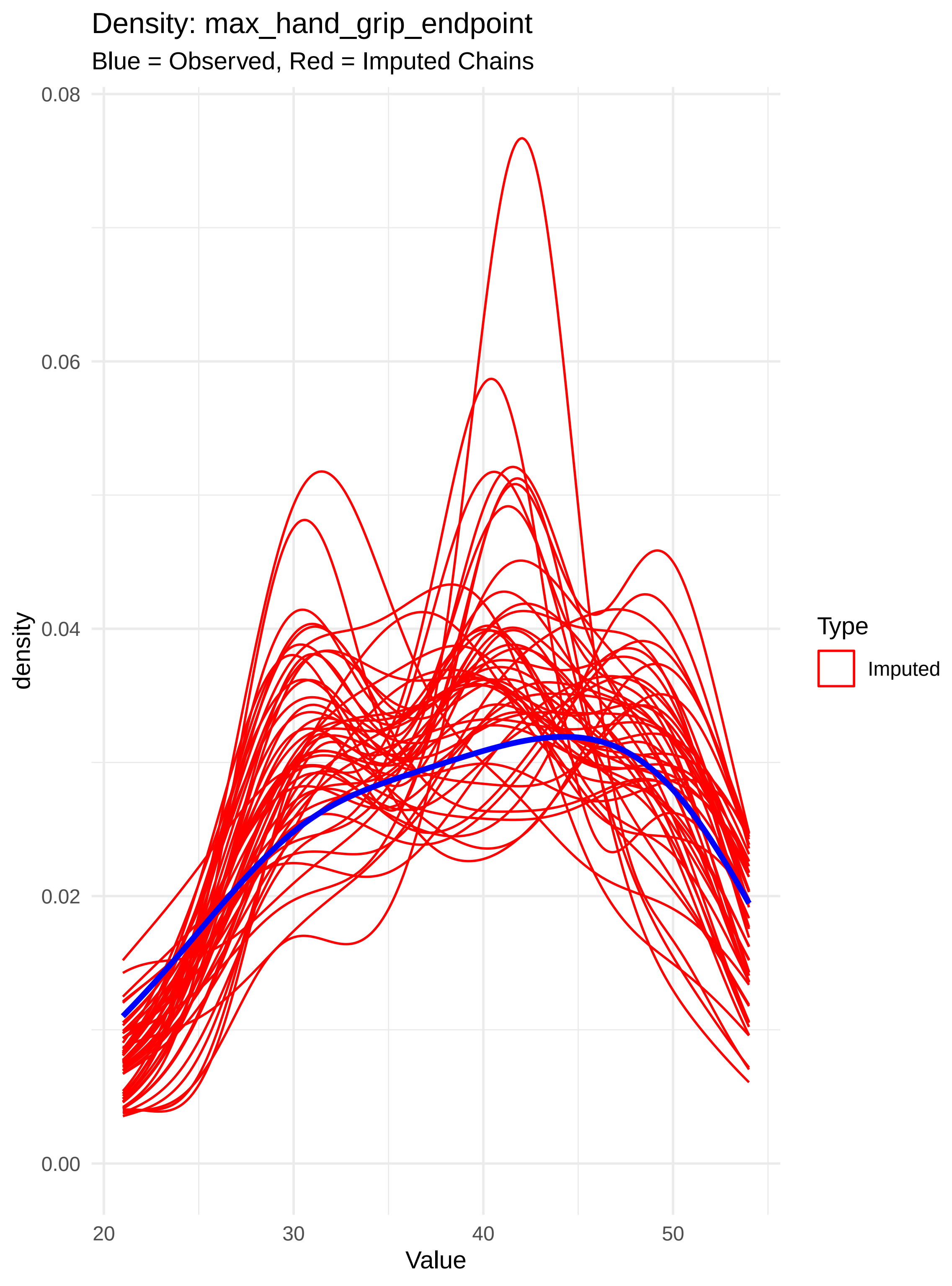

Density: max_hand_grip_endpoint
Blue = Observed, Red = Imputed Chains
density
Value
Type
Imputed
0.00
0.02
0.04
0.06
0.08
20
30
40
50

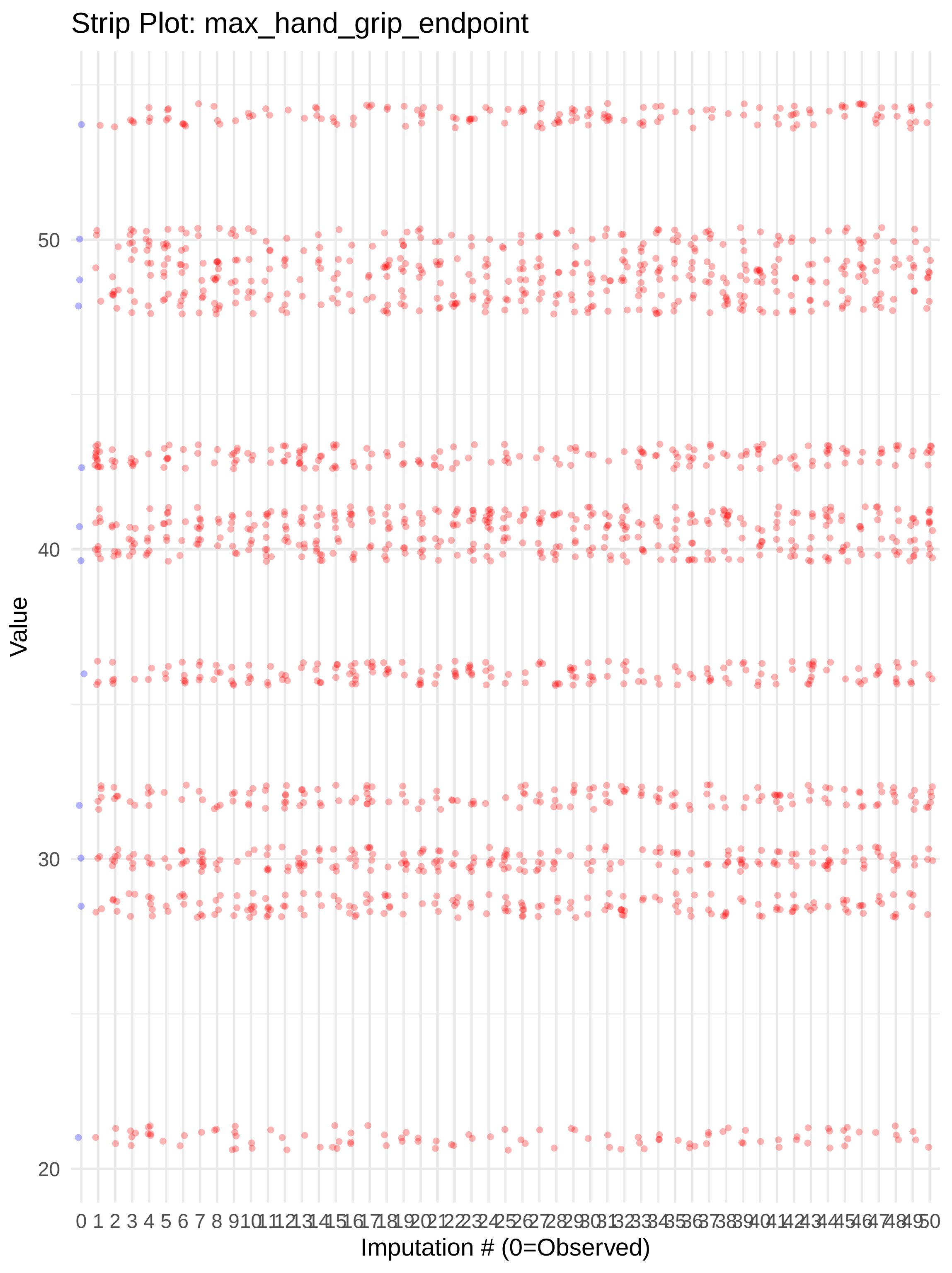

Strip Plot: max_hand_grip_endpoint
Value
Imputation # (0=Observed)
20
30
40
50

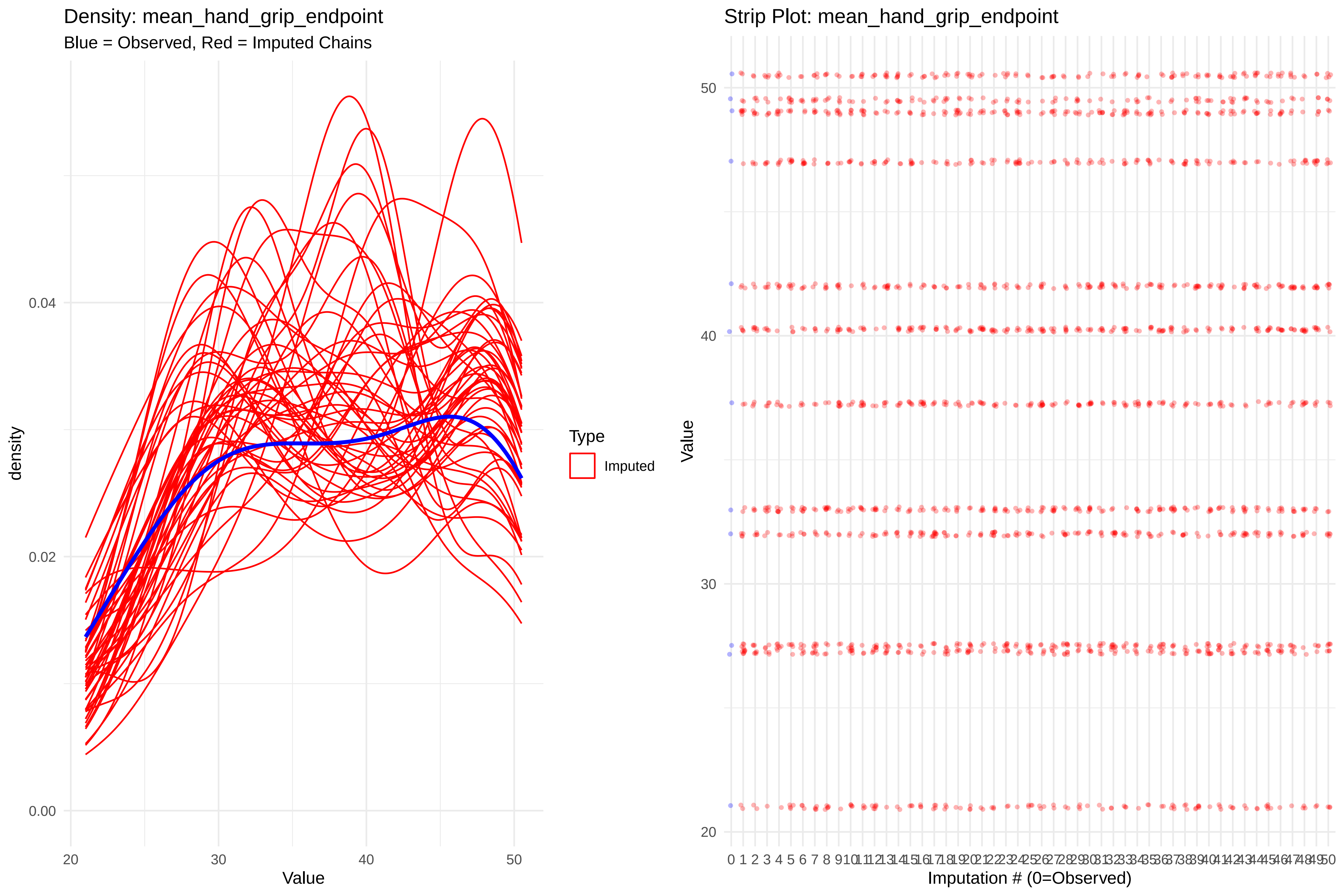
Density: mean_hand_grip_endpoint
Blue = Observed, Red = Imputed Chains
density
0.00
0.02
0.04
20
30
40
50
Value
Type
Imputed
Strip Plot: mean_hand_grip_endpoint
Value
20
30
40
50
Imputation # (0=Observed)
