# Supplementary material for: A Randomised Feasibility Trial of a Service‐Coordinated Exercise Intervention in First‐Episode Psychosis: Challenges in Implementation and Outcome Assessment
Source: Early Interv Psychiatry. 2026 Jul 9;20(7):e70193. doi: 10.1111/eip.70193 (PMC13349341; doi:10.1111/eip.70193)
Supplement: Supplementary file 3 — Table S1: Results of the Intention‐to‐treat analysis with imputed data. [file EIP-20-0-s001.docx]

# Supplementary files

## Full results – Intention to treat analysis

|  |  | Baseline | Endpoint |  | Baseline | Endpoint |  |  |
| --- | --- | --- | --- | --- | --- | --- | --- | --- |
|  | **Control** | | | **Intervention** | | | **Analysis** | |
| **Outcome Measure** | N | Mean (SD) | Mean (SD) | N | Mean (SD) | Mean (SD) | **Adj. Diff [95% CI]** | ***p*-value** |
| **Physical Health Outcomes** | | | | | | | | |
| Body Mass Index (kg/m²) | 12 | 28.3 (3.5) | 28.5 (4.0) | 14 | 27.3 (5.1) | 27.4 (5.1) | -0.13 [-1.13, 0.86] | 0.783 |
| Waist Circumference (cm) | 13 | 98.0 (11.7) | 96.9 (10.0) | 13 | 94.3 (15.0) | 93.8 (14.0) | -0.06 [-3.49, 3.38] | 0.973 |
| Hip Circumference (cm) | 12 | 106.2 (7.0) | 104.9 (7.6) | 12 | 103.9 (7.8) | 102.9 (9.9) | -4.33 [-15.35, 6.69] | 0.424 |
| Systolic BP (mmHg) | 7 | 120.7 (7.0) | 114.1 (8.4) | 11 | 113.0 (12.5) | 110.5 (9.7) | -1.81 [-11.92, 8.29] | 0.708 |
| IPAQ Activity Score | 8 | 2,805.0 (3,902.8) | 2,727.4 (4,168.3) | 13 | 2,345.8 (2,296.2) | 4,491.8 (4,082.0) | 2,039.73 [-1,562.78, 5,642.23] | 0.250 |
| 6 Minute Walk (m) | 7 | 554.3 (51.3) | 557.1 (53.1) | 5 | 568.0 (59.3) | 562.0 (57.6) | -2.40 [-68.27, 63.46] | 0.936 |
| 2 Minute Walk (m) | 7 | 182.9 (13.8) | 160.7 (28.9) | 6 | 190.0 (16.7) | 165.0 (29.5) | 1.24 [-36.94, 39.41] | 0.944 |
| 20m Shuttle Run (Level) | 7 | 4.4 (2.2) | 4.1 (2.5) | 6 | 4.6 (1.4) | 5.8 (2.3) | 1.52 [0.12, 2.93] | 0.036 |
| Max Vertical Jump (cm) | 6 | 25.7 (7.7) | 25.2 (9.2) | 5 | 33.6 (8.2) | 32.8 (2.6) | 3.07 [-6.10, 12.24] | 0.463 |
| Mean Vertical Jump (cm) | 6 | 22.9 (9.7) | 24.0 (9.4) | 5 | 30.9 (6.7) | 29.8 (2.4) | 0.79 [-7.24, 8.83] | 0.825 |
| Max Hand Grip (kg) | 6 | 33.1 (9.2) | 36.7 (11.0) | 5 | 39.5 (12.2) | 42.3 (10.3) | 0.22 [-8.84, 9.29] | 0.956 |
| Mean Hand Grip (kg) | 6 | 32.1 (9.2) | 35.6 (11.0) | 5 | 37.4 (11.5) | 40.5 (10.2) | 0.34 [-8.92, 9.59] | 0.935 |
| **Psychometric Outcomes** | | | | | | | | |
| STAI Total Score | 10 | 74.9 (32.5) | 74.9 (35.1) | 14 | 95.8 (26.9) | 87.3 (24.1) | 10.10 [-17.06, 37.27] | 0.448 |
| STAI State | 9 | 36.9 (9.0) | 37.8 (14.2) | 14 | 43.6 (15.1) | 40.0 (11.2) | -0.72 [-11.14, 9.69] | 0.886 |
| STAI Trait | 7 | 45.6 (15.5) | 39.1 (13.0) | 14 | 52.1 (14.8) | 47.3 (15.2) | 3.73 [-6.96, 14.43] | 0.473 |
| Perceived Stress (PSS) | 9 | 16.1 (8.0) | 13.4 (5.6) | 17 | 18.5 (6.8) | 15.7 (8.6) | 0.38 [-4.27, 5.02] | 0.868 |
| Social Interaction Anxiety | 9 | 21.7 (15.8) | 21.8 (16.7) | 16 | 38.9 (22.9) | 26.5 (21.0) | -8.34 [-19.65, 2.97] | 0.140 |
| Depression (CDSS) | 11 | 6.2 (5.8) | 6.2 (6.9) | 15 | 7.3 (6.3) | 6.6 (4.8) | -0.11 [-4.37, 4.14] | 0.957 |
| SANS/SAPS Combined | 11 | 7.8 (6.3) | 7.1 (6.1) | 12 | 6.8 (5.6) | 4.6 (3.6) | -2.25 [-6.43, 1.93] | 0.274 |
| EQ-5D-5L Index | 10 | 0.8 (0.2) | 0.8 (0.2) | 13 | 0.7 (0.4) | 0.8 (0.3) | 0.08 [0.01, 0.16] | 0.031 |
| EQ-VAS | 10 | 63.5 (22.6) | 66.0 (22.9) | 13 | 55.1 (22.7) | 71.4 (23.8) | 12.06 [-1.69, 25.81] | 0.082 |
| Global Functioning (GAF) | 10 | 62.1 (11.1) | 60.6 (13.1) | 12 | 56.8 (6.8) | 60.8 (13.3) | 5.62 [-3.51, 14.74] | 0.213 |
| **Biomarkers** | | | | | | | | |
| Total Cholesterol (mmol/L) | 12 | 5.7 (1.0) | 5.8 (1.0) | 9 | 5.9 (1.2) | 5.8 (1.3) | -0.16 [-0.87, 0.54] | 0.629 |
| Triglycerides (mmol/L) | 12 | 1.7 (1.0) | 1.9 (1.0) | 9 | 1.6 (0.7) | 1.4 (0.7) | -0.42 [-1.09, 0.25] | 0.204 |
| Fasting Glucose (mmol/L) | 9 | 4.8 (0.8) | 4.9 (0.6) | 7 | 4.5 (0.2) | 4.3 (0.7) | -0.52 [-1.20, 0.16] | 0.120 |
| C-Reactive Protein (mg/L) | 11 | 2.2 (1.8) | 3.5 (3.7) | 8 | 2.2 (2.0) | 1.9 (1.9) | -1.62 [-4.32, 1.08] | 0.221 |
| HbA1c (mmol/mol) | 12 | 35.6 (3.2) | 35.2 (2.9) | 10 | 37.1 (3.6) | 35.0 (2.4) | -0.82 [-2.95, 1.31] | 0.431 |
| **CANTAB Cognitive Outcomes** | | | | | | | | |
| VRM: Free Recall (Correct) | 6 | 6.3 (2.5) | 5.3 (1.4) | 10 | 4.8 (1.5) | 5.0 (2.4) | -0.50 [-3.10, 2.11] | 0.687 |
| VRM: Immediate Recog (Correct) | 6 | 33.2 (2.6) | 30.8 (3.8) | 10 | 31.2 (5.5) | 28.4 (5.0) | -1.13 [-5.16, 2.89] | 0.553 |
| VRM: Delayed Recog (Correct) | 6 | 31.0 (6.2) | 31.2 (3.4) | 8 | 31.2 (3.4) | 28.9 (2.4) | -2.38 [-5.24, 0.48] | 0.095 |
| RTI: Simple Reaction Time (ms) | 5 | 351.6 (36.3) | 427.5 (125.2) | 5 | 358.1 (81.2) | 415.0 (86.1) | -19.64 [-151.53, 112.26] | 0.735 |
| RTI: 5-Choice Reaction Time (ms) | 5 | 425.0 (45.5) | 462.7 (92.9) | 5 | 434.5 (102.1) | 447.7 (96.7) | -25.18 [-92.93, 42.58] | 0.409 |
| OTS: Problems Solved (1st Choice) | 5 | 10.0 (1.9) | 9.2 (1.9) | 4 | 8.2 (4.0) | 11.2 (2.8) | 2.96 [-0.23, 6.15] | 0.063 |
| OTS: Mean Latency (ms) | 5 | 23,089.0 (19,445.0) | 17,839.5 (13,744.8) | 4 | 16,200.2 (10,530.8) | 15,608.3 (7,776.1) | 2,301.93 [-5,794.67, 10,398.53] | 0.513 |
| ERT: Emotion Recog (Total Hits) | 3 | 27.7 (5.9) | 30.3 (2.3) | 2 | 26.0 (1.4) | 25.5 (0.7) | -4.59 [-13.42, 4.24] | 0.155 |
| Note. N reflects participants with valid data at both baseline and endpoint. Adjusted Difference = Intervention - Control. | | | | | | | | |

## Per-Protocol Results

|  |  | Baseline | Endpoint |  | Baseline | Endpoint |  |  |
| --- | --- | --- | --- | --- | --- | --- | --- | --- |
|  | **Control Group** | | | **Intervention Group** | | | **Analysis** | |
| **Outcome Measure** | N | Mean (SD) | Mean (SD) | N | Mean (SD) | Mean (SD) | **Adj. Diff [95% CI]** | ***p*-value** |
| **Physical Health Outcomes** | | | | | | | | |
| Body Mass Index (kg/m²) | 12 | 28.3 (3.5) | 28.5 (4.0) | 12 | 27.4 (4.7) | 27.4 (4.6) | -0.22 [-1.28, 0.85] | 0.676 |
| Waist Circumference (cm) | 13 | 98.0 (11.7) | 96.9 (10.0) | 11 | 94.6 (15.1) | 94.1 (14.1) | 0.05 [-3.71, 3.81] | 0.979 |
| Hip Circumference (cm) | 13 | 106.2 (7.0) | 104.9 (7.6) | 10 | 103.1 (5.5) | 101.7 (8.3) | -4.47 [-16.89, 7.95] | 0.461 |
| Systolic BP (mmHg) | 7 | 120.7 (7.0) | 114.1 (8.4) | 10 | 112.3 (13.0) | 110.6 (10.3) | -1.52 [-12.34, 9.30] | 0.768 |
| IPAQ Activity Score | 8 | 2,805.0 (3,902.8) | 2,727.4 (4,168.3) | 9 | 2,318.8 (2,248.7) | 3,457.4 (2,514.7) | 1,184.21 [-667.69, 3,036.11] | 0.192 |
| 6 Minute Walk (m) | 7 | 554.3 (51.3) | 557.1 (53.1) | 5 | 568.0 (59.3) | 562.0 (57.6) | -2.40 [-68.27, 63.46] | 0.936 |
| 2 Minute Walk (m) | 7 | 182.9 (13.8) | 160.7 (28.9) | 6 | 190.0 (16.7) | 165.0 (29.5) | 1.24 [-36.94, 39.41] | 0.944 |
| 20m Shuttle Run (Level) | 7 | 4.4 (2.2) | 4.1 (2.5) | 6 | 4.6 (1.4) | 5.8 (2.3) | 1.52 [0.12, 2.93] | 0.036 |
| Max Vertical Jump (cm) | 6 | 25.7 (7.7) | 25.2 (9.2) | 5 | 33.6 (8.2) | 32.8 (2.6) | 3.07 [-6.10, 12.24] | 0.463 |
| Mean Vertical Jump (cm) | 6 | 22.9 (9.7) | 24.0 (9.4) | 5 | 30.9 (6.7) | 29.8 (2.4) | 0.79 [-7.24, 8.83] | 0.825 |
| Max Hand Grip (kg) | 6 | 33.1 (9.2) | 36.7 (11.0) | 5 | 39.5 (12.2) | 42.3 (10.3) | 0.22 [-8.84, 9.29] | 0.956 |
| Mean Hand Grip (kg) | 6 | 32.1 (9.2) | 35.6 (11.0) | 5 | 37.4 (11.5) | 40.5 (10.2) | 0.34 [-8.92, 9.59] | 0.935 |
| **Psychometric Outcomes** | | | | | | | | |
| STAI Total Score | 10 | 74.9 (32.5) | 74.9 (35.1) | 11 | 103.6 (22.6) | 91.7 (18.3) | 19.76 [-9.69, 49.20] | 0.176 |
| STAI State | 9 | 36.9 (9.0) | 37.8 (14.2) | 11 | 46.9 (15.0) | 41.5 (9.6) | 0.13 [-11.41, 11.67] | 0.981 |
| STAI Trait | 7 | 45.6 (15.5) | 39.1 (13.0) | 11 | 56.7 (11.6) | 50.2 (12.4) | 4.86 [-7.06, 16.78] | 0.399 |
| Perceived Stress (PSS) | 9 | 16.1 (8.0) | 13.4 (5.6) | 12 | 20.4 (5.2) | 17.1 (8.7) | 0.46 [-5.39, 6.30] | 0.872 |
| Social Interaction Anxiety | 9 | 21.7 (15.8) | 21.8 (16.7) | 12 | 42.6 (23.5) | 30.4 (21.6) | -6.63 [-20.25, 7.00] | 0.320 |
| Depression (CDSS) | 11 | 6.2 (5.8) | 6.2 (6.9) | 12 | 8.3 (6.3) | 7.0 (5.0) | -0.17 [-4.98, 4.64] | 0.943 |
| SANS/SAPS Combined | 11 | 7.8 (6.3) | 7.1 (6.1) | 10 | 8.2 (5.1) | 5.1 (3.6) | -2.08 [-6.66, 2.50] | 0.352 |
| EQ-5D-5L Index | 10 | 0.8 (0.2) | 0.8 (0.2) | 10 | 0.6 (0.5) | 0.7 (0.4) | 0.09 [0.00, 0.18] | 0.044 |
| EQ-VAS | 10 | 63.5 (22.6) | 66.0 (22.9) | 10 | 55.7 (22.3) | 68.3 (23.2) | 8.40 [-6.39, 23.18] | 0.247 |
| Global Functioning (GAF) | 10 | 62.1 (11.1) | 60.6 (13.1) | 10 | 55.2 (5.8) | 58.0 (10.6) | 3.41 [-6.15, 12.97] | 0.462 |
| **Biomarkers** | | | | | | | | |
| Total Cholesterol (mmol/L) | 12 | 5.7 (1.0) | 5.8 (1.0) | 8 | 6.0 (1.2) | 6.0 (1.3) | -0.10 [-0.84, 0.65] | 0.790 |
| Triglycerides (mmol/L) | 12 | 1.7 (1.0) | 1.9 (1.0) | 8 | 1.6 (0.8) | 1.5 (0.8) | -0.42 [-1.14, 0.30] | 0.231 |
| Fasting Glucose (mmol/L) | 9 | 4.8 (0.8) | 4.9 (0.6) | 6 | 4.6 (0.2) | 4.3 (0.8) | -0.52 [-1.26, 0.21] | 0.146 |
| C-Reactive Protein (mg/L) | 11 | 2.2 (1.8) | 3.5 (3.7) | 7 | 1.6 (0.9) | 1.7 (2.0) | -1.07 [-3.91, 1.76] | 0.432 |
| HbA1c (mmol/mol) | 12 | 35.6 (3.2) | 35.2 (2.9) | 9 | 37.6 (3.5) | 35.1 (2.5) | -0.94 [-3.23, 1.36] | 0.403 |
| **CANTAB Cognitive Outcomes** | | | | | | | | |
| VRM: Free Recall (Correct) | 6 | 6.3 (2.5) | 5.3 (1.4) | 8 | 4.6 (1.1) | 4.1 (1.1) | -1.18 [-2.89, 0.54] | 0.158 |
| VRM: Immediate Recog (Correct) | 6 | 33.2 (2.6) | 30.8 (3.8) | 8 | 30.6 (6.1) | 27.8 (5.4) | -1.46 [-6.13, 3.20] | 0.504 |
| VRM: Delayed Recog (Correct) | 6 | 31.0 (6.2) | 31.2 (3.4) | 7 | 30.7 (3.3) | 28.7 (2.5) | -2.35 [-5.48, 0.78] | 0.125 |
| RTI: Simple Reaction Time (ms) | 5 | 351.6 (36.3) | 427.5 (125.2) | 4 | 360.6 (93.5) | 429.3 (92.3) | -7.88 [-160.49, 144.73] | 0.904 |
| RTI: 5-Choice Reaction Time (ms) | 5 | 425.0 (45.5) | 462.7 (92.9) | 4 | 442.4 (116.1) | 451.0 (111.4) | -30.69 [-109.79, 48.41] | 0.379 |
| OTS: Problems Solved (1st Choice) | 5 | 10.0 (1.9) | 9.2 (1.9) | 3 | 9.0 (4.6) | 12.3 (2.1) | 3.54 [0.35, 6.74] | 0.036 |
| OTS: Mean Latency (ms) | 5 | 23,089.0 (19,445.0) | 17,839.5 (13,744.8) | 3 | 21,014.3 (5,224.4) | 18,833.9 (5,317.3) | 2,355.25 [-7,534.20, 12,244.69] | 0.567 |
| ERT: Emotion Recog (Total Hits) | 3 | 27.7 (5.9) | 30.3 (2.3) | 2 | 26.0 (1.4) | 25.5 (0.7) | -4.59 [-13.42, 4.24] | 0.155 |
| *Note.* Data presented as Mean (SD). Difference = Intervention minus Control (adjusted for baseline value). N reflects participants with valid data at both timepoints (Complete Case). | | | | | | | | |

## Intention to treat analysis with imputed data

|  | | | | |
| --- | --- | --- | --- | --- |
|  | | | | |
|  |  |  |  |  |
|  |  |  |  |  |
|  |  |  |  |  |
|  |  |  |  |  |
|  |  |  |  |  |
|  |  |  |  |  |
|  |  |  |  |  |
|  |  |  |  |  |
|  |  |  |  |  |
|  |  |  |  |  |
|  |  |  |  |  |
|  |  |  |  |  |
|  |  |  |  |  |
|  |  |  |  |  |
|  |  |  |  |  |
|  |  |  |  |  |
|  |  |  |  |  |
|  |  |  |  |  |
|  |  |  |  |  |
|  |  |  |  |  |
|  |  |  |  |  |
|  |  |  |  |  |
|  |  |  |  |  |
|  |  |  |  |  |
|  |  |  |  |  |
|  |  |  |  |  |
|  |  |  |  |  |
|  | | | | |
| *Intervention Effects on Primary and Secondary Outcomes* | | | | |
| *Results from ANCOVA models (Endpoint ~ Baseline + Group) using Multiple Imputation (mITT population)* | | | | |

| Outcome Measure | N | Adjusted Difference (95% CI) | p-value |
| --- | --- | --- | --- |
| **Biomarkers** | | | |
| C-Reactive Protein | 36 | -0.90 (-3.66 to 1.85) | 0.494 |
| Cholesterol | 36 | 0.03 (-0.53 to 0.60) | 0.902 |
| Fasting Glucose | 36 | -0.17 (-0.71 to 0.37) | 0.514 |
| HbA1c | 36 | 0.18 (-1.60 to 1.95) | 0.838 |
| Triglyceride | 36 | -0.13 (-0.76 to 0.50) | 0.670 |
| **Physical Health** | | | |
| 20m Shuttle Run (Level) | 32 | 0.74 (-0.69 to 2.18) | 0.289 |
| Body Mass Index | 36 | -0.43 (-1.26 to 0.41) | 0.311 |
| Max Hand Grip | 30 | -0.11 (-8.44 to 8.22) | 0.977 |
| Vertical Jump (Max) (cm) | 29 | 2.10 (-4.96 to 9.16) | 0.531 |
| Vertical Jump (Mean) (cm) | 29 | 0.67 (-4.83 to 6.17) | 0.800 |
| Waist | 36 | -0.36 (-4.16 to 3.45) | 0.846 |
| Walk Test 2 Min Metres | 32 | 7.41 (-22.10 to 36.92) | 0.596 |
| Walk Test 6 Min Metres | 32 | 17.02 (-28.74 to 62.78) | 0.440 |
| Weight (kg) | 36 | -0.93 (-3.48 to 1.62) | 0.458 |
| **Psychometric Outcomes** | | | |
| Depression (CDSS) | 34 | -0.66 (-4.12 to 2.79) | 0.695 |
| Global Functioning (GAF) | 34 | 1.06 (-8.18 to 10.30) | 0.813 |
| Perceived Stress Scale (PSS) | 34 | 2.09 (-2.39 to 6.56) | 0.344 |
| Quality of Life (EQ-5D-5L) | 35 | 0.05 (-0.02 to 0.12) | 0.132 |
| SANS/SAPS Combined | 34 | -1.14 (-4.52 to 2.23) | 0.491 |
| STAI Total | 34 | 3.78 (-15.27 to 22.84) | 0.688 |
| Social Interaction Anxiety (SIAS) | 34 | -5.08 (-15.30 to 5.14) | 0.310 |
| State Anxiety (STAI-S) | 34 | -2.18 (-11.24 to 6.87) | 0.621 |
| Trait Anxiety (STAI-T) | 34 | 1.51 (-7.91 to 10.93) | 0.737 |
| *Note.* Adjusted Difference = Intervention minus Control. Analysis restricted to participants with a valid baseline assessment. Missing endpoints were imputed using MICE (m=50). | | | |

Supplementary Table 1: Results of the Intention-to-treat analysis with imputed data

We assessed the validity of the Multiple Imputation by Chained Equations (MICE) models through visual inspection of trace plots and kernel density estimates. The trace plots confirmed stationarity and good chain mixing across all fifty imputed datasets after 20 iterations, indicating that the algorithm successfully converged without distinct trends. The density plots demonstrated that the imputed value distributions consistently matched the observed data. The imputation models successfully preserved the distributional characteristics of the original dataset, including skewness in variables such as physical activity (IPAQ) and triglycerides, while strictly respecting logical boundaries (e.g., maintaining positive values for physical measures and being within possible scale scores).

| **Outcome** | **Group** | **Age** | **Gender** | **Weight Kg** | **BMI** | **Blood Pressure** | **Mean Hand Grip** | **Walk Test 6 Min Metres** | **X20 M Shuttle Run** | **Stai Total** | **GAF** | **CDSS** | **SANS/ SAPS** | **Eq5d5l** | **Cholesterol** | **Hb A1c** |
| --- | --- | --- | --- | --- | --- | --- | --- | --- | --- | --- | --- | --- | --- | --- | --- | --- |
| BMI | 0.726 | 0.080 | 0.127 | 0.185 | NA | 0.401 | 0.595 | 0.662 | 0.328 | 0.671 | 0.598 | 0.475 | 0.567 | 0.290 | 0.624 | 0.330 |
| Bp Sys | 0.815 | 0.938 | 0.721 | 0.963 | 0.672 | NA | 0.975 | 0.196 | 0.431 | 0.788 | 0.893 | 0.567 | 0.947 | 0.102 | 0.302 | 0.703 |
| IPAQ | 0.181 | 0.859 | 0.879 | 0.266 | 0.786 | 0.620 | 0.091 | 0.351 | 0.096 | 0.150 | 0.693 | 0.399 | 0.585 | 0.719 | 0.112 | 0.936 |
| Walk Test 6 Min Metres | 0.291 | 0.504 | 0.657 | 0.667 | 0.448 | 0.417 | 0.343 | NA | 0.481 | 0.878 | 0.653 | 0.401 | 0.575 | 0.871 | 0.337 | 0.100 |
| Walk Test 2 Min Metres | 0.291 | 0.647 | 0.849 | 0.838 | 0.275 | 0.722 | 0.343 | 0.062 | 0.585 | 0.848 | 0.972 | 0.291 | 0.823 | 0.989 | 0.648 | 0.360 |
| X20 M Shuttle Run | 0.603 | 0.718 | 0.777 | 0.292 | 0.129 | 0.622 | 0.343 | 0.101 | NA | 0.665 | 0.842 | 0.210 | 0.221 | 0.706 | 0.300 | 0.836 |
| Max Vj | 0.318 | 0.989 | 0.534 | 0.881 | 0.692 | 0.288 | 0.343 | 0.056 | 0.481 | 0.813 | 0.658 | 0.656 | 0.609 | 0.797 | 0.782 | 0.495 |
| Max Hand Grip | 0.318 | 0.989 | 0.534 | 0.881 | 0.692 | 0.288 | 0.343 | 0.056 | 0.481 | 0.813 | 0.658 | 0.656 | 0.609 | 0.797 | 0.782 | 0.495 |
| Stai Total | 0.351 | 0.796 | 0.721 | 0.653 | 0.929 | 0.421 | 0.828 | 0.714 | 0.435 | NA | 0.505 | 0.573 | 0.177 | 0.481 | 0.452 | 0.771 |
| PSS | 0.088 | 0.585 | 0.777 | 0.844 | 0.981 | 0.263 | 0.725 | 0.240 | 0.325 | 0.187 | 0.596 | 0.699 | 0.309 | 0.639 | 0.413 | 0.662 |
| Sias | 0.264 | 0.745 | 0.372 | 0.785 | 0.336 | 0.158 | 0.709 | 0.411 | 0.517 | 0.080 | 0.822 | 0.699 | 0.538 | 0.592 | 0.432 | 0.338 |
| CDSS | 0.793 | 0.170 | 0.268 | 0.845 | 0.891 | 0.380 | 0.922 | 0.726 | 0.793 | 0.266 | 0.230 | NA | 0.657 | 0.636 | 0.205 | 0.822 |
| Sans Saps | 0.991 | 0.346 | 0.895 | 0.382 | 0.419 | 0.715 | 0.398 | 0.575 | 0.432 | 0.850 | 0.975 | 0.949 | NA | 0.336 | 0.067 | 0.137 |
| Eq5d5l | 0.494 | 0.586 | 0.991 | 0.384 | 0.256 | 0.943 | 0.641 | 0.178 | 0.929 | 0.428 | 0.794 | 0.686 | 0.822 | NA | 0.018 | 0.898 |
| GAF | 0.354 | 0.029 | 0.611 | 0.184 | 0.097 | 0.860 | 0.691 | 0.355 | 0.429 | 0.522 | NA | 0.941 | 0.509 | 0.907 | 0.040 | 0.067 |
| Cholesterol | 0.475 | 0.326 | 0.338 | 0.567 | 0.920 | 0.306 | 0.746 | 0.128 | 0.300 | 0.355 | 0.709 | 0.871 | 0.201 | 0.172 | NA | 0.745 |
| Triglyceride | 0.475 | 0.326 | 0.338 | 0.567 | 0.920 | 0.306 | 0.746 | 0.128 | 0.300 | 0.355 | 0.709 | 0.871 | 0.201 | 0.172 | 0.255 | 0.745 |
| CRP | 0.391 | 0.500 | 0.149 | 0.541 | 0.733 | 0.277 | 0.312 | 0.345 | 0.106 | 0.289 | 0.564 | 0.941 | 0.208 | 0.276 | 0.516 | 0.887 |
| Hb A1c | 0.475 | 0.343 | 0.707 | 0.998 | 0.841 | 0.202 | 0.812 | 0.452 | 0.393 | 0.406 | 0.882 | 0.747 | 0.201 | 0.187 | 0.267 | NA |

Supplementary table: Predictors of missingness matrix

Note. * p < 0.05. Shaded cells indicate significant predictors of missingness.
